# Time-dynamic associations between symptom-related expectations, self-management experiences and somatic symptom severity in everyday life: an ecological momentary assessment study with university students

**DOI:** 10.1136/bmjopen-2024-091032

**Published:** 2025-02-07

**Authors:** Stefanie Hahn, Yvonne Nestoriuc, Simon Kirchhof, Anne Toussaint, Bernd Löwe, Franz Pauls

**Affiliations:** 1 Clinical Psychology and Psychotherapy, Helmut-Schmidt-University / University of the Armed Forces Hamburg, Hamburg, Germany; 2 Systems Neuroscience, Universitätsklinikum Hamburg-Eppendorf, Hamburg, Germany; 3 Department of Developmental and Educational Psychology, Helmut-Schmidt-University / University of the Armed Forces Hamburg, Hamburg, Germany; 4 Department of Psychosomatic Medicine and Psychotherapy, Universitätsklinikum Hamburg-Eppendorf, Hamburg, Germany

**Keywords:** MENTAL HEALTH, Somatoform Disorders, PUBLIC HEALTH

## Abstract

**Abstract:**

**Objective:**

The present study investigated the associations between symptom-related expectations, self-management experiences and expectation framing on somatic symptom severity in university students in two conditions (positive or standard expectation framing). We hypothesised that symptom-related expectations are significantly associated with concurrent and subsequent levels of somatic symptom depending on expectation framing.

**Design:**

A smartphone-based micro-longitudinal ecological momentary assessment study with randomisation to one of two expectation framing groups (positive vs negative) was carried out. Multilevel mixed-effects linear regression analyses were conducted for data analysis.

**Setting:**

Data was collected in real-time from university students via smartphones, with three predetermined assessments per day over seven consecutive days.

**Participants:**

A total of 104 students (63.5% male, 0% diverse) who were 18 years or older, possessing sufficient German language skills and had access to an Android-powered smartphone were included.

**Interventions:**

Participants were randomised to one of two different expectation framing groups, either receiving questionnaires for the expected impairment due to somatic symptoms (negative framing) or for the expected freedom from impairment due to somatic symptoms (positive framing).

**Primary outcome measures:**

Somatic symptom severity was assessed using an adapted version of the Patient Health Questionnaire, with 11-point instead of 3-point Likert-scales. Symptom-related expectations were assessed using 11-point Numerical Rating Scales and self-management experiences were assessed using binary variables.

**Results:**

Concurrent analysis revealed a significant association between symptom-related expectations and symptom severity (β=0.934, p<0.001), but no significant associations between self-management experiences and symptom severity. Regarding expectation framing, participants in the negative group reported higher symptom severity levels than those in the positive group (β=−0.071, p<0.001). Results indicated a stronger association between symptom-related expectations and symptom severity in the negative framing group (β=−0.088, p<0.001). Time-lagged analysis showed higher levels of symptom-related expectations predicted higher subsequent symptom severity levels (β=0.502, p<0.001), whereas preceding symptom severity levels or self-management experiences did not predict subsequent symptom severity levels. Negative framing was associated with higher subsequent symptom severity levels (β=−0.158, p<0.001). The effect of symptom-related expectations on subsequent symptom severity levels was independent of expectation framing.

**Conclusions:**

Our findings highlight the impact of expectations and expectation framing on somatic symptom severity among university students and expand the knowledge needed for the development of expectation management techniques.

**Trial registration number:**

ISRCTN36251388.

**STRENGTHS AND LIMITATIONS OF THIS STUDY**

- The data collection took place in real-time and real life, as ecological momentary assessment captures participants’ experiences in their everyday life, thus reducing recall bias.
- The study included multiple assessments per day and thus provided thorough data on the course of symptom-related expectations and somatic symptom severity.
- Multilevel modelling was used for data analysis, which, as a robust analytical approach, accounts for individual differences, repeated assessments and missing data.
- The study did not account for individual stress levels, environmental, interpersonal or university-specific stressors that may also influence somatic symptom severity.
- The sample consisted of university students and may provide limited generalisability due to potential sampling biases.

## Introduction

 Somatic symptoms such as pain, gastrointestinal complaints, lack of energy or fatigue are ubiquitous in the general population. Research indicates that somatic symptoms can be a major source of distress and various physical dysfunctions.^1 2^ Female gender, low socioeconomic status and obesity have been identified as significant risk factors for persistent somatic symptoms in previous studies.^3 4^ Persistence and lacking treatment may contribute to maladaptive thoughts, feelings or behaviours related to somatic symptoms or health concerns. This could potentially progress into clinically relevant conditions such as somatic symptom disorder (SSD).^5^,^8^ While the overall prevalence of somatic symptoms increases with age,^3^ young adults are also likely to experience elevated levels of somatic complaints . Reasons for this may include psychological distress, biological factors (eg, high cortisol, low heart-rate variability) and environmental factors (eg, overprotective parenting style).^9^,^11^ Especially young adults in academically challenging environments, appear to be at high risk of experiencing somatic symptoms. Studies highlight that headaches, abdominal pain or fatigue are experienced by more than 20% of college students and 67% of high school students.^12^,^14^ Experiencing somatic symptoms in an academic environment is associated with psychological distress, an increased risk of university dropouts and poor levels of functioning at advanced academic levels.^12^ Aetiological models in psychosomatic medicine suggest that expectations and prior experiences may play a substantial role in the development and perception of somatic symptoms and thus contribute to symptom course and potential chronification.^15^ Expectations related to future somatic symptoms (symptom-related expectations) as well as prior treatment experiences may thus act as contributing factors for the perceived levels of somatic symptom severity. Additionally, current research studies using ambulatory assessment approaches (such as ecological momentary assessment) suggest negative affectivity, negative psychological factors and stress to play an integral role in the development of medically unexplained symptoms in both clinical and healthy samples.^16 17^ However, despite the debilitating nature of somatic symptoms on the psychological and academic functioning of young adults, there is a gap regarding knowledge on the course and persistence of somatic symptoms in this population. Here, little is known about the short-term course of physical concerns and moment-to-moment fluctuations of symptom severity.

### Symptom-related expectations and prior treatment experiences

In general, symptom-related expectations can be conceptualised as future-targeted cognitions that enable the anticipation of future symptom severity or bodily states.^18^ These types of expectations have been shown to predict long-term symptom course for a wide range of somatic symptoms related to heart rate, lung function, blood pressure or bowel motility, among others.^19^ There is growing evidence that a positive modification of expectations may improve clinical outcomes in different medical and psychological conditions.^20^,^23^ Here, placebo and nocebo effects play an integral role. Such effects can be understood as being induced by scham interventions (inert or active) that can still cause significant effects in the sense of worsening (nocebo) or improving (placebo) symptoms or conditions by manipulating the information context (eg, framing).^24^ Thus, symptom-related expectations may cause placebo responses (reducing future somatic symptom severity) but may also induce nocebo effects (increasing future somatic symptom severity).^20 25^ Since there is a lack of knowledge regarding the short-term influence of symptom-related expectations on somatic symptom severity, further investigation is warranted to clarify this relationship.

For persistent somatic symptoms (PSS), a possible effect of symptom-related expectations might thus depend on the type of expectation or its frame of reference (expectation framing).^26 27^ Research has already shown that framing can affect perceptual experiences (eg, weight estimations) or influence opinions on important matters (eg, politics).^28 29^ Framing effects may thus depend on factors such as perceived importance or context.^29 30^ It is yet to be determined whether negatively framed expectations of future symptom impairment and positively framed expectations of symptom-free periods differ in their impact on future reported somatic symptom severity.

Besides symptom-related expectations, prior treatment experiences may also contribute to the development and maintenance of somatic symptoms. In the context of psychosomatic disorders, the role of prior treatment experiences is highlighted in terms of negative interactions with the healthcare system in the past (eg, frustration caused by inappropriate treatments).^15^ Since such experiences can act as aggravating or maintaining factors for somatic symptoms in individuals with clinical conditions, treatment experiences might influence somatic symptom severity in the general population as well. However, as most somatic symptoms that are experienced in everyday life among the general population do not have an explicit link to a clinical condition, they may not necessarily be accompanied by healthcare use.^31^ In the context of this study, treatment experiences were therefore defined as experiences related to the individual self-management of somatic symptoms (eg, self-medication, physical activity and protective behaviour).

### Objective

The knowledge of factors that may influence somatic symptom severity in the general population needs further research. As of now, potential factors include a multitude of potentially relevant mechanisms (eg, infections, sleep problems, life events).^32^ The present study thus aims to provide a deeper insight into contributing factors for moment-to-moment fluctuations of somatic symptom severity, experienced by university students. Specifically, the role of symptom-related expectations and expectation framing on somatic symptom severity will be explored by investigating whether symptom severity depends on the positive or negative framing of symptom-related expectations. Furthermore, the role of self-management experiences in somatic symptom severity will be addressed. We assume that negatively framed expectations are strongly associated with higher average levels of somatic symptom severity while positively framed expectations are strongly associated with lower average levels of somatic symptom severity. We expect that the association between symptom-related expectations and perceived levels of somatic symptom severity will be more pronounced in students with negatively framed expectations than in those with positively framed expectations. Furthermore, we investigate whether concurrent symptom-related expectations predict subsequent levels of symptom severity (ie, reported symptom severity at the next measurement point). We explore whether subsequent levels of somatic symptom severity are significantly associated with expectation framing and assume a strong association between negatively framed expectation and higher levels of subsequent symptom severity. Conversely, we expect a strong association between positively framed expectations and lower levels of subsequent somatic symptom severity. We assume that a possible effect of symptom-related expectations on subsequent levels of somatic symptom severity will be larger in students with negatively framed expectations than in those with positively framed expectations. Furthermore, we investigate whether self-management experiences and concurrent symptom severity levels predict subsequent levels of symptom severity.

## Methods

### Patient and public involvement

Patients or other public members were not involved in the conceptualisation of the present study.

### Procedure

University students from the Helmut-Schmidt-University/University of the Federal Armed Forces in Hamburg, Germany were recruited via university-internal emails and mailing lists, online advertisements and digital flyers that included brief study descriptions and contact information. Interested students were required to send a request via email and were included if they fulfilled the following eligibility criteria: being 18 years or older, having sufficient German language skills and being in possession of an Android-powered smartphone. After a comprehensive written explanation of the study procedure had been given to eligible students, they provided written informed consent. Subsequently, all participants filled out a self-report online questionnaire to provide further socio-demographic information (eg, age, gender, body mass index, migration background, marital status and the number of medical consultations within the prior 4 weeks and 6 months). Participants did not receive financial reimbursement but were credited with three test subject hours for a fully completed participation in the study.

### Study design

**Figure 1:**
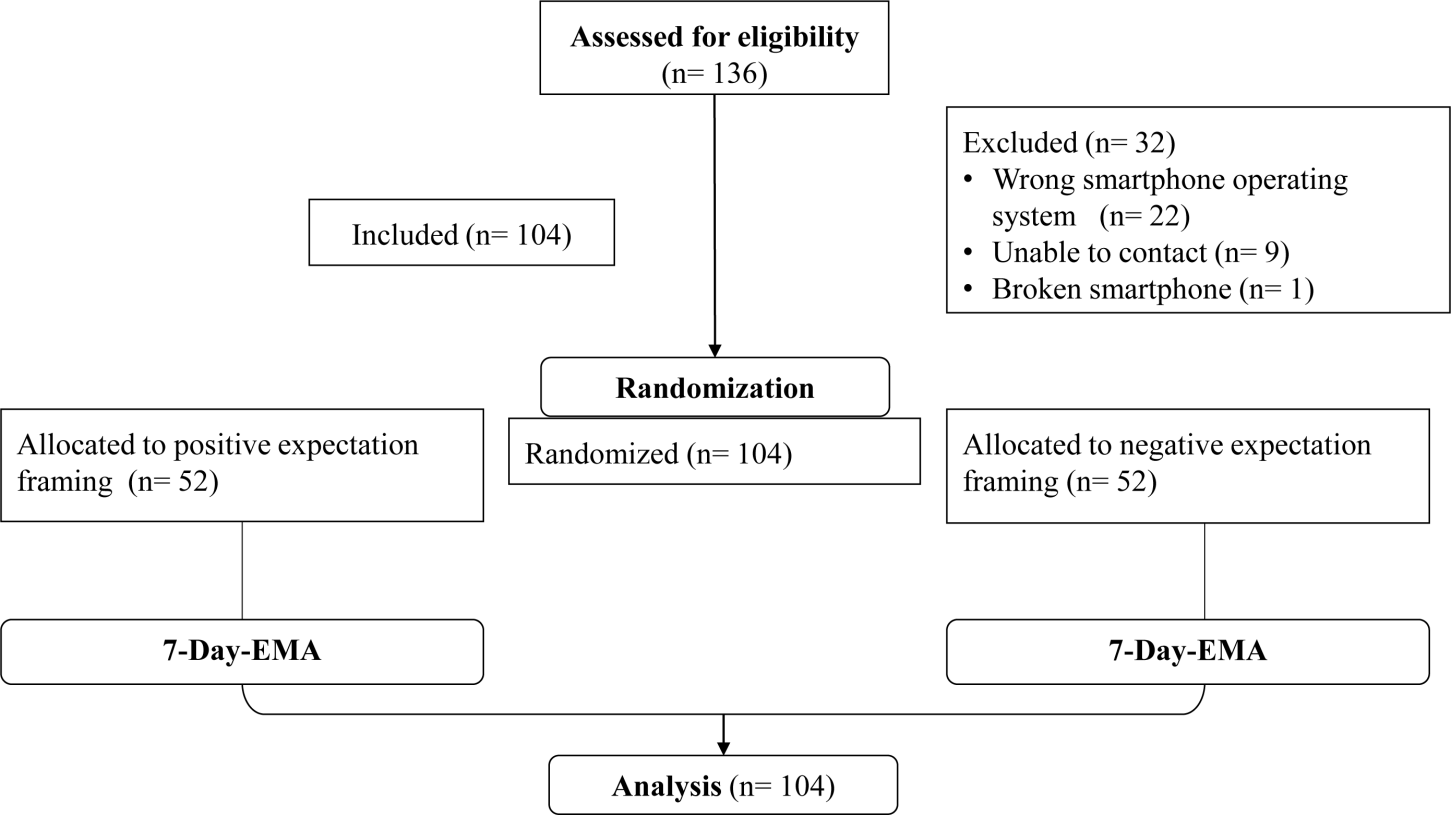
Consolidated Standards of Reporting Trials flow diagram of participants throughout the course of the study. Note. The EMA includes three assessment points per day (21 assessment points in total), each with questionnaires administered at predefined times (08:00, 12:00 and 18:00). In the positive expectation framing group, the questionnaires administered refer to the expected freedom from impairment due to somatic symptoms. In the negative expectation framing group, the questionnaires administered refer to the expected impairment due to the anticipated somatic symptoms. EMA, ecological momentary assessment.

A 7-day ecological momentary assessment (EMA) study was conducted to investigate the presented research objectives. EMA enables a reliable assessment of time-dynamic fluctuations of symptom severity and other-related factors in everyday life^33^ and allows for data collection about real-time processes of somatic symptoms rather than memory-based summary ratings. This approach may increase ecological validity^34 35^ by reducing retrospective and heuristic biases, and has already been successfully employed in previous studies on somatic symptom experience.^36^,^39^ The present smartphone-based EMA study followed a micro-longitudinal cohort design. Participants were randomly allocated to one of two expectation framing groups (positive or negative expectation framing) and provided with a written instruction to instal the customised smartphone-based application movisensXS (Movisens GmbH). Random group allocation was ensured by providing participants with random group-specific QR codes for study registration. Prior to assessment, the same number of QR codes had been created for each framing group and were randomly distributed among participants in predefined alternating order. Once registered to the study, participants received questionnaires via the application, which signalled the beginning of each predetermined assessment (08:00, 12:00 and 18:00) by automated visual and auditory signals. Questionnaires regarding somatic symptom severity (Patient Health Questionnaire (PHQ)_adapt_) and self-management experiences (NRS_treat_) were the same for both groups, while questionnaires regarding symptom-related expectations (Numerical Rating Scale (NRS)_expectNeg_ vs NRS_expectPos_) depended on their group membership. Thus, participants in the negative expectation framing group received questionnaires for the expected impairment due to somatic symptoms and participants in the positive expectation framing group received questionnaires for the expected freedom from impairment due to somatic symptoms. For further details, please refer to the questionnaires in the appendix. The study took place over seven consecutive days and consisted of 21 assessment points. Each questionnaire took approximately 5 min to complete. At each survey time, participants had up to 2 hours to complete a questionnaire and received automated half-hourly reminders within this time frame. A detailed overview of the flow of participation and the course of the study is presented in figure 1.

All questionnaires were implemented using the software package movisensXS (Movisens GmbH). Pseudonymised data was temporarily stored by the company Movisens GmbH in the data centre of TelemaxX Telekommunikation GmbH. Post data collection, the data was transferred to the Chair of Clinical Psychology and Psychotherapy at the Helmut-Schmidt-University in Hamburg and was deleted from the cache afterwards. An identification of personal data was not possible at any point. Data protection and privacy declaration were evaluated based on the General Data Protection Regulation of the European Parliament and approved by the data protection officer of the Helmut-Schmidt-University/University of the Federal Armed Forces in Hamburg, Germany on 07 April 2022. This study is part of the study ‘Modifiable Factors for Somatic Symptom Persistence in Patients with Somatic Symptom Disorder’, with the goal of piloting the applicability of an EMA study design for the assessment of symptom-related expectations. All study procedures were approved by the ethics committee of the Medical Chambers Hamburg, Germany (reference number: 2020–10197-BO-ff, 25 January 2021).

### Instruments and measures

Somatic symptom severity was assessed using the PHQ-15.^31 40^ The PHQ-15 assesses the perceived severity of 15 different somatic symptoms during the past 4 weeks. It accounts for up to 90% of the most frequently reported somatic symptoms of patients (eg, low energy, headache, constipation). The severity of each somatic symptom is rated on a 3-point NRS (0=‘not bothered at all’, 1=‘bothered a little’, 2=‘bothered a lot’). The total sum score ranges from 0 to 30. It is one of the best-validated questionnaires for the assessment of somatic symptom severity and shows high levels of internal consistency for the English and German versions alike (English: Cronbach’s α=0.80, German: Cronbach’s α=0.79).^24 32^ For the purpose of this study, the scale of the original PHQ-15 was extended to a unidimensional 11-point NRS. In our adapted version PHQ_adapt_, ratings for each somatic symptom range from 0 (‘not bothered at all’) to 10 (‘bothered a lot’) and refer to the currently perceived impairments (eg, ‘At the moment, how much are you bothered by stomach pain?’). This enables the calculation of a broader range of total sum scores for each assessment point, ranging from 0 to 150 and thus allows for a detailed assessment of minor fluctuations in symptom severity on a daily basis.

To assess symptom-related expectations, participants in the negative expectation framing group were asked to rate their expected symptom severity on each of the 15 somatic symptoms of the PHQ_adapt_ on an 11-point NRS_expectNeg_, ranging from 0 (‘not bothered at all’) to 10 (‘bothered a lot’). Participants in the positive expectation framing group rated their expected symptom-free periods on each of the 15 somatic symptoms of the PHQ_adapt_ on a unidimensional 11-point NRS_expectPos_, ranging from 0 (‘not unbothered at all’) to 10 (‘highly unbothered’). Self-ratings of negatively framed expectations (eg, ‘How much do you expect to be bothered in terms of stomach pain by noon?’) and positively framed expectations (eg, ‘How much do you expect to be unbothered by noon in terms of stomach pain?’) both referred to the respective next assessment point. The total sum scores of the NRS_expectNeg_ and NRS_expectPos_ range from 0 to 150 for each assessment point. To provide a unidimensional variable for symptom-related expectations in both expectation framing groups, the scale of NRS_expectPos_ was recoded to match the scale of NRS_expectNeg_ by reversing scale polarity prior to the calculation of total sum scores.

Following each NRS_expectNeg_ or NRS_expectPos_ item, self-management experiences were assessed through a binary item (0=‘no’, 1=‘yes’) for each of the 15 somatic symptoms of the PHQ_adapt_, referring to the timespan from the last assessment point to the current one (eg, ‘Have you taken any action since last evening to treat the stomach pain?’). For each assessment point, total sum scores for self-management experiences ranged from 0 to 15 and described the total number of individual self-management interventions for short-term somatic symptoms per assessment point. For further details, please refer to the questionnaires in the appendix.

### Data analyses

An a priori power analysis that was based on a medium effect size, an alpha error of α=0.05, a statistical power of 1-β=0.80 and four predictor variables (symptom-related expectations, expectation framing, self-management experiences and the interaction between expectations and experiences) suggested a required total sample size of N=85 for the variance in the criterion variable (somatic symptom severity) that is explained by the specified regression model to be different from zero. Data preparation and all required data analyses were carried out using IBM SPSS Statistics software V.29.^41^ Statistical significance was evaluated based on an alpha level of 0.05. To examine socio-demographic and healthcare-related differences between both expectation framing groups, we conducted t-tests for continuous and parametric as well as χ^2^ tests for categorical or non-parametric data, using listwise deletion of occasional missing data. Χ^2^ tests of homogeneity were conducted to test whether the frequency distributions of further socio-demographic variables differed between the two expectation framing groups. All hypotheses were tested using hierarchical linear modelling on the data gathered within the 7-day EMA. The corresponding data set consisted of a maximum of 21 assessments×104 persons, including 2184 observations in total. Intraclass correlation coefficients (ICC) were computed and used as an indicator of the proportion of variance explained by the different levels. ICC was thus used to determine whether hierarchical linear modelling was a useful analytical approach.

Multilevel mixed-effects linear regression analyses (multilevel linear modelling, MLM) were conducted to investigate the concurrent and subsequent associations between symptom-related expectations, expectation framing, self-management experiences and somatic symptom severity on the within-person level. MLM is deemed suitable for analysing hierarchical and extensive longitudinal data including multiple repeated measures, as it accounts for the dependence between observations and accommodates missing observations using the robust maximum likelihood estimation technique.^42 43^ The hierarchical structure of the present EMA data is defined by two levels: observations (level 1) nested within individuals (level 2). Variables at level 1 may vary across assessment points (eg, symptom-related expectations), while variables at level 2 may vary across persons (eg, age). Prior to MLM, total scores on the predictor variables were mean-centred to reduce the potential risk of multicollinearity between predictor variables and the constructed cross-product term (interaction term). Given that expectation framing was defined as a binary group variable, this predictor variable was effect-coded (1=‘negative expectation framing group’; 2=‘positive expectation framing group’). The slopes and intercepts of within-person predictors were allowed to randomly vary across individuals. The criterion variable was operationalised by the total sum scores on the PHQ_adapt_ (somatic symptom severity) for each assessment point. Total sum scores on the NRS_expect_ (symptom-related expectations), expectation framing, the NRS_treat_ (self-management experiences) and the constructed cross-product term for the interaction between symptom-related expectations and expectation framing were used as the respective predictor variables. Concurrent associations between the criterion variable somatic symptom severity and the predictor variables symptom-related expectations, self-management experiences, expectation framing and the interaction between symptom-related expectations and expectation framing were analysed by including all variables at the same assessment points (*t*) in the first MLM (MLM 1). For investigating the potential effects of the predictor variables on subsequent somatic symptom severity, an additional MLM (MLM 2) was performed. In MLM 2, a time-lagged analysis was conducted based on the associations between somatic symptom severity at a given assessment point (*t*) and somatic symptom severity, symptom-related expectations, self-management experiences, expectation framing and the interaction between symptom-related expectations and expectation framing at the previous assessment point (*t*-1).

## Results

### Participants and sample characteristics

All analyses were based on the data of N=104 participants. The final sample consisted of 63.5% male participants (0% diverse). The most frequently reported somatic symptoms throughout the 7-day EMA were lack of energy (22.3%), headache (12.7%) and back pain (11.2%). The expectation framing groups were compared regarding the most relevant socio-demographic and healthcare-related variables to test for any group differences that might affect the results of subsequent analyses. There were no group differences regarding socio-demographic and healthcare-related variables except for the PHQ_adapt_ items ‘Feeling heart pound or race’ (*t* (102)=−1.616, p=0.044) and ‘Shortness of breath’ (*t* (102)=−1.432, p=0.006). Socio-demographic and healthcare-related characteristics of the total sample are depicted in tables1  2. For more information on socio-demographic and healthcare-related characteristics as well as group comparisons for the measures of symptom severity and symptom-related expectations for the expectation framing groups, please refer to online supplemental tables a and b. The average completion rate of the EMA assessments ranged between 84.9% for participants in the negative expectation framing group (Min=8.7%, Max=100%) and 82.6% for participants in the positive expectation framing group (Min=17.4%, Max=100%), resulting in 1829 valid observations across both groups. The average level of missingness in the EMA data across both groups was 16.3% (Min=0%, Max=91.3%). There was no significant difference regarding the compliance rate between both expectation framing groups when comparing the number of valid assessments between both groups (*t* (102)=0.648, p=0.519).

**Table 1:** Socio-demographic characteristics of the total sample

Variable	Total sample (N=104)			
	n (%)	M	SD	Min/Max
Gender	104 (100)			1/2
Male (1)	66 (63.5)			
Age (in years)	104 (100)	24.27	2.70	19/33
Educational level	104 (100)			1/2
Higher (1)	50 (48.1)			
Migration background	104 (100)			0/1
With (1)	16 (15.4)			
Partnership	104 (100)			0/1
Yes (1)	70 (67.3)			
Medical consultations (4 weeks)	51 (49.0)	0.87	1.25	0/7
Medical consultations (6 months)	90 (86.5)	3.26	3.61	0/15

Note. Educational level is defined as the highest level of education achieved by the participant (higher (1) = ‘working towards a bachelor’s degree’, highest (2) = ‘working towards a master’s degree’). Migration background is indicated when either the participant or at least one parent was not born in Germany. For the sake of readability, descriptive statistics for only one category are depicted for the variables Ggender, Eeducational Bbackground, Mmigration Bbackground, and Ppartnership. Test statistics for group comparisons are based on all categories of the respective variables. Medical consultations (4w weeks)=total number of medical consultations that had been attended within the last four4 weeks. Medical consultations (6m months)=total number of medical consultations that had been attended within the last six6 months.

**Table 2:** Healthcare-related characteristics of the total sample

Somatic symptom	Total sample (n=104)			
	n (%)	M	SD	Sum (Min/Max)
Stomach pain	45 (43.3)	1.71	3.25	178 (0/18)
Back pain	71 (68.3)	4.46	5.73	464 (0/21)
Pain in arms, legs or joints (Knees, hips, etc)	65 (62.5)	4.27	5.69	444 (0/20)
Menstrual cramps or other problems with your period	14 (13.5)	0.36	1.32	37 (0/10)
Headaches	82 (78.8)	5.05	5.61	525 (0/20)
Chest pain	30 (28.8)	1.10	3.40	114 (0/21)
Dizziness	44 (42.3)	1.41	2.94	147 (0/21)
Fainting spells	11 (10.6)	0.17	0.76	18 (0/7)
Feeling heart pound or race	40 (38.5)	1.44	3.30	150 (0/21)
Shortness of breath	34 (32.7)	1.87	4.54	194 (0/21)
Pain or problems during sexual intercourse	17 (16.3)	0.53	1.87	55 (0/12)
Constipation, loose bowels or diarrhoea	56 (53.8)	2.50	4.29	228 (0/21)
Nausea, gas or indigestion	64 (61.5)	2.90	4.29	299 (0/21)
Feeling tired or having low energy	91 (87.5)	8.79	7.21	922 (0/21)
Trouble sleeping	59 (56.7)	3.37	5.10	336 (0/21)
Sum of somatic complaints	103 (99.1)	39.72	38.13	4131 (0/215)

Note. Somatic symptoms are operationalizedoperationalised by the total sum score of 15 unidimensional 11-point nNumerical rRating sScales (PHQ_adapt_ ranging from 0 = ‘not impaired at all’ to 10 = ‘very strongly impaired’). Scores were coded as binary (0=no somatic complaints complaints, 1=somatic complaints complaints) for the frequencies of somatic symptoms shown. N refers to the total number of reported somatic complaints complaintsfor the specific symptom over a 7--dayday period. Min/Max describes the days on which the least/most somatic complaints complaintswere reported.

PHQPatient Health Questionnaire

### Multilevel linear modelling

MLM 1 and MLM 2 were both supported by the ICC, indicating that a large proportion of the total variance in the criterion variables of both models was attributable to the between-group variability (ICC_MLM 1_=0.449; ICC_MLM 2_=0.586).

As presented in table 3, the results of MLM 1 indicated a significant and large positive main effect of symptom-related expectations on concurrent somatic symptom severity (β=0.934, p<0.001). Thus, higher expectations regarding symptom impairment were strongly associated with higher concurrent levels of somatic symptom severity. Moreover, a rather small but significant negative main effect was found for expectation framing on concurrent somatic symptom severity (β=−0.071, p<0.001). Conclusively, participants in the negative expectation framing group reported slightly but significantly higher average levels of concurrent somatic symptom severity than those in the positive expectation framing group. Additionally, the interaction between symptom-related expectations and expectation framing (expectation×framing) was significantly associated with concurrent somatic symptom severity (β=−0.088, p<0.001). This indicates that symptom-related expectations were more strongly associated with concurrent average levels of somatic symptom severity in the negative expectation framing group than in the positive expectation framing group. No meaningful association could be found between self-management experiences and concurrent somatic symptom severity.

**Table 3:** Multilevel mixed-effects linear regression analysis for the associations with concurrent levels of somatic symptom severity as the criterion variable

Variable	MLM 1: concurrent associations					
	β	B	SE (B)	*t*	P value	95% CI (B)
Symptom-related expectations	0.934	0.957	0.018	52.880	<0.001	(0.922, 0.992)
Expectation framing	−0.071	−1.273	0.235	−5.419	<0.001	(−1.734, 0.812)
Expectation×framing	−0.088	−0.180	0.036	−4.920	<0.001	(−0.251, 0.108)
Self-management experiences	0.003	0.059	0.098	0.607	0.544	(−0.132, 0.251)

Note. Symptom-related expectations are operationalizedoperationalised by the total sum score of 15 unidimensional 11-point nNumerical rRating sScales (NRS_expect_ ranging from 0 = ‘not impairment free at all’ to 10 = ‘highly impairment free’). Expectation framing=expectations framing group (1 = ‘negative expectation framing group’, 2 = ‘positive expectation framing group’). Expectation×framing=cross-product term for the interaction between the predictor variables symptom-related expectations and expectation framing. Self-management experiences are defined as the total number of individual self-management interventions of short-term somatic symptoms per assessment point and measured by the total sum score of 15 binary rating items (NRS_treat_ with 0 = ‘no’, 1 = ‘yes’). Somatic symptom severity (criterion variable) is operationalized operationalised by the total sum score of 15 unidimensional 11-point nNumerical rRating sScales (PHQ_adapt_ ranging from 0 = ‘not bothered at all’ to 10 = ‘bothered a lot’).

MLMmultilevel linear modellingPHQPatient Health Questionnaire

The results of the time-lagged analysis (MLM 2) depicted in table 4 revealed a significant positive main effect of symptom-related expectations on somatic symptom severity experienced at the subsequent assessment points (β=0.502, p<0.001). This indicated that higher levels of symptom-related expectations predicted higher levels of later experienced somatic symptom severity. As in the case of the concurrent associations found in MLM 1, expectation framing turned out to be negatively associated with subsequent somatic symptom severity in MLM 2 (β=−0.158, p<0.001). On average, participants in the negative expectation framing group reported significantly higher levels of later experienced somatic symptom severity than those in the positive expectation framing group. In contrast to the concurrent associations found in MLM 1, the interaction between symptom-related expectations and expectation framing (expectation×framing) was not found to significantly affect subsequent somatic symptom severity in MLM 2. Moreover, results of MLM 2 indicated that participants’ self-management experiences and prior experienced levels of somatic symptom severity did not have a statistically significant effect on their later experienced somatic symptom severity.

**Table 4:** Multilevel mixed-effects linear regression analysis for the time-lagged associations with somatic symptom severity as the criterion variable

Variable	MLM 2: time-lagged associations					
	β	B	SE (B)	*t*	P value	95% CI (B)
Symptom-related expectations	0.502	0.501	0.088	5.755	<0.001	(0.333, 0.678)
Expectation framing	−0.158	−2.767	0.679	−4.074	<0.001	(−4.103, 1.430)
Expectation×framing	−0.060	−0.121	0.109	−1.117	0.267	(−0.337, 0.094)
Self-management experiences	−0.017	−0.248	0.272	−0.912	0.362	(−0.782, 0.286)
Somatic symptom severity (prior)	−0.124	−0.122	0.085	−1.438	0.151	(−0.289, 0.045)

Note. Symptom-related expectations are operationalizedoperationalised by the total sum score of 15 unidimensional 11-point nNumerical rRating sScales (NRS_expect_ ranging from 0 = ‘not impairment free at all’ to 10 = ‘highly impairment free’). Expectation framing=expectations framing group (1 = ‘negative expectation framing group’, 2 = ‘positive expectation framing group’). Expectation×framing=cross-product term for the interaction between the predictor variables symptom-related expectations and expectation framing. Self-management experiences are defined as the total number of individual self-management interventions of short-term somatic symptoms per assessment point and measured by the total sum score of 15 binary rating items (NRS_treat_ with 0 = ‘no’, 1 = ‘yes’). Somatic symptom severity (prior) is operationalizedoperationalised by the total sum score of 15 unidimensional 11-point nNumerical rRating sScales (PHQ_adapt_ ranging from 0 = ‘not bothered at all’ to 10 = ‘bothered a lot’) and refers to the experienced impairments at the previous assessment points. The criterion variable is defined as somatic symptom severity experienced at the subsequent assessment points.

MLMmultilevel linear modellingPHQPatient Health Questionnaire

To consolidate the present findings, sensitivity analyses were conducted to address gender as a potential covariate and the influence of statistical outliers (ie, 2 SD above the mean level of symptom severity) on the effects found in MLM 1 and MLM 2. Analysis revealed no significant gender differences in symptom-related expectations, self-management experiences and symptom severity. Most of the effects indicated by MLM 1 and MLM 2 could be replicated in the sensitivity analysis after excluding statistical outliers. Although this was true for all effect sizes in MLM 1, however, the effect of symptom-related expectations on subsequent levels of symptom severity in MLM 2 significantly decreased after excluding statistical outliers (*t*(ΔB)=11.25, p<0.001. For details, please refer to online supplemental tables c and d.

## Discussion

Despite the prevalence of somatic symptoms among individuals in academia, there is still a limited understanding about factors influencing symptom severity in non-clinical populations. Therefore, the present study was set out to investigate associations between symptom-related expectations, self-management experiences, expectation framing and somatic symptom severity experienced by university students through the implementation of EMA as an innovative approach for the assessment of real-time and real-life experiences. Results revealed a link between concurrent symptom-related expectations and somatic symptom severity, with higher average levels of symptom severity reported by those in the negative expectation framing group. Time-lagged analysis suggested that higher levels of symptom-related expectations may predict higher subsequent levels of symptom severity, irrespective of previously experienced symptom severity or self-management experiences. Negative expectation framing was associated with increased levels of subsequent symptom severity.

### Concurrent associations

In line with our assumption, MLM 1 indicated a strong positive association between symptom-related expectations and concurrent somatic symptom severity. More severe somatic symptoms thus appear to be strongly linked to higher expectations regarding future symptom impairment. This relationship might reflect a strong influence of current somatic symptoms on expectations regarding the course of future symptom severity. Contrary to our assumptions, self-management experiences were not found to be significantly associated with concurrent levels of somatic symptom severity. However, only a small number of self-management interventions were reported in this sample, with 21% of the participants reporting no interventions at all and the mean number of reported self-management interventions over 7 days being 0.23 (SD=0.37). Here, operationalising self-management experiences by the total number of individual interventions per assessment point might underestimate the true associations between self-management experiences and somatic symptom severity. Furthermore, other factors could have contributed to the absence of a considerable link between self-management experiences and somatic symptom severity. First, time variations in the onset of effectiveness of self-management interventions were not considered in the present study. For example, non-prescription painkillers (eg, ibuprofen) can take longer than 30 min to reach full effect,^44^ while physical activities, such as stretching exercises, can be beneficial immediately after application.^45^ Thus, it is plausible to assume that some effects of self-management experiences were not observed in the concurrent analysis. A qualitative symptom-specific investigation of self-management effects should thus be conducted to further analyse the potential effects of specific self-management interventions on somatic symptoms.

Results of MLM 1 further indicated a small but significant negative main effect of expectation framing on somatic symptom severity, confirming our assumption that negative expectation framing may be associated with higher concurrent levels of somatic symptom severity. Moreover, a negative interaction effect between symptom-related expectations and expectation framing suggests that the positive association between symptom-related expectations and somatic symptom severity was slightly more pronounced for participants in the negative expectation framing group than for those in the positive expectation framing group. These findings support the notion that nocebo effects may play an important role in the perception and exacerbation of prevalent physical complaints, particularly over a short period of time. Reasons may be partly attributed to selective attentional focus, where thoughts are directed towards physical exhaustion.^46 47^ Individuals might hyper-focus on sensations of somatic symptoms (eg, fatigue), leading to an amplification of perceived discomfort. Additionally, confirmation bias, (eg, the belief ‘I woke up feeling tired already, this will surely persist throughout the day,’) might contribute to the intensification of interoceptive signals or symptoms that were relatively weak in the beginning.^48^ In clinical contexts, for example, functional correlates have indicated a different experience of pain in patients diagnosed with SSD compared with healthy individuals (eg, increased functional connectivity between the sensorimotor network and default mode network),^49^ potentially making them more susceptible to the influence of negative expectations and beliefs. The results of the present study appear to support this assumption, suggesting that even in healthy individuals, nocebo effects can influence the perception and development of physical complaints over a short time span. Based on neuroscientific concepts, processes of predictive coding^50^ may be discussed. In the case of PSS, it has been demonstrated that strong priors (eg, the repeated experience of fatigue throughout the day after waking up tired) can over-ride weaker sensory inputs from the body.^51^ The present results suggest that this may also be the case on a short-term level, leaving room for further research.^52^ While MLM 1 suggests a stronger link between negative expectation framing and somatic symptom severity, possible placebo effects could also be suggested as an explanation for the lower average levels of somatic symptom severity found in the positive expectation framing group.

### Time-lagged associations

Contrary to our assumption, MLM 2 indicated that subsequent levels of somatic symptom severity were not significantly predicted by preceding levels of symptom severity. Since the participating university students were overall healthy, it seems reasonable to assume that the types and severity levels of their perceived physical complaints were highly fluctuating across multiple assessment points, thus being dynamically changing rather than being persistent. Short-term physical complaints could thus be influenced more strongly by external factors, such as weather conditions, sleep quality, nutrition or exercise.^4553^,^55^

The time-lagged analysis suggested symptom-related expectations as a significant predictor for subsequent somatic symptom severity, thus supporting our assumption. Symptom-related expectations may induce a nocebo effect, thus increasing later experienced levels of somatic symptom severity. However, MLM 2 failed to indicate self-management experiences as a significant predictor for subsequent somatic symptom severity. Due to the limited number of participants engaging in such self-management interventions and not considering further qualitative aspects, additional research is warranted to further explore the potential effects of self-management experiences on future somatic symptom severity. Expectation framing was found to be a significant predictor for subsequent somatic symptom severity. Contrary to MLM 1, however, MLM 2 failed to indicate a significant interaction effect between expectation framing and symptom-related expectations on subsequent somatic symptom severity. The predictive power of symptom-related expectations on somatic symptom severity thus appeared to be independent of whether expectations were negatively or positively framed. A possible explanation might relate to potential differences in the latencies of placebo and nocebo effects on subsequent levels of somatic symptom severity. In the negative expectation framing group, cognitive restructuring processes might have played a minor role at best, as the concept of expectations regarding future symptom impairment is highly congruent with the currently perceived levels of somatic symptom severity. In the positive expectation framing group, however, the input would have been contradictory as expectations regarding future symptom relief would conflict with present somatic complaints. Since the cognitive processing of contradictory or incongruent stimuli takes longer,^56^ it is possible that placebo responses would emerge in time-lagged analysis while nocebo responses would be more apparent in concurrent analysis.

### Limitations and implications

As previous research has demonstrated, university students show a higher risk of developing somatic symptoms due to academic pressure and stress. Despite confirming the important role of symptom-related expectations and expectation framing for somatic symptom severity, the present study failed to consider the individual stress levels of participants as well as environmental, interpersonal and university-specific factors. Therefore, future research on the role of contributing factors for physical complaints should control for factors such as subjectively perceived levels of stress, environmental stressors (eg, examinations, housing situation) or interpersonal stressors (eg, relationship with classmates). Additional psychological factors (eg, catastrophising or negative affectivity) should also be considered in future research, as they have been shown to be associated with the development of medically unexplained symptoms.^16 39^ Furthermore, a more representative sample of students needs to be included in future research, as most participants in the present study studied psychology (79.8%), hence leading to a very specific population that is used to participating in studies and possesses at least basic psychological knowledge. While participants were not informed of specific hypotheses and objectives, it is still possible that their responses were influenced by existing knowledge. Additionally, methodological improvements should be considered. This includes increasing the number of assessment points per day and using randomised time points to clarify whether the effects found are independent of temporal fluctuations or possible time effects. Given the high compliance in the present sample and the application possibilities that the EMA approach provides, future studies could adopt more frequent, randomised assessments to address time-specific effects more precisely. Furthermore, the sequentially and semantically consistent presentation of 15 items measuring somatic symptom severity and symptom-related expectations at three time points per day may have influenced participants’ response behaviour due to a question order bias, potentially inflating the strong concurrent associations found in MLM 1. These associations might thus party reflect the similarity and contiguity of items. To control for this potential bias, future research should randomise the item order. Considering the potential biases mentioned for MLM 1, the effects found in MLM 2 might provide a more accurate representation of the associations between symptom-related expectations and subsequent somatic symptom severity. Moreover, future research should place a greater emphasis on investigating subgroups reporting very high or very low levels of symptom severity. Such analyses would be helpful to clarify whether the impact of symptom-related expectations or expectation framing on symptom severity might be different for groups with high levels of symptom severity compared with groups with very low levels of symptom severity. For a more comprehensive examination of how short-term developments and changes over time might be associated between symptom-related expectations and symptom severity (eg, a possible accumulation of effects over time), future research should focus on analysing growth trajectories within a structural equation modelling approach on a larger sample (eg, latent growth curve modelling).

## Conclusion

Altogether, the present study shed light onto the time-dynamic nature of somatic symptom severity in a sample of university students and supported EMA as a viable approach for identifying possible effects of expectations and expectation framing over a short period of time. The advantages of EMA (eg, prompt data collection in the participants environment) were an asset in this study. In order to gain insights into the course of somatic symptom and symptom expectations, we thus deem this approach appropriate for future research in the domain of somatic symptoms. To our knowledge, it is the first EMA-based study focusing on the roles of symptom-related expectations, self-management experiences and expectation framing for somatic symptoms of university students. The present findings support the assumptions that (1) somatic symptoms may be negatively or positively affected by expectations, (2) possible effects of expectations can be modulated through expectation framing and that (3) expectation management might therefore be a promising approach for reducing the perceived severity of short-term physical complaints in healthy individuals. However, the role of previously experienced somatic symptoms and self-management experiences for future somatic symptoms warrants further investigation. The development and implementation of micro-interventions for optimising expectations could improve coping with somatic symptoms in academic settings, thereby reducing stress and the risk of developing long-term somatic complaints .^57^ Micro-interventions such as the nocebo explanation (ie, providing written or verbal information about expectation/nocebo effects)^20 23 58^ are easily applicable in everyday life without significant time or financial investments. Future research should therefore focus on the effectiveness of interventions focusing on expectation framing both in healthy samples and clinical settings and clarify whether the associations found in the present study are generalisable to clinical populations such as patients with SSD.

## supplementary material

10.1136/bmjopen-2024-091032 online supplemental file 1

## Data Availability

Data are available upon reasonable request.
