# Supplementary material for: Time-dynamic associations between symptom-related expectations, self-management experiences and somatic symptom severity in everyday life: an ecological momentary assessment study with university students
Source: BMJ Open. 2025 Feb 7;15(2):e091032. doi: 10.1136/bmjopen-2024-091032 (PMC11808919; doi:10.1136/bmjopen-2024-091032)
Supplement: online supplemental file 1 [file bmjopen-15-2-s001.docx]

**Table a.** Descriptive statistics for the measures of symptom severity and symptom-related expectations for the total sample and both expectation framing groups.

|  | | **Total (*N* = 104)** | | | |  | **Expectation framing group** | | | | | | |  | **Test statistics for group comparisons** | | |
| --- | --- | --- | --- | --- | --- | --- | --- | --- | --- | --- | --- | --- | --- | --- | --- | --- | --- |
|  |  |  |  |  |  |  | **Negative (*n* = 52)** | | |  | **Positive (*n* = 52)** | | |  |  |  |  |
| **Variable** | **AP** | ***M*** | ***SD*** | **Min/Max** | **α** |  | ***M*** | ***SD*** | **α** |  | ***M*** | ***SD*** | **α** |  | ***t*** | ***df*** | ***p*** |
| PHQ_adapt_ [0-150] | 1 | 11.18 | 11.36 | 0/47 | 0.762 |  | 12.43 | 12.21 | 0.782 |  | 9.76 | 10.27 | 0.737 |  | 1.101 | 86 | 0.274 |
|  | 2 | 9.02 | 10.57 | 0/40 | 0.804 |  | 8.78 | 10.00 | 0.806 |  | 9.27 | 11.22 | 0.805 |  | -0.218 | 88 | 0.828 |
|  | 3 | 8.18 | 9.41 | 0/44 | 0.779 |  | 8.02 | 8.00 | 0.785 |  | 8.35 | 10.72 | 0.780 |  | -0.165 | 90 | 0.869 |
|  | 4 | 8.52 | 8.95 | 0/41 | 0.789 |  | 8.91 | 8.59 | 0.783 |  | 8.13 | 9.38 | 0.763 |  | 0.410 | 88 | 0.683 |
|  | 5 | 7.29 | 8.27 | 0/41 | 0.778 |  | 6.26 | 6.89 | 0.791 |  | 8.31 | 9.40 | 0.735 |  | -1.215 | 93 | 0.228 |
|  | 6 | 6.59 | 8.10 | 0/35 | 0.766 |  | 6.55 | 8.09 | 0.774 |  | 6.62 | 8.20 | 0.733 |  | -0.036 | 76 | 0.971 |
|  | 7 | 6.99 | 8.22 | 0/37 | 0.735 |  | 7.29 | 8.30 | 0.752 |  | 6.69 | 8.22 | 0.771 |  | 0.332 | 81 | 0.741 |
|  | 8 | 5.80 | 6.83 | 0/25 | 0.760 |  | 5.55 | 6.56 | 0.766 |  | 6.04 | 7.16 | 0.731 |  | -0.344 | 91 | 0.731 |
|  | 9 | 6.13 | 6.66 | 0/25 | 0.729 |  | 6.17 | 6.61 | 0.728 |  | 6.09 | 6.79 | 0.747 |  | 0.058 | 92 | 0.954 |
|  | 10 | 6.46 | 8.35 | 0/41 | 0.734 |  | 5.82 | 6.36 | 0.722 |  | 7.07 | 9.96 | 0.726 |  | -0.670 | 79 | 0.505 |
|  | 11 | 6.76 | 8.36 | 0/44 | 0.749 |  | 6.04 | 8.07 | 0.759 |  | 7.47 | 8.67 | 0.700 |  | -0.825 | 92 | 0.412 |
|  | 12 | 5.86 | 8.11 | 0/41 | 0.743 |  | 5.81 | 8.11 | 0.710 |  | 5.91 | 8.20 | 0.813 |  | -0.059 | 86 | 0.953 |
|  | 13 | 6.23 | 7.14 | 0/31 | 0.752 |  | 7.43 | 7.28 | 0.759 |  | 5.00 | 6.87 | 0.747 |  | 1.563 | 81 | 0.122 |
|  | 14 | 5.77 | 8.46 | 0/47 | 0.771 |  | 5.16 | 5.78 | 0.741 |  | 6.38 | 10.51 | 0.821 |  | -0.684 | 88 | 0.496 |
|  | 15 | 6.66 | 8.18 | 0/35 | 0.729 |  | 5.89 | 6.63 | 0.723 |  | 7.43 | 9.50 | 0.793 |  | -0.885 | 86 | 0.379 |
|  | 16 | 5.76 | 7.59 | 0/31 | 0.710 |  | 6.37 | 8.20 | 0.714 |  | 5.17 | 7.01 | 0.701 |  | 0.718 | 80 | 0.475 |
|  | 17 | 6.36 | 8.53 | 0/38 | 0.749 |  | 6.44 | 7.64 | 0.728 |  | 6.28 | 9.43 | 0.811 |  | 0.088 | 84 | 0.930 |
|  | 18 | 5.46 | 7.70 | 0/45 | 0.789 |  | 5.38 | 6.61 | 0.785 |  | 5.55 | 8.75 | 0.795 |  | -0.099 | 82 | 0.922 |
|  | 19 | 6.85 | 8.67 | 0/40 | 0.778 |  | 7.10 | 8.31 | 0.791 |  | 6.60 | 9.11 | 0.757 |  | 0.256 | 78 | 0.798 |
|  | 20 | 6.21 | 8.35 | 0/39 | 0.781 |  | 6.29 | 7.67 | 0.785 |  | 6.13 | 9.15 | 0.779 |  | 0.090 | 83 | 0.929 |
|  | 21 | 6.09 | 9.09 | 0/40 | 0.767 |  | 6.55 | 8.46 | 0.740 |  | 5.63 | 9.76 | 0.799 |  | 0.469 | 85 | 0.640 |
| NRS_expect_ [0-150] | 1 | 9.97 | 9.09 | 0/37 | 0.852 |  | 9.53 | 9.79 | 0.735 |  | 10.46 | 10.14 | 0.729 |  | -0.438 | 86 | 0.663 |
|  | 2 | 9.59 | 10.61 | 0/47 | 0.893 |  | 8.60 | 9.34 | 0.766 |  | 10.58 | 11.76 | 0.798 |  | -0.883 | 88 | 0.380 |
|  | 3 | 8.47 | 9.42 | 0/46 | 0.894 |  | 7.41 | 7.23 | 0.788 |  | 9.50 | 11.18 | 0.773 |  | -1.063 | 76.98 | 0.291 |
|  | 4 | 7.73 | 8.96 | 0/42 | 0.892 |  | 6.96 | 8.04 | 0.734 |  | 8.51 | 9.83 | 0.749 |  | -0.822 | 88 | 0.414 |
|  | 5 | 7.85 | 8.83 | 0/42 | 0.884 |  | 6.30 | 7.23 | 0.758 |  | 9.38 | 10.01 | 0.768 |  | -1.721 | 85.61 | 0.089 |
|  | 6 | 6.77 | 8.08 | 0/31 | 0.875 |  | 5.90 | 7.00 | 0.825 |  | 7.34 | 8.75 | 0.788 |  | -0.766 | 76 | 0.446 |
|  | 7 | 6.86 | 7.47 | 0/32 | 0.885 |  | 7.07 | 7.57 | 0.787 |  | 6.64 | 7.46 | 0.727 |  | 0.261 | 81 | 0.795 |
|  | 8 | 5.87 | 7.43 | 0/28 | 0.905 |  | 5.13 | 6.55 | 0.841 |  | 6.63 | 8.24 | 0.820 |  | -0.975 | 91 | 0.332 |
|  | 9 | 6.48 | 7.13 | 0/29 | 0.894 |  | 6.08 | 6.82 | 0.778 |  | 6.89 | 7.49 | 0.787 |  | -0.547 | 92 | 0.586 |
|  | 10 | 6.58 | 8.47 | 0/37 | 0.855 |  | 5.73 | 6.77 | 0.736 |  | 7.41 | 9.87 | 0.770 |  | -0.896 | 79 | 0.373 |
|  | 11 | 6.86 | 8.41 | 0/44 | 0.895 |  | 5.96 | 8.29 | 0.786 |  | 7.77 | 8.52 | 0.798 |  | -1.043 | 92 | 0.300 |
|  | 12 | 5.75 | 7.92 | 0/38 | 0.896 |  | 5.00 | 6.35 | 0.722 |  | 6.43 | 9.14 | 0.818 |  | -0.848 | 86 | 0.399 |
|  | 13 | 6.31 | 6.99 | 0/30 | 0.885 |  | 7.12 | 7.38 | 0.794 |  | 5.49 | 6.57 | 0.792 |  | 1.063 | 81 | 0.291 |
|  | 14 | 5.49 | 7.75 | 0/37 | 0.846 |  | 4.73 | 5.26 | 0.766 |  | 6.24 | 9.64 | 0.804 |  | -0.923 | 68.09 | 0.359 |
|  | 15 | 6.45 | 8.87 | 0/38 | 0.894 |  | 5.48 | 7.19 | 0.779 |  | 7.43 | 10.27 | 0.810 |  | -1.034 | 86 | 0.304 |
|  | 16 | 5.41 | 6.21 | 0/25 | 0.854 |  | 5.53 | 6.02 | 0.801 |  | 5.31 | 6.45 | 0.802 |  | 0.156 | 80 | 0.876 |
|  | 17 | 6.13 | 7.81 | 0/36 | 0.835 |  | 6.16 | 7.38 | 0.735 |  | 6.09 | 8.32 | 0.767 |  | 0.041 | 84 | 0.967 |
|  | 18 | 6.06 | 8.42 | 0/42 | 0.863 |  | 6.10 | 8.06 | 0.748 |  | 6.02 | 8.88 | 0.765 |  | 0.039 | 82 | 0.969 |
|  | 19 | 6.35 | 8.22 | 0/34 | 0.925 |  | 6.05 | 7.19 | 0.761 |  | 6.65 | 9.22 | 0.837 |  | -0.325 | 78 | 0.746 |
|  | 20 | 6.07 | 7.76 | 0/34 | 0.902 |  | 6.22 | 7.35 | 0.812 |  | 5.90 | 8.29 | 0.746 |  | 0.190 | 83 | 0.850 |
|  | 21 | 6.23 | 9.22 | 0/39 | 0.894 |  | 6.32 | 9.11 | 0.740 |  | 6.14 | 9.45 | 0.769 |  | 0.090 | 85 | 0.929 |


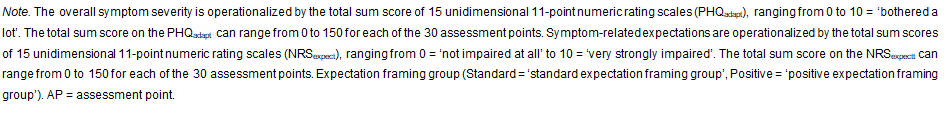


**Table b.** Sociodemographic and healthcare-related characteristics of the total sample and both expectation framing groups.

|  |  |  | | | |  | Expectation framing group | | | | | | |  | Test statistics for group comparisons | | | | | | |
| --- | --- | --- | --- | --- | --- | --- | --- | --- | --- | --- | --- | --- | --- | --- | --- | --- | --- | --- | --- | --- | --- |
|  |  | Total sample (*N* = 104) | | | |  | Negative (*n* = 52) | | |  | Positive (*n* = 52) | | |  | Homogeneity | | |  | Difference | | |
| Variable |  | *n* (%) | *M* | *SD* | Min/Max |  | *n* (%) | *M* | *SD* |  | *n* (%) | *M* | *SD* |  | χ^2^ | *df* | *p_asym_* |  | *t* | *df* | *p* |
| Gender |  | 104 (100) |  |  | 1 / 2 |  | 52 (100) |  |  |  | 52 (100) |  |  |  | 0.000 | 1 | 1.000 |  |  |  |  |
| male (1) |  | 66 (63.5) |  |  |  |  | 33 (63.5) |  |  |  | 33 (63.5) |  |  |  |  |  |  |  |  |  |  |
| Age (in years) |  | 104 (100) | 24.27 | 2.70 | 19 / 33 |  | 52 (100) | 23.96 | 2.66 |  | 52 (100) | 24.58 | 2.73 |  |  |  |  |  | 1.170 | 102 | 0.475 |
| Educational level |  | 104 (100) |  |  | 1 / 2 |  | 52 (100) |  |  |  | 52 (100) |  |  |  | 1.480 | 1 | 0.477 |  |  |  |  |
| higher (1) |  | 50 (48.1) |  |  |  |  | 27 (51.9) |  |  |  | 23 (44.2) |  |  |  |  |  |  |  |  |  |  |
| Migration background |  | 104 (100) |  |  | 0 / 1 |  | 52 (100) |  |  |  | 52 (100) |  |  |  | 1.182 | 1 | 0.277 |  |  |  |  |
| with (1) |  | 16 (15.4) |  |  |  |  | 6  (11.5) |  |  |  | 10 (19.2) |  |  |  |  |  |  |  |  |  |  |
| Partnership |  | 104 (100) |  |  | 0 / 1 |  | 52 (100) |  |  |  | 52 (100) |  |  |  | 0.699 | 1 | 0.403 |  |  |  |  |
| yes (1) |  | 70 (67.3) |  |  |  |  | 37 (71.2) |  |  |  | 33 (63.5) |  |  |  |  |  |  |  |  |  |  |
| Medical consultations (4w) |  | 51 (49.0) | 0.87 | 1.25 | 0 / 7 |  | 22 (42.3) | 0.71 | 1.24 |  | 29 (55.8) | 1.02 | 1.24 |  | 6.530 | 6 | 0.366 |  | -1.262 | 102 | 0.600 |
| Medical consultations (6m) |  | 90 (86.5) | 3.26 | 3.61 | 0 / 15 |  | 44 (84.6) | 2.71 | 3.41 |  | 46  (88.5) | 3.81 | 3.75 |  | 10.353 | 12 | 0.585 |  | -1.559 | 102 | 0.249 |
| Somatic Symptom |  | *n* (%) | *M* | *SD* | Sum (Min/Max) |  | *n* (%) | *M* | *SD* |  | *n* (%) | *M* | *SD* |  | χ^2^ | *df* | *p_asym_* |  | *t* | *df* | *p* |
| Stomach pain |  | 45 (43.3) | 1.71 | 3.25 | 178 (0/18) |  | 25 (48.1) | 1.77 | 2.819 |  | 20 (38.4) | 1.65 | 3.65 |  |  |  |  |  | 0.180 | 102 | 0.738 |
| Back pain |  | 71 (68.3) | 4.46 | 5.73 | 464 (0/21) |  | 35 (67.3) | 4.54 | 5.768 |  | 36 (69.2) | 4.38 | 5.75 |  |  |  |  |  | 0.136 | 102 | 0.560 |
| Pain in arms, legs or joints (Knees, hips, etc.) |  | 65 (62.5) | 4.27 | 5.69 | 444 (0/20) |  | 33 (63.4) | 4.40 | 5.730 |  | 32 (61.5) | 4.13 | 5.71 |  |  |  |  |  | 0.240 | 102 | 0.910 |
| Menstrual cramps or other problems with your period |  | 14 (13.5) | 0.36 | 1.32 | 37 (0/10) |  | 5 (9.6) | 0.23 | 1.022 |  | 9 (17.3) | 0.48 | 1.57 |  |  |  |  |  | -0.964 | 102 | 0.099 |
| Headaches |  | 82 (78.8) | 5.05 | 5.61 | 525 (0/20) |  | 41 (78.8) | 5.40 | 5.696 |  | 41 (78.8) | 4.69 | 5.56 |  |  |  |  |  | 0.645 | 102 | 0.793 |
| Chest pain |  | 30 (28.8) | 1.10 | 3.40 | 114 (0/21) |  | 13 (25.0) | 0.73 | 2.450 |  | 17 (32.7) | 1.46 | 4.12 |  |  |  |  |  | -1.099 | 102 | 0.076 |
| Dizziness |  | 44 (42.3) | 1.41 | 2.94 | 147 (0/21) |  | 21 (40.4) | 1.27 | 2.490 |  | 23 (44.2) | 1.56 | 3.35 |  |  |  |  |  | 0.310 | 102 | 0.452 |
| Fainting spells |  | 11 (10.6) | 0.17 | 0.76 | 18 (0/7) |  | 4 (7.7) | 0.19 | 0.991 |  | 7 (13.4) | 0.15 | 0.42 |  |  |  |  |  | 0.258 | 102 | 0.514 |
| Feeling heart pound or race |  | 40 (38.5) | 1.44 | 3.30 | 150 (0/21) |  | 15 (28.8) | 0.92 | 2.131 |  | 25 (48.1) | 1.96 | 4.12 |  |  |  |  |  | -1.616 | 102 | 0.044 |
| Shortness of breath |  | 34 (32.7) | 1.87 | 4.54 | 194 (0/21) |  | 16 (30.7) | 1.23 | 3.341 |  | 18 (34.6) | 2.50 | 5.44 |  |  |  |  |  | -1.432 | 102 | 0.006 |
| Pain or problems during sexual intercourse |  | 17 (16.3) | 0.53 | 1.87 | 55 (0/12) |  | 10 (19.2) | 0.58 | 1.913 |  | 7 (13.5) | 0.48 | 1.84 |  |  |  |  |  | 0.261 | 102 | 0.759 |
| Constipation, loose bowels, or diarrhea |  | 56 (53.8) | 2.50 | 4.29 | 228 (0/21) |  | 29 (55.7) | 2.50 | 4.291 |  | 27 (51.9) | 2.27 | 4.37 |  |  |  |  |  | 0.272 | 102 | 0.721 |
| Nausea, gas, or indigestion |  | 64 (61.5) | 2.90 | 4.29 | 299 (0/21) |  | 35 (67.3) | 2.90 | 4.294 |  | 29 (55.7) | 2.85 | 4.57 |  |  |  |  |  | 0.066 | 102 | 0.902 |
| Feeling tired or having low energy |  | 91 (87.5) | 8.79 | 7.21 | 922 (0/21) |  | 47 (90.1) | 8.79 | 7.212 |  | 44 (84.6) | 8.94 | 6.93 |  |  |  |  |  | -0.111 | 102 | 0.337 |
| Trouble sleeping |  | 59 (56.7) | 3.37 | 5.10 | 336 (0/21) |  | 31 (59.6) | 3.37 | 5.228 |  | 28  (53.8) | 3.10 | 5.01 |  |  |  |  |  | 0.268 | 102 | 0.857 |
| Sum of somatic complaints |  | 103 (99.1) | 39.72 | 38.13 | 4131 (0/215) |  | 52 (100) | 38.83 | 32.89 |  | 51 (98.1) | 40.62 | 43.05 |  |  |  |  |  | -0.238 | 102 | 0.447 |

*Note.* Educational level is defined as the highest level of education achieved by the participant (higher (1) = ‘working towards a bachelor's degree’, highest (2) = ‘working towards a master’s degree’). Migration background is indicated when either the participant or at least one parent was not born in Germany. For the sake of readability, descriptive statistics for only one category are depicted for the variables Gender, Educational Background, Migration Background, and Partnership. Test statistics for group comparisons are based on all categories of the respective variables. Medical consultations (4w) = Total number of medical consultations that had been attended within the last four weeks. Medical consultations (6m) = Total number of medical consultations that had been attended within the last six months. Somatic symptoms are operationalized by the total sum score of 15 unidimensional 11-point numeric rating scales (PHQ_adapt_ ranging from 0 = ‘not impaired at all’ to 10 = ‘very strongly impaired’). Scores were coded as binary (0 = no somatic complaints, 1 = somatic complaints) for the frequencies of somatic symptoms shown. *N* refers to the total number of reported somatic complaints for the specific symptom over a 7-day period. *Min/Max* describes the days on which the least/most somatic complaints were reported.

**Sensitivity Analysis**

|  |  | **MLM 1: concurrent associations (sensitivity analysis)** | | | | | |
| --- | --- | --- | --- | --- | --- | --- | --- |
| **Variable** |  | **β** | ***B*** | ***SE (B)*** | ***t*** | ***p*** | **95% CI (*B*)** |
| Symptom-related expectations |  | 0.908 | 0.936 | 0.019 | 49.792 | < 0.001 | [0.899. 0.974] |
| Expectation framing |  | -0.113 | -1.604 | 0.254 | -6.302 | < 0.001 | [-2.113. -1.094] |
| Expectation × Framing |  | -0.116 | -0.233 | 0.040 | -5.887 | < 0.001 | [-0.312. -0.154] |
| Self-management experiences |  | 0.014 | 0.210 | 0.128 | 1.634 | 0.107 | [-0.047. 0.467] |

**Table c.** Sensitivity analysis for multilevel mixed-effects linear regression analysis for the associations with concurrent levels of somatic symptom severity as the criterion variable

*Note.* N = 6 outliers were excluded for the sensitivity analysis. Symptom-related expectations are operationalized by the total sum score of 15 unidimensional 11-point numeric rating scales (NRS_expect_ ranging from 0 = ‘not impairment free at all’ to 10 = ‘highly impairment free’). Expectation framing = expectations framing group (1 = ‘negative expectation framing group’, 2 = ‘positive expectation framing group’). Expectation × Framing = cross-product term for the interaction between the predictor variables symptom-related expectations and expectation framing. Self-management experiences are defined as the total number of individual self-management interventions of short-term somatic symptoms per assessment point and measured by the total sum score of 15 binary rating items (NRS_treat_ with 0 = ‘no’, 1 = ‘yes’). Somatic symptom severity (criterion variable) is operationalized by the total sum score of 15 unidimensional 11-point numeric rating scales (PHQ_adapt_ ranging from 0 = ‘not bothered at all’ to 10 = ‘bothered a lot’).

**Table d.** Sensitivity analysis for multilevel mixed-effects linear regression analysis for the associations with concurrent levels of somatic symptom severity as the criterion variable

*Note.* N = 6 outliers were excluded for the sensitivity analysis. Symptom-related expectations are operationalized by the total sum score of 15 unidimensional 11-point numeric rating scales (NRS_expect_ ranging from 0 = ‘not impairment free at all’ to 10 = ‘highly impairment free’). Expectation framing = expectations framing group (1 = ‘negative expectation framing group’, 2 = ‘positive expectation framing group’). Expectation × Framing = cross-product term for the interaction between the predictor variables symptom-related expectations and expectation framing. Self-management experiences are defined as the total number of individual self-management interventions of short-term somatic symptoms per assessment point and measured by the total sum score of 15 binary rating items (NRS_treat_ with 0 = ‘no’, 1 = ‘yes’). Somatic symptom severity (criterion variable) is operationalized by the total sum score of 15 unidimensional 11-point numeric rating scales (PHQ_adapt_ ranging from 0 = ‘not bothered at all’ to 10 = ‘bothered a lot’).

|  |  | **MLM 2: time-lagged associations (sensitivity analysis)** | | | | | |
| --- | --- | --- | --- | --- | --- | --- | --- |
| **Variable** |  | **β** | ***B*** | ***SE (B)*** | ***t*** | ***p*** | **95% CI (*B*)** |
| Symptom-related expectations |  | 0.233 | 0.236 | 0.064 | 3.683 | <0.001 | [0.110. 0.362] |
| Expectation framing |  | -0.146 | -2.073 | 0.893 | -2.321 | 0.020 | [-3.825. -0.321] |
| Expectation × Framing |  | -0.061 | -0.122 | 0.066 | -1.851 | 0.064 | [-0.251. 0.007] |
| Self-management experiences |  | -0.011 | -0.169 | 0.352 | -0.481 | 0.631 | [-0.859. 0.521] |
| Somatic symptom severity (prior) |  | 0.033 | 0.032 | 0.063 | 0.506 | 0.613 | [-0.091. 0.155] |

**Adapted Questionnaire (PHQ_adapt_): Negative Expectation Framing. Morning Assessment**

**Online-Befragung am Morgen** (8:00 Uhr bis 10:00 Uhr)

**Einleitung:** *„Herzlich willkommen zur Online-Befragung der* *SymTrack 2.0 Pilotstudie. Vielen Dank. dass Sie sich heute Morgen Zeit dafür nehmen.“*

| Im folgenden Abschnitt geht es darum. einzuschätzen. durch welche körperlichen Beschwerden Sie seit dem letzten Abend beeinträchtigt sind. welche Erwartungen Sie bezüglich des Verlaufs der Beschwerden haben und ob Sie bereits etwas gegen die Beschwerden unternommen haben: | | | | | | | | | | | | | | |
| --- | --- | --- | --- | --- | --- | --- | --- | --- | --- | --- | --- | --- | --- | --- |
| **Wie stark fühlen Sie sich seit dem letzten Abend durch die folgenden Beschwerden beeinträchtigt?** | | | | | | | | | | | | | | |
| **1. Bauchschmerzen** | | | | | | | | | | | | | | PHQ_adapt_  Item 1 |
| **nicht beeinträchtigt** | 0 | 1 | 2 | 3 | 4 | 5 | | 6 | 7 | 8 | 9 | 10 | **sehr stark**  **beeinträchtigt** | |
| 1.2 Wie sehr erwarten Sie bis zum Mittag durch die *Bauchschmerzen* beeinträchtigt zu sein? | | | | | | | | | | | | | | NRS_expect_  Item 1 |
| **nicht beeinträchtigt** | 0 | 1 | 2 | 3 | 4 | 5 | | 6 | 7 | 8 | 9 | 10 | **sehr stark**  **beeinträchtigt** | |
| 1.3 Haben Sie seit dem letzten Abend etwas unternommen. um die *Bauchschmerzen* zu behandeln? | | | | | | | | | | | | | | NRS_treat1_  Item 1 |
| Ja | | | | | | | Nein | | | | | | | |
| **2. Rückenschmerzen** | | | | | | | | | | | | | | PHQ_adapt_  Item 2 |
| **nicht beeinträchtigt** | 0 | 1 | 2 | 3 | 4 | 5 | | 6 | 7 | 8 | 9 | 10 | **sehr stark**  **beeinträchtigt** | |
| 2.2 Wie sehr erwarten Sie bis zum Mittag durch die *Rückenschmerzen* beeinträchtigt zu sein? | | | | | | | | | | | | | | NRS_expect_  Item 2 |
| **nicht beeinträchtigt** | 0 | 1 | 2 | 3 | 4 | 5 | | 6 | 7 | 8 | 9 | 10 | **sehr stark**  **beeinträchtigt** | |
| 2.3 Haben Sie seit dem letzten Abend etwas unternommen. um die *Rückenschmerzen* zu behandeln? | | | | | | | | | | | | | | NRS_treat1_  Item 2 |
| Ja | | | | | | | Nein | | | | | | | |
| 2.4 Wie sehr hat die Behandlung Ihre Beeinträchtigung durch die *Rückenschmerzen* verbessert? | | | | | | | | | | | | | | NRS_treat2_  Item 2 |
| **keine**  **Verbesserung** | 0 | 1 | 2 | 3 | 4 | 5 | | 6 | 7 | 8 | 9 | 10 | **sehr starke Verbesserung** | |
| 2.5 Wie sehr hat die Behandlung Ihre Beeinträchtigung durch die *Rückenschmerzen* verschlechtert? | | | | | | | | | | | | | | NRS_treat3_  Item 2 |
| **keine Verschlechterung** | 0 | 1 | 2 | 3 | 4 | 5 | | 6 | 7 | 8 | 9 | 10 | **sehr starke Verschlechterung** | |
| 2.6 Wie sehr haben Sie unangenehme Nebenwirkungen durch die Behandlung erlebt? | | | | | | | | | | | | | | TreatExp  Item 2 |
| **keine Nebenwirkungen** | 0 | 1 | 2 | 3 | 4 | 5 | | 6 | 7 | 8 | 9 | 10 | **sehr starke Nebenwirkungen** | |
| **3. Schmerzen in Armen. Beinen oder Gelenken (Knie. Hüften. usw.)** | | | | | | | | | | | | | | PHQ_adapt_  Item 3 |
| **nicht beeinträchtigt** | 0 | 1 | 2 | 3 | 4 | 5 | | 6 | 7 | 8 | 9 | 10 | **sehr stark**  **beeinträchtigt** | |
| 3.2 Wie sehr erwarten Sie bis zum Mittag durch die *Schmerzen in Armen. Beinen oder Gelenken (Knie. Hüften. usw.)* beeinträchtigt zu sein? | | | | | | | | | | | | | | NRS_expect_  Item 3 |
| **nicht beeinträchtigt** | 0 | 1 | 2 | 3 | 4 | 5 | | 6 | 7 | 8 | 9 | 10 | **sehr stark**  **beeinträchtigt** | |
| 3.3 Haben Sie seit dem letzten Abend etwas unternommen. um die *Schmerzen in Armen. Beinen oder Gelenken (Knie. Hüften. usw.)* zu behandeln? | | | | | | | | | | | | | | NRS_treat1_  Item 3 |
| Ja | | | | | | | Nein | | | | | | | |
| 3.4 Wie sehr hat die Behandlung Ihre Beeinträchtigung durch die *Schmerzen in Armen. Beinen oder Gelenken (Knie. Hüften. usw.)* verbessert? | | | | | | | | | | | | | | NRS_treat2_  Item 3 |
| **keine**  **Verbesserung** | 0 | 1 | 2 | 3 | 4 | 5 | | 6 | 7 | 8 | 9 | 10 | **sehr starke Verbesserung** | |
| 3.5 Wie sehr hat die Behandlung Ihre Beeinträchtigung durch die *Schmerzen in Armen. Beinen oder Gelenken (Knie. Hüften. usw.)* verschlechtert? | | | | | | | | | | | | | | NRS_treat3_  Item 3 |
| **keine Verschlechterung** | 0 | 1 | 2 | 3 | 4 | 5 | | 6 | 7 | 8 | 9 | 10 | **sehr starke Verschlechterung** | |
| 3.6 Wie sehr haben Sie unangenehme Nebenwirkungen durch die Behandlung erlebt? | | | | | | | | | | | | | | TreatExp  Item 3 |
| **keine Nebenwirkungen** | 0 | 1 | 2 | 3 | 4 | 5 | | 6 | 7 | 8 | 9 | 10 | **sehr starke Nebenwirkungen** | |
| **4. Menstruationsschmerzen oder andere Probleme mit der Menstruation** | | | | | | | | | | | | | | PHQ_adapt_  Item 4 |
| **nicht beeinträchtigt** | 0 | 1 | 2 | 3 | 4 | 5 | | 6 | 7 | 8 | 9 | 10 | **sehr stark**  **beeinträchtigt** | |
| 4.2 Wie sehr erwarten Sie bis zum Mittag durch die *Menstruationsschmerzen oder andere Probleme mit der Menstruation* beeinträchtigt zu sein? | | | | | | | | | | | | | | NRS_expect_  Item 4 |
| **nicht beeinträchtigt** | 0 | 1 | 2 | 3 | 4 | 5 | | 6 | 7 | 8 | 9 | 10 | **sehr stark**  **beeinträchtigt** | |
| 4.3 Haben Sie seit dem letzten Abend etwas unternommen. um die *Menstruationsschmerzen oder andere Probleme mit der Menstruation* zu behandeln? | | | | | | | | | | | | | | NRS_treat1_  Item 4 |
| Ja | | | | | | | Nein | | | | | | | |
| 4.4 Wie sehr hat die Behandlung Ihre Beeinträchtigung durch die *Menstruationsschmerzen oder andere Probleme mit der Menstruation* verbessert? | | | | | | | | | | | | | | NRS_treat2_  Item 4 |
| **keine**  **Verbesserung** | 0 | 1 | 2 | 3 | 4 | 5 | | 6 | 7 | 8 | 9 | 10 | **sehr starke Verbesserung** | |
| 4.5 Wie sehr hat die Behandlung Ihre Beeinträchtigung durch die *Menstruationsschmerzen oder andere Probleme mit der Menstruation* verschlechtert? | | | | | | | | | | | | | | NRS_treat3_  Item 4 |
| **keine Verschlechterung** | 0 | 1 | 2 | 3 | 4 | 5 | | 6 | 7 | 8 | 9 | 10 | **sehr starke Verschlechterung** | |
| 4.6 Wie sehr haben Sie unangenehme Nebenwirkungen durch die Behandlung erlebt? | | | | | | | | | | | | | | TreatExp  Item 4 |
| **keine Nebenwirkungen** | 0 | 1 | 2 | 3 | 4 | 5 | | 6 | 7 | 8 | 9 | 10 | **sehr starke Nebenwirkungen** | |
| **5. Schmerzen oder Probleme beim Geschlechtsverkehr** | | | | | | | | | | | | | | PHQ_adapt_  Item 5 |
| **nicht beeinträchtigt** | 0 | 1 | 2 | 3 | 4 | 5 | | 6 | 7 | 8 | 9 | 10 | **sehr stark**  **beeinträchtigt** | |
| 5.2 Wie sehr erwarten Sie bis zum Mittag durch die *Schmerzen oder Probleme beim Geschlechtsverkehr* beeinträchtigt zu sein? | | | | | | | | | | | | | | NRS_expect_  Item 5 |
| **nicht beeinträchtigt** | 0 | 1 | 2 | 3 | 4 | 5 | | 6 | 7 | 8 | 9 | 10 | **sehr stark**  **beeinträchtigt** | |
| 5.3 Haben Sie seit dem letzten Abend etwas unternommen. um die *Schmerzen oder Probleme beim Geschlechtsverkehr* zu behandeln? | | | | | | | | | | | | | | NRS_treat1_  Item 5 |
| Ja | | | | | | | Nein | | | | | | | |
| 5.4 Wie sehr hat die Behandlung Ihre Beeinträchtigung durch die *Schmerzen oder Probleme beim Geschlechtsverkehr* verbessert? | | | | | | | | | | | | | | NRS_treat2_  Item 5 |
| **keine**  **Verbesserung** | 0 | 1 | 2 | 3 | 4 | 5 | | 6 | 7 | 8 | 9 | 10 | **sehr starke Verbesserung** | |
| 5.5 Wie sehr hat die Behandlung Ihre Beeinträchtigung durch die *Schmerzen oder Probleme beim Geschlechtsverkehr* verschlechtert? | | | | | | | | | | | | | | NRS_treat3_  Item 5 |
| **keine Verschlechterung** | 0 | 1 | 2 | 3 | 4 | 5 | | 6 | 7 | 8 | 9 | 10 | **sehr starke Verschlechterung** | |
| 5.6 Wie sehr haben Sie unangenehme Nebenwirkungen durch die Behandlung erlebt? | | | | | | | | | | | | | | TreatExp  Item 5 |
| **keine Nebenwirkungen** | 0 | 1 | 2 | 3 | 4 | 5 | | 6 | 7 | 8 | 9 | 10 | **sehr starke Nebenwirkungen** | |
| **6. Kopfschmerzen** | | | | | | | | | | | | | | PHQ_adapt_  Item 6 |
| **nicht beeinträchtigt** | 0 | 1 | 2 | 3 | 4 | 5 | | 6 | 7 | 8 | 9 | 10 | **sehr stark**  **beeinträchtigt** | |
| 6.2 Wie sehr erwarten Sie bis zum Mittag durch die *Kopfschmerzen* beeinträchtigt zu sein? | | | | | | | | | | | | | | NRS_expect_  Item 6 |
| **nicht beeinträchtigt** | 0 | 1 | 2 | 3 | 4 | 5 | | 6 | 7 | 8 | 9 | 10 | **sehr stark**  **beeinträchtigt** | |
| 6.3 Haben Sie seit dem letzten Abend etwas unternommen. um die *Kopfschmerzen* zu behandeln? | | | | | | | | | | | | | | NRS_treat1_  Item 6 |
| Ja | | | | | | | Nein | | | | | | | |
| 6.4 Wie sehr hat die Behandlung Ihre Beeinträchtigung durch die *Kopfschmerzen* verbessert? | | | | | | | | | | | | | | NRS_treat2_  Item 6 |
| **keine**  **Verbesserung** | 0 | 1 | 2 | 3 | 4 | 5 | | 6 | 7 | 8 | 9 | 10 | **sehr starke Verbesserung** | |
| 6.5 Wie sehr hat die Behandlung Ihre Beeinträchtigung durch die *Kopfschmerzen* verschlechtert? | | | | | | | | | | | | | | NRS_treat3_  Item 6 |
| **keine Verschlechterung** | 0 | 1 | 2 | 3 | 4 | 5 | | 6 | 7 | 8 | 9 | 10 | **sehr starke Verschlechterung** | |
| 6.6 Wie sehr haben Sie unangenehme Nebenwirkungen durch die Behandlung erlebt? | | | | | | | | | | | | | | TreatExp  Item 6 |
| **keine Nebenwirkungen** | 0 | 1 | 2 | 3 | 4 | 5 | | 6 | 7 | 8 | 9 | 10 | **sehr starke Nebenwirkungen** | |
| **7. Schmerzen im Brustbereich** | | | | | | | | | | | | | | PHQ_adapt_  Item 7 |
| **nicht beeinträchtigt** | 0 | 1 | 2 | 3 | 4 | 5 | | 6 | 7 | 8 | 9 | 10 | **sehr stark**  **beeinträchtigt** | |
| 7.2 Wie sehr erwarten Sie bis zum Mittag durch die *Schmerzen im Brustbereich* beeinträchtigt zu sein? | | | | | | | | | | | | | | NRS_expect_  Item 7 |
| **nicht beeinträchtigt** | 0 | 1 | 2 | 3 | 4 | 5 | | 6 | 7 | 8 | 9 | 10 | **sehr stark**  **beeinträchtigt** | |
| 7.3 Haben Sie seit dem letzten Abend etwas unternommen. um die *Schmerzen im Brustbereich* zu behandeln? | | | | | | | | | | | | | | NRS_treat1_  Item 7 |
| Ja | | | | | | | Nein | | | | | | | |
| 7.4 Wie sehr hat die Behandlung Ihre Beeinträchtigung durch die *Schmerzen im Brustbereich* verbessert? | | | | | | | | | | | | | | NRS_treat2_  Item 7 |
| **keine**  **Verbesserung** | 0 | 1 | 2 | 3 | 4 | 5 | | 6 | 7 | 8 | 9 | 10 | **sehr starke Verbesserung** | |
| 7.5 Wie sehr hat die Behandlung Ihre Beeinträchtigung durch die *Schmerzen im Brustbereich* verschlechtert? | | | | | | | | | | | | | | NRS_treat3_  Item 7 |
| **keine Verschlechterung** | 0 | 1 | 2 | 3 | 4 | 5 | | 6 | 7 | 8 | 9 | 10 | **sehr starke Verschlechterung** | |
| 7.6 Wie sehr haben Sie unangenehme Nebenwirkungen durch die Behandlung erlebt? | | | | | | | | | | | | | | TreatExp  Item 7 |
| **keine Nebenwirkungen** | 0 | 1 | 2 | 3 | 4 | 5 | | 6 | 7 | 8 | 9 | 10 | **sehr starke Nebenwirkungen** | |
| **8. Schwindel** | | | | | | | | | | | | | | PHQ_adapt_  Item 8 |
| **nicht beeinträchtigt** | 0 | 1 | 2 | 3 | 4 | 5 | | 6 | 7 | 8 | 9 | 10 | **sehr stark**  **beeinträchtigt** | |
| 8.2 Wie sehr erwarten Sie bis zum Mittag durch den *Schwindel* beeinträchtigt zu sein? | | | | | | | | | | | | | | NRS_expect_  Item 8 |
| **nicht beeinträchtigt** | 0 | 1 | 2 | 3 | 4 | 5 | | 6 | 7 | 8 | 9 | 10 | **sehr stark**  **beeinträchtigt** | |
| 8.3 Haben Sie seit dem letzten Abend etwas unternommen. um den *Schwindel* zu behandeln? | | | | | | | | | | | | | | NRS_treat1_  Item 8 |
| Ja | | | | | | | Nein | | | | | | | |
| 8.4 Wie sehr hat die Behandlung Ihre Beeinträchtigung durch den *Schwindel* verbessert? | | | | | | | | | | | | | | NRS_treat2_  Item 8 |
| **keine**  **Verbesserung** | 0 | 1 | 2 | 3 | 4 | 5 | | 6 | 7 | 8 | 9 | 10 | **sehr starke Verbesserung** | |
| 8.5 Wie sehr hat die Behandlung Ihre Beeinträchtigung durch den *Schwindel* verschlechtert? | | | | | | | | | | | | | | NRS_treat3_  Item 8 |
| **keine Verschlechterung** | 0 | 1 | 2 | 3 | 4 | 5 | | 6 | 7 | 8 | 9 | 10 | **sehr starke Verschlechterung** | |
| 8.6 Wie sehr haben Sie unangenehme Nebenwirkungen durch die Behandlung erlebt? | | | | | | | | | | | | | | TreatExp  Item 8 |
| **keine Nebenwirkungen** | 0 | 1 | 2 | 3 | 4 | 5 | | 6 | 7 | 8 | 9 | 10 | **sehr starke Nebenwirkungen** | |
| **9. Ohnmachtsanfälle** | | | | | | | | | | | | | | PHQ_adapt_  Item 9 |
| **nicht beeinträchtigt** | 0 | 1 | 2 | 3 | 4 | 5 | | 6 | 7 | 8 | 9 | 10 | **sehr stark**  **beeinträchtigt** | |
| 9.2 Wie sehr erwarten Sie bis zum Mittag durch die *Ohnmachtsanfälle* beeinträchtigt zu sein? | | | | | | | | | | | | | | NRS_expect_  Item 9 |
| **nicht beeinträchtigt** | 0 | 1 | 2 | 3 | 4 | 5 | | 6 | 7 | 8 | 9 | 10 | **sehr stark**  **beeinträchtigt** | |
| 9.3 Haben Sie seit dem letzten Abend etwas unternommen. um die *Ohnmachtsanfälle* zu behandeln? | | | | | | | | | | | | | | NRS_treat1_  Item 9 |
| Ja | | | | | | | Nein | | | | | | | |
| 9.4 Wie sehr hat die Behandlung Ihre Beeinträchtigung durch die *Ohnmachtsanfälle* verbessert? | | | | | | | | | | | | | | NRS_treat2_  Item 9 |
| **keine**  **Verbesserung** | 0 | 1 | 2 | 3 | 4 | 5 | | 6 | 7 | 8 | 9 | 10 | **sehr starke Verbesserung** | |
| 9.5 Wie sehr hat die Behandlung Ihre Beeinträchtigung durch die *Ohnmachtsanfälle* verschlechtert? | | | | | | | | | | | | | | NRS_treat3_  Item 9 |
| **keine Verschlechterung** | 0 | 1 | 2 | 3 | 4 | 5 | | 6 | 7 | 8 | 9 | 10 | **sehr starke Verschlechterung** | |
| 9.6 Wie sehr haben Sie unangenehme Nebenwirkungen durch die Behandlung erlebt? | | | | | | | | | | | | | | TreatExp  Item 9 |
| **keine Nebenwirkungen** | 0 | 1 | 2 | 3 | 4 | 5 | | 6 | 7 | 8 | 9 | 10 | **sehr starke Nebenwirkungen** | |
| **10. Herzklopfen oder Herzrasen** | | | | | | | | | | | | | | PHQ_adapt_  Item 10 |
| **nicht beeinträchtigt** | 0 | 1 | 2 | 3 | 4 | 5 | | 6 | 7 | 8 | 9 | 10 | **sehr stark**  **beeinträchtigt** | |
| 10.2 Wie sehr erwarten Sie bis zum Mittag durch das *Herzklopfen oder Herzrasen* beeinträchtigt zu sein? | | | | | | | | | | | | | | NRS_expect_  Item 10 |
| **nicht beeinträchtigt** | 0 | 1 | 2 | 3 | 4 | 5 | | 6 | 7 | 8 | 9 | 10 | **sehr stark**  **beeinträchtigt** | |
| 10.3 Haben Sie seit dem letzten Abend etwas unternommen. um das *Herzklopfen oder Herzrasen* zu behandeln? | | | | | | | | | | | | | | NRS_treat1_  Item 10 |
| Ja | | | | | | | Nein | | | | | | | |
| 10.4 Wie sehr hat die Behandlung Ihre Beeinträchtigung durch das *Herzklopfen oder Herzrasen* verbessert? | | | | | | | | | | | | | | NRS_treat2_  Item 10 |
| **keine**  **Verbesserung** | 0 | 1 | 2 | 3 | 4 | 5 | | 6 | 7 | 8 | 9 | 10 | **sehr starke Verbesserung** | |
| 10.5 Wie sehr hat die Behandlung Ihre Beeinträchtigung durch das *Herzklopfen oder Herzrasen* verschlechtert? | | | | | | | | | | | | | | NRS_treat3_  Item 10 |
| **keine Verschlechterung** | 0 | 1 | 2 | 3 | 4 | 5 | | 6 | 7 | 8 | 9 | 10 | **sehr starke Verschlechterung** | |
| 10.6 Wie sehr haben Sie unangenehme Nebenwirkungen durch die Behandlung erlebt? | | | | | | | | | | | | | | TreatExp  Item 10 |
| **keine Nebenwirkungen** | 0 | 1 | 2 | 3 | 4 | 5 | | 6 | 7 | 8 | 9 | 10 | **sehr starke Nebenwirkungen** | |
| **11. Kurzatmigkeit** | | | | | | | | | | | | | | PHQ_adapt_  Item 11 |
| **nicht beeinträchtigt** | 0 | 1 | 2 | 3 | 4 | 5 | | 6 | 7 | 8 | 9 | 10 | **sehr stark**  **beeinträchtigt** | |
| 11.2 Wie sehr erwarten Sie bis zum Mittag durch die *Kurzatmigkeit* beeinträchtigt zu sein? | | | | | | | | | | | | | | NRS_expect_  Item 11 |
| **nicht beeinträchtigt** | 0 | 1 | 2 | 3 | 4 | 5 | | 6 | 7 | 8 | 9 | 10 | **sehr stark**  **beeinträchtigt** | |
| 11.3 Haben Sie seit dem letzten Abend etwas unternommen. um die *Kurzatmigkeit* zu behandeln? | | | | | | | | | | | | | | NRS_treat1_  Item 11 |
| Ja | | | | | | | Nein | | | | | | | |
| 11.4 Wie sehr hat die Behandlung Ihre Beeinträchtigung durch die *Kurzatmigkeit* verbessert? | | | | | | | | | | | | | | NRS_treat2_  Item 11 |
| **keine**  **Verbesserung** | 0 | 1 | 2 | 3 | 4 | 5 | | 6 | 7 | 8 | 9 | 10 | **sehr starke Verbesserung** | |
| 11.5 Wie sehr hat die Behandlung Ihre Beeinträchtigung durch die *Kurzatmigkeit* verschlechtert? | | | | | | | | | | | | | | NRS_treat3_  Item 11 |
| **keine Verschlechterung** | 0 | 1 | 2 | 3 | 4 | 5 | | 6 | 7 | 8 | 9 | 10 | **sehr starke Verschlechterung** | |
| 11.6 Wie sehr haben Sie unangenehme Nebenwirkungen durch die Behandlung erlebt? | | | | | | | | | | | | | | TreatExp  Item 11 |
| **keine Nebenwirkungen** | 0 | 1 | 2 | 3 | 4 | 5 | | 6 | 7 | 8 | 9 | 10 | **sehr starke Nebenwirkungen** | |
| **12. Verstopfung. nervöser Darm oder Durchfall** | | | | | | | | | | | | | | PHQ_adapt_  Item 12 |
| **nicht beeinträchtigt** | 0 | 1 | 2 | 3 | 4 | 5 | | 6 | 7 | 8 | 9 | 10 | **sehr stark**  **beeinträchtigt** | |
| 12.2 Wie sehr erwarten Sie bis zum Mittag durch die *Verstopfung. den nervösen Darm oder Durchfall* beeinträchtigt zu sein? | | | | | | | | | | | | | | NRS_expect_  Item 12 |
| **nicht beeinträchtigt** | 0 | 1 | 2 | 3 | 4 | 5 | | 6 | 7 | 8 | 9 | 10 | **sehr stark**  **beeinträchtigt** | |
| 12.3 Haben Sie seit dem letzten Abend etwas unternommen. um die *Verstopfung. den nervösen Darm oder Durchfall* zu behandeln? | | | | | | | | | | | | | | NRS_treat1_  Item 12 |
| Ja | | | | | | | Nein | | | | | | | |
| 12.4 Wie sehr hat die Behandlung Ihre Beeinträchtigung durch die *Verstopfung. den nervösen Darm oder Durchfall* verbessert? | | | | | | | | | | | | | | NRS_treat2_  Item 12 |
| **keine**  **Verbesserung** | 0 | 1 | 2 | 3 | 4 | 5 | | 6 | 7 | 8 | 9 | 10 | **sehr starke Verbesserung** | |
| 12.5 Wie sehr hat die Behandlung Ihre Beeinträchtigung durch die *Verstopfung.* den *nervösen Darm oder Durchfall* verschlechtert? | | | | | | | | | | | | | | NRS_treat3_  Item 12 |
| **keine Verschlechterung** | 0 | 1 | 2 | 3 | 4 | 5 | | 6 | 7 | 8 | 9 | 10 | **sehr starke Verschlechterung** | |
| 12.6 Wie sehr haben Sie unangenehme Nebenwirkungen durch die Behandlung erlebt? | | | | | | | | | | | | | | TreatExp  Item 12 |
| **keine Nebenwirkungen** | 0 | 1 | 2 | 3 | 4 | 5 | | 6 | 7 | 8 | 9 | 10 | **sehr starke Nebenwirkungen** | |
| **13. Übelkeit. Blähungen oder Verdauungsbeschwerden** | | | | | | | | | | | | | | PHQ_adapt_  Item 13 |
| **nicht beeinträchtigt** | 0 | 1 | 2 | 3 | 4 | 5 | | 6 | 7 | 8 | 9 | 10 | **sehr stark**  **beeinträchtigt** | |
| 13.2 Wie sehr erwarten Sie bis zum Mittag durch die *Übelkeit. Blähungen oder Verdauungsbeschwerden* beeinträchtigt zu sein? | | | | | | | | | | | | | | NRS_expect_  Item 13 |
| **nicht beeinträchtigt** | 0 | 1 | 2 | 3 | 4 | 5 | | 6 | 7 | 8 | 9 | 10 | **sehr stark**  **beeinträchtigt** | |
| 13.3 Haben Sie seit dem letzten Abend etwas unternommen. um die *Übelkeit. Blähungen oder Verdauungsbeschwerden* zu behandeln? | | | | | | | | | | | | | | NRS_treat1_  Item 13 |
| Ja | | | | | | | Nein | | | | | | | |
| 13.4 Wie sehr hat die Behandlung Ihre Beeinträchtigung durch die *Übelkeit. Blähungen oder Verdauungsbeschwerden* verbessert? | | | | | | | | | | | | | | NRS_treat2_  Item 13 |
| **keine**  **Verbesserung** | 0 | 1 | 2 | 3 | 4 | 5 | | 6 | 7 | 8 | 9 | 10 | **sehr starke Verbesserung** | |
| 13.5 Wie sehr hat die Behandlung Ihre Beeinträchtigung durch die *Übelkeit. Blähungen oder Verdauungsbeschwerden* verschlechtert? | | | | | | | | | | | | | | NRS_treat3_  Item 13 |
| **keine Verschlechterung** | 0 | 1 | 2 | 3 | 4 | 5 | | 6 | 7 | 8 | 9 | 10 | **sehr starke Verschlechterung** | |
| 13.6 Wie sehr haben Sie unangenehme Nebenwirkungen durch die Behandlung erlebt? | | | | | | | | | | | | | | TreatExp  Item 13 |
| **keine Nebenwirkungen** | 0 | 1 | 2 | 3 | 4 | 5 | | 6 | 7 | 8 | 9 | 10 | **sehr starke Nebenwirkungen** | |
| **14. Müdigkeit oder Gefühl. keine Energie zu haben** | | | | | | | | | | | | | | PHQ_adapt_  Item 14 |
| **nicht beeinträchtigt** | 0 | 1 | 2 | 3 | 4 | 5 | | 6 | 7 | 8 | 9 | 10 | **sehr stark**  **beeinträchtigt** | |
| 14.2 Wie sehr erwarten Sie bis zum Mittag durch die *Müdigkeit oder das Gefühl. keine Energie zu haben.* beeinträchtigt zu sein? | | | | | | | | | | | | | | NRS_expect_  Item 14 |
| **nicht beeinträchtigt** | 0 | 1 | 2 | 3 | 4 | 5 | | 6 | 7 | 8 | 9 | 10 | **sehr stark**  **beeinträchtigt** | |
| 14.3 Haben Sie seit dem letzten Abend etwas unternommen. um die *Müdigkeit oder das Gefühl. keine Energie zu haben.* zu behandeln? | | | | | | | | | | | | | | NRS_treat1_  Item 14 |
| Ja | | | | | | | Nein | | | | | | | |
| 14.4 Wie sehr hat die Behandlung Ihre Beeinträchtigung durch die *Müdigkeit oder das Gefühl. keine Energie zu haben.* verbessert? | | | | | | | | | | | | | | NRS_treat2_  Item 14 |
| **keine**  **Verbesserung** | 0 | 1 | 2 | 3 | 4 | 5 | | 6 | 7 | 8 | 9 | 10 | **sehr starke Verbesserung** | |
| 14.5 Wie sehr hat die Behandlung Ihre Beeinträchtigung durch die *Müdigkeit oder das Gefühl. keine Energie zu haben.* verschlechtert? | | | | | | | | | | | | | | NRS_treat3_  Item 14 |
| **keine Verschlechterung** | 0 | 1 | 2 | 3 | 4 | 5 | | 6 | 7 | 8 | 9 | 10 | **sehr starke Verschlechterung** | |
| 14.6 Wie sehr haben Sie unangenehme Nebenwirkungen durch die Behandlung erlebt? | | | | | | | | | | | | | | TreatExp  Item 14 |
| **keine Nebenwirkungen** | 0 | 1 | 2 | 3 | 4 | 5 | | 6 | 7 | 8 | 9 | 10 | **sehr starke Nebenwirkungen** | |
| **15. Schlafstörungen** | | | | | | | | | | | | | | PHQ_adapt_  Item 15 |
| **nicht beeinträchtigt** | 0 | 1 | 2 | 3 | 4 | 5 | | 6 | 7 | 8 | 9 | 10 | **sehr stark**  **beeinträchtigt** | |
| 15.2 Wie sehr erwarten Sie bis zum Mittag durch die *Schlafstörungen* beeinträchtigt zu sein? | | | | | | | | | | | | | | NRS_expect_  Item 15 |
| **nicht beeinträchtigt** | 0 | 1 | 2 | 3 | 4 | 5 | | 6 | 7 | 8 | 9 | 10 | **sehr stark**  **beeinträchtigt** | |
| 15.3 Haben Sie seit dem letzten Abend etwas unternommen. um die *Schlafstörungen* zu behandeln? | | | | | | | | | | | | | | NRS_treat1_  Item 15 |
| Ja | | | | | | | Nein | | | | | | | |
| 15.4 Wie sehr hat die Behandlung Ihre Beeinträchtigung durch die *Schlafstörungen* verbessert? | | | | | | | | | | | | | | NRS_treat2_  Item 15 |
| **keine**  **Verbesserung** | 0 | 1 | 2 | 3 | 4 | 5 | | 6 | 7 | 8 | 9 | 10 | **sehr starke Verbesserung** | |
| 15.5 Wie sehr hat die Behandlung Ihre Beeinträchtigung durch die *Schlafstörungen* verschlechtert? | | | | | | | | | | | | | | NRS_treat3_  Item 15 |
| **keine Verschlechterung** | 0 | 1 | 2 | 3 | 4 | 5 | | 6 | 7 | 8 | 9 | 10 | **sehr starke Verschlechterung** | |
| 15.6 Wie sehr haben Sie unangenehme Nebenwirkungen durch die Behandlung erlebt? | | | | | | | | | | | | | | TreatExp  Item 15 |
| **keine Nebenwirkungen** | 0 | 1 | 2 | 3 | 4 | 5 | | 6 | 7 | 8 | 9 | 10 | **sehr starke Nebenwirkungen** | |

**Adapted Questionnaire (PHQ_adapt_): Positive Expectation Framing. Morning Assessment**

**Online-Befragung am Morgen** (8:00 Uhr bis 10:00 Uhr)

**Einleitung:** *„Herzlich willkommen zur Online-Befragung der SymTrack 2.0 Pilotstudie. Vielen Dank, dass Sie sich heute Morgen Zeit dafür nehmen.“*

| Im folgenden Abschnitt geht es darum, einzuschätzen, durch welche körperlichen Beschwerden Sie seit dem letzten Abend beeinträchtigt sind, welche Erwartungen Sie bezüglich des Verlaufs der Beschwerden haben und ob Sie bereits etwas gegen die Beschwerden unternommen haben: | | | | | | | | | | | | | | |
| --- | --- | --- | --- | --- | --- | --- | --- | --- | --- | --- | --- | --- | --- | --- |
| **Wie stark fühlen Sie sich seit dem letzten Abend durch die folgenden Beschwerden beeinträchtigt?** | | | | | | | | | | | | | | |
| **1. Bauchschmerzen** | | | | | | | | | | | | | | PHQ_adapt_  Item 1 |
| **nicht beeinträchtigt** | 0 | 1 | 2 | 3 | 4 | 5 | | 6 | 7 | 8 | 9 | 10 | **sehr stark**  **beeinträchtigt** | |
| 1.2 Wie sehr erwarten Sie bis zum Mittag hinsichtlich der Bauchschmerzen beeinträchtigungsfrei zu sein? | | | | | | | | | | | | | | NRS_expect#_  Item 1 |
| **gar nicht beeinträchtigungsfrei** | 0 | 1 | 2 | 3 | 4 | 5 | | 6 | 7 | 8 | 9 | 10 | **sehr beeinträchtigungsfrei** | |
| 1.3 Haben Sie seit dem letzten Abend etwas unternommen, um die *Bauchschmerzen* zu behandeln? | | | | | | | | | | | | | | NRS_treat1_  Item 1 |
| Ja | | | | | | | Nein | | | | | | | |
| 1.4 Wie sehr hat die Behandlung Ihre Beeinträchtigung durch die *Bauchschmerzen* verbessert? | | | | | | | | | | | | | | NRS_treat2_  Item 1 |
| **keine**  **Verbesserung** | 0 | 1 | 2 | 3 | 4 | 5 | | 6 | 7 | 8 | 9 | 10 | **sehr starke Verbesserung** | |
| 1.5 Wie sehr hat die Behandlung Ihre Beeinträchtigung durch die *Bauchschmerzen* verschlechtert? | | | | | | | | | | | | | | NRS_treat3_  Item 1 |
| **keine Verschlechterung** | 0 | 1 | 2 | 3 | 4 | 5 | | 6 | 7 | 8 | 9 | 10 | **sehr starke Verschlechterung** | |
| 1.6 Wie sehr haben Sie unangenehme Nebenwirkungen durch die Behandlung erlebt? | | | | | | | | | | | | | | TreatExp  Item 1 |
| **keine Nebenwirkungen** | 0 | 1 | 2 | 3 | 4 | 5 | | 6 | 7 | 8 | 9 | 10 | **sehr starke Nebenwirkungen** | |
| **2. Rückenschmerzen** | | | | | | | | | | | | | | PHQ_adapt_  Item 2 |
| **nicht beeinträchtigt** | 0 | 1 | 2 | 3 | 4 | 5 | | 6 | 7 | 8 | 9 | 10 | **sehr stark**  **beeinträchtigt** | |
| 2.2 Wie sehr erwarten Sie bis zum Mittag hinsichtlich der *Rückenschmerzen* beeiträchtigungsfrei zu sein? | | | | | | | | | | | | | | NRS_expect#_  Item 2 |
| **gar nicht beeinträchtigungsfrei** | 0 | 1 | 2 | 3 | 4 | 5 | | 6 | 7 | 8 | 9 | 10 | **sehr beeinträchtigungsfrei** | |
| 2.3 Haben Sie seit dem letzten Abend etwas unternommen, um die *Rückenschmerzen* zu behandeln? | | | | | | | | | | | | | | NRS_treat1_  Item 2 |
| Ja | | | | | | | Nein | | | | | | | |
| 2.4 Wie sehr hat die Behandlung Ihre Beeinträchtigung durch die *Rückenschmerzen* verbessert? | | | | | | | | | | | | | | NRS_treat2_  Item 2 |
| **keine**  **Verbesserung** | 0 | 1 | 2 | 3 | 4 | 5 | | 6 | 7 | 8 | 9 | 10 | **sehr starke Verbesserung** | |
| 2.5 Wie sehr hat die Behandlung Ihre Beeinträchtigung durch die *Rückenschmerzen* verschlechtert? | | | | | | | | | | | | | | NRS_treat3_  Item 2 |
| **keine Verschlechterung** | 0 | 1 | 2 | 3 | 4 | 5 | | 6 | 7 | 8 | 9 | 10 | **sehr starke Verschlechterung** | |
| 2.6 Wie sehr haben Sie unangenehme Nebenwirkungen durch die Behandlung erlebt? | | | | | | | | | | | | | | TreatExp  Item 2 |
| **keine Nebenwirkungen** | 0 | 1 | 2 | 3 | 4 | 5 | | 6 | 7 | 8 | 9 | 10 | **sehr starke Nebenwirkungen** | |
| **3. Schmerzen in Armen, Beinen oder Gelenken (Knie, Hüften, usw.)** | | | | | | | | | | | | | | PHQ_adapt_  Item 3 |
| **nicht beeinträchtigt** | 0 | 1 | 2 | 3 | 4 | 5 | | 6 | 7 | 8 | 9 | 10 | **sehr stark**  **beeinträchtigt** | |
| 3.2 Wie sehr erwarten Sie bis zum Mittag hinsichtlich der *Schmerzen in Armen, Beinen oder Gelenken (Knie, Hüften, usw.)* beeinträchtigungsfrei zu sein? | | | | | | | | | | | | | | NRS_expect#_  Item 3 |
| **gar nicht beeinträchtigungsfrei** | 0 | 1 | 2 | 3 | 4 | 5 | | 6 | 7 | 8 | 9 | 10 | **sehr beeinträchtigungsfrei** | |
| 3.3 Haben Sie seit dem letzten Abend etwas unternommen, um die *Schmerzen in Armen, Beinen oder Gelenken (Knie, Hüften, usw.)* zu behandeln? | | | | | | | | | | | | | | NRS_treat1_  Item 3 |
| Ja | | | | | | | Nein | | | | | | | |
| 3.4 Wie sehr hat die Behandlung Ihre Beeinträchtigung durch die *Schmerzen in Armen, Beinen oder Gelenken (Knie, Hüften, usw.)* verbessert? | | | | | | | | | | | | | | NRS_treat2_  Item 3 |
| **keine**  **Verbesserung** | 0 | 1 | 2 | 3 | 4 | 5 | | 6 | 7 | 8 | 9 | 10 | **sehr starke Verbesserung** | |
| 3.5 Wie sehr hat die Behandlung Ihre Beeinträchtigung durch die *Schmerzen in Armen, Beinen oder Gelenken (Knie, Hüften, usw.)* verschlechtert? | | | | | | | | | | | | | | NRS_treat3_  Item 3 |
| **keine Verschlechterung** | 0 | 1 | 2 | 3 | 4 | 5 | | 6 | 7 | 8 | 9 | 10 | **sehr starke Verschlechterung** | |
| 3.6 Wie sehr haben Sie unangenehme Nebenwirkungen durch die Behandlung erlebt? | | | | | | | | | | | | | | TreatExp  Item 3 |
| **keine Nebenwirkungen** | 0 | 1 | 2 | 3 | 4 | 5 | | 6 | 7 | 8 | 9 | 10 | **sehr starke Nebenwirkungen** | |
| **4. Menstruationsschmerzen oder andere Probleme mit der Menstruation** | | | | | | | | | | | | | | PHQ_adapt_  Item 4 |
| **nicht beeinträchtigt** | 0 | 1 | 2 | 3 | 4 | 5 | | 6 | 7 | 8 | 9 | 10 | **sehr stark**  **beeinträchtigt** | |
| 4.2 Wie sehr erwarten Sie bis zum Mittag hinsichtlich der *Menstruationsschmerzen oder anderer Probleme mit der Menstruation* beeinträchtigungsfrei zu sein? | | | | | | | | | | | | | | NRS_expect#_  Item 4 |
| **gar nicht beeinträchtigungsfrei** | 0 | 1 | 2 | 3 | 4 | 5 | | 6 | 7 | 8 | 9 | 10 | **sehr beeinträchtigungsfrei** | |
| 4.3 Haben Sie seit dem letzten Abend etwas unternommen, um die *Menstruationsschmerzen oder andere Probleme mit der Menstruation* zu behandeln? | | | | | | | | | | | | | | NRS_treat1_  Item 4 |
| Ja | | | | | | | Nein | | | | | | | |
| 4.4 Wie sehr hat die Behandlung Ihre Beeinträchtigung durch die *Menstruationsschmerzen oder andere Probleme mit der Menstruation* verbessert? | | | | | | | | | | | | | | NRS_treat2_  Item 4 |
| **keine**  **Verbesserung** | 0 | 1 | 2 | 3 | 4 | 5 | | 6 | 7 | 8 | 9 | 10 | **sehr starke Verbesserung** | |
| 4.5 Wie sehr hat die Behandlung Ihre Beeinträchtigung durch die *Menstruationsschmerzen oder andere Probleme mit der Menstruation* verschlechtert? | | | | | | | | | | | | | | NRS_treat3_  Item 4 |
| **keine Verschlechterung** | 0 | 1 | 2 | 3 | 4 | 5 | | 6 | 7 | 8 | 9 | 10 | **sehr starke Verschlechterung** | |
| 4.6 Wie sehr haben Sie unangenehme Nebenwirkungen durch die Behandlung erlebt? | | | | | | | | | | | | | | TreatExp  Item 4 |
| **keine Nebenwirkungen** | 0 | 1 | 2 | 3 | 4 | 5 | | 6 | 7 | 8 | 9 | 10 | **sehr starke Nebenwirkungen** | |
| **5. Schmerzen oder Probleme beim Geschlechtsverkehr** | | | | | | | | | | | | | | PHQ_adapt_  Item 5 |
| **nicht beeinträchtigt** | 0 | 1 | 2 | 3 | 4 | 5 | | 6 | 7 | 8 | 9 | 10 | **sehr stark**  **beeinträchtigt** | |
| 5.2 Wie sehr erwarten Sie bis zum Mittag hinsichtlich der *Schmerzen oder Probleme beim Geschlechtsverkehr* beeinträchtigungsfrei zu sein? | | | | | | | | | | | | | | NRS_expect#_  Item 5 |
| **gar nicht beeinträchtigungsfrei** | 0 | 1 | 2 | 3 | 4 | 5 | | 6 | 7 | 8 | 9 | 10 | **sehr beeinträchtigungsfrei** | |
| 5.3 Haben Sie seit dem letzten Abend etwas unternommen, um die *Schmerzen oder Probleme beim Geschlechtsverkehr* zu behandeln? | | | | | | | | | | | | | | NRS_treat1_  Item 5 |
| Ja | | | | | | | Nein | | | | | | | |
| 5.4 Wie sehr hat die Behandlung Ihre Beeinträchtigung durch die *Schmerzen oder Probleme beim Geschlechtsverkehr* verbessert? | | | | | | | | | | | | | | NRS_treat2_  Item 5 |
| **keine**  **Verbesserung** | 0 | 1 | 2 | 3 | 4 | 5 | | 6 | 7 | 8 | 9 | 10 | **sehr starke Verbesserung** | |
| 5.5 Wie sehr hat die Behandlung Ihre Beeinträchtigung durch die *Schmerzen oder Probleme beim Geschlechtsverkehr* verschlechtert? | | | | | | | | | | | | | | NRS_treat3_  Item 5 |
| **keine Verschlechterung** | 0 | 1 | 2 | 3 | 4 | 5 | | 6 | 7 | 8 | 9 | 10 | **sehr starke Verschlechterung** | |
| 5.6 Wie sehr haben Sie unangenehme Nebenwirkungen durch die Behandlung erlebt? | | | | | | | | | | | | | | TreatExp  Item 5 |
| **keine Nebenwirkungen** | 0 | 1 | 2 | 3 | 4 | 5 | | 6 | 7 | 8 | 9 | 10 | **sehr starke Nebenwirkungen** | |
| **6. Kopfschmerzen** | | | | | | | | | | | | | | PHQ_adapt_  Item 6 |
| **nicht beeinträchtigt** | 0 | 1 | 2 | 3 | 4 | 5 | | 6 | 7 | 8 | 9 | 10 | **sehr stark**  **beeinträchtigt** | |
| 6.2 Wie sehr erwarten Sie bis zum Mittag hinsichtlich der *Kopfschmerzen* beeinträchtigungsfrei zu sein? | | | | | | | | | | | | | | NRS_expect#_  Item 6 |
| **gar nicht beeinträchtigungsfrei** | 0 | 1 | 2 | 3 | 4 | 5 | | 6 | 7 | 8 | 9 | 10 | **sehr beeinträchtigungsfrei** | |
| 6.3 Haben Sie seit dem letzten Abend etwas unternommen, um die *Kopfschmerzen* zu behandeln? | | | | | | | | | | | | | | NRS_treat1_  Item 6 |
| Ja | | | | | | | Nein | | | | | | | |
| 6.4 Wie sehr hat die Behandlung Ihre Beeinträchtigung durch die *Kopfschmerzen* verbessert? | | | | | | | | | | | | | | NRS_treat2_  Item 6 |
| **keine**  **Verbesserung** | 0 | 1 | 2 | 3 | 4 | 5 | | 6 | 7 | 8 | 9 | 10 | **sehr starke Verbesserung** | |
| 6.5 Wie sehr hat die Behandlung Ihre Beeinträchtigung durch die *Kopfschmerzen* verschlechtert? | | | | | | | | | | | | | | NRS_treat3_  Item 6 |
| **keine Verschlechterung** | 0 | 1 | 2 | 3 | 4 | 5 | | 6 | 7 | 8 | 9 | 10 | **sehr starke Verschlechterung** | |
| 6.6 Wie sehr haben Sie unangenehme Nebenwirkungen durch die Behandlung erlebt? | | | | | | | | | | | | | | TreatExp  Item 6 |
| **keine Nebenwirkungen** | 0 | 1 | 2 | 3 | 4 | 5 | | 6 | 7 | 8 | 9 | 10 | **sehr starke Nebenwirkungen** | |
| **7. Schmerzen im Brustbereich** | | | | | | | | | | | | | | PHQ_adapt_  Item 7 |
| **nicht beeinträchtigt** | 0 | 1 | 2 | 3 | 4 | 5 | | 6 | 7 | 8 | 9 | 10 | **sehr stark**  **beeinträchtigt** | |
| 7.2 Wie sehr erwarten Sie bis zum Mittag hinsichtlich der *Schmerzen im Brustbereich* beeinträchtigungsfrei zu sein? | | | | | | | | | | | | | | NRS_expect#_  Item 7 |
| **gar nicht beeinträchtigungsfrei** | 0 | 1 | 2 | 3 | 4 | 5 | | 6 | 7 | 8 | 9 | 10 | **sehr beeinträchtigungsfrei** | |
| 7.3 Haben Sie seit dem letzten Abend etwas unternommen, um die *Schmerzen im Brustbereich* zu behandeln? | | | | | | | | | | | | | | NRS_treat1_  Item 7 |
| Ja | | | | | | | Nein | | | | | | | |
| 7.4 Wie sehr hat die Behandlung Ihre Beeinträchtigung durch die *Schmerzen im Brustbereich* verbessert? | | | | | | | | | | | | | | NRS_treat2_  Item 7 |
| **keine**  **Verbesserung** | 0 | 1 | 2 | 3 | 4 | 5 | | 6 | 7 | 8 | 9 | 10 | **sehr starke Verbesserung** | |
| 7.5 Wie sehr hat die Behandlung Ihre Beeinträchtigung durch die *Schmerzen im Brustbereich* verschlechtert? | | | | | | | | | | | | | | NRS_treat3_  Item 7 |
| **keine Verschlechterung** | 0 | 1 | 2 | 3 | 4 | 5 | | 6 | 7 | 8 | 9 | 10 | **sehr starke Verschlechterung** | |
| 7.6 Wie sehr haben Sie unangenehme Nebenwirkungen durch die Behandlung erlebt? | | | | | | | | | | | | | | TreatExp  Item 7 |
| **keine Nebenwirkungen** | 0 | 1 | 2 | 3 | 4 | 5 | | 6 | 7 | 8 | 9 | 10 | **sehr starke Nebenwirkungen** | |
| **8. Schwindel** | | | | | | | | | | | | | | PHQ_adapt_  Item 8 |
| **nicht beeinträchtigt** | 0 | 1 | 2 | 3 | 4 | 5 | | 6 | 7 | 8 | 9 | 10 | **sehr stark**  **beeinträchtigt** | |
| 8.2 Wie sehr erwarten Sie bis zum Mittag hinsichtlich des *Schwindels* beeinträchtigungsfrei zu sein? | | | | | | | | | | | | | | NRS_expect#_  Item 8 |
| **gar nicht beeinträchtigungsfrei** | 0 | 1 | 2 | 3 | 4 | 5 | | 6 | 7 | 8 | 9 | 10 | **sehr beeinträchtigungsfrei** | |
| 8.3 Haben Sie seit dem letzten Abend etwas unternommen, um den *Schwindel* zu behandeln? | | | | | | | | | | | | | | NRS_treat1_  Item 8 |
| Ja | | | | | | | Nein | | | | | | | |
| 8.4 Wie sehr hat die Behandlung Ihre Beeinträchtigung durch den *Schwindel* verbessert? | | | | | | | | | | | | | | NRS_treat2_  Item 8 |
| **keine**  **Verbesserung** | 0 | 1 | 2 | 3 | 4 | 5 | | 6 | 7 | 8 | 9 | 10 | **sehr starke Verbesserung** | |
| 8.5 Wie sehr hat die Behandlung Ihre Beeinträchtigung durch den *Schwindel* verschlechtert? | | | | | | | | | | | | | | NRS_treat3_  Item 8 |
| **keine Verschlechterung** | 0 | 1 | 2 | 3 | 4 | 5 | | 6 | 7 | 8 | 9 | 10 | **sehr starke Verschlechterung** | |
| 8.6 Wie sehr haben Sie unangenehme Nebenwirkungen durch die Behandlung erlebt? | | | | | | | | | | | | | | TreatExp  Item 8 |
| **keine Nebenwirkungen** | 0 | 1 | 2 | 3 | 4 | 5 | | 6 | 7 | 8 | 9 | 10 | **sehr starke Nebenwirkungen** | |
| **9. Ohnmachtsanfälle** | | | | | | | | | | | | | | PHQ_adapt_  Item 9 |
| **nicht beeinträchtigt** | 0 | 1 | 2 | 3 | 4 | 5 | | 6 | 7 | 8 | 9 | 10 | **sehr stark**  **beeinträchtigt** | |
| 9.2 Wie sehr erwarten Sie bis zum Mittag hinsichtlich der *Ohnmachtsanfälle* beeinträchtigungsfrei zu sein? | | | | | | | | | | | | | | NRS_expect#_  Item 9 |
| **gar nicht beeinträchtigungsfrei** | 0 | 1 | 2 | 3 | 4 | 5 | | 6 | 7 | 8 | 9 | 10 | **sehr beeinträchtigungsfrei** | |
| 9.3 Haben Sie seit dem letzten Abend etwas unternommen, um die *Ohnmachtsanfälle* zu behandeln? | | | | | | | | | | | | | | NRS_treat1_  Item 9 |
| Ja | | | | | | | Nein | | | | | | | |
| 9.4 Wie sehr hat die Behandlung Ihre Beeinträchtigung durch die *Ohnmachtsanfälle* verbessert? | | | | | | | | | | | | | | NRS_treat2_  Item 9 |
| **keine**  **Verbesserung** | 0 | 1 | 2 | 3 | 4 | 5 | | 6 | 7 | 8 | 9 | 10 | **sehr starke Verbesserung** | |
| 9.5 Wie sehr hat die Behandlung Ihre Beeinträchtigung durch die *Ohnmachtsanfälle* verschlechtert? | | | | | | | | | | | | | | NRS_treat3_  Item 9 |
| **keine Verschlechterung** | 0 | 1 | 2 | 3 | 4 | 5 | | 6 | 7 | 8 | 9 | 10 | **sehr starke Verschlechterung** | |
| 9.6 Wie sehr haben Sie unangenehme Nebenwirkungen durch die Behandlung erlebt? | | | | | | | | | | | | | | TreatExp  Item 9 |
| **keine Nebenwirkungen** | 0 | 1 | 2 | 3 | 4 | 5 | | 6 | 7 | 8 | 9 | 10 | **sehr starke Nebenwirkungen** | |
| **10. Herzklopfen oder Herzrasen** | | | | | | | | | | | | | | PHQ_adapt_  Item 10 |
| **nicht beeinträchtigt** | 0 | 1 | 2 | 3 | 4 | 5 | | 6 | 7 | 8 | 9 | 10 | **sehr stark**  **beeinträchtigt** | |
| 10.2 Wie sehr erwarten Sie bis zum Mittag hinsichtlich des *Herzklopfens oder Herzrasens* beeinträchtigungsfrei zu sein? | | | | | | | | | | | | | | NRS_expect#_  Item 10 |
| **gar nicht beeinträchtigungsfrei** | 0 | 1 | 2 | 3 | 4 | 5 | | 6 | 7 | 8 | 9 | 10 | **sehr beeinträchtigungsfrei** | |
| 10.3 Haben Sie seit dem letzten Abend etwas unternommen, um das *Herzklopfen oder Herzrasen* zu behandeln? | | | | | | | | | | | | | | NRS_treat1_  Item 10 |
| Ja | | | | | | | Nein | | | | | | | |
| 10.4 Wie sehr hat die Behandlung Ihre Beeinträchtigung durch das *Herzklopfen oder Herzrasen* verbessert? | | | | | | | | | | | | | | NRS_treat2_  Item 10 |
| **keine**  **Verbesserung** | 0 | 1 | 2 | 3 | 4 | 5 | | 6 | 7 | 8 | 9 | 10 | **sehr starke Verbesserung** | |
| 10.5 Wie sehr hat die Behandlung Ihre Beeinträchtigung durch das *Herzklopfen oder Herzrasen* verschlechtert? | | | | | | | | | | | | | | NRS_treat3_  Item 10 |
| **keine Verschlechterung** | 0 | 1 | 2 | 3 | 4 | 5 | | 6 | 7 | 8 | 9 | 10 | **sehr starke Verschlechterung** | |
| 10.6 Wie sehr haben Sie unangenehme Nebenwirkungen durch die Behandlung erlebt? | | | | | | | | | | | | | | TreatExp  Item 10 |
| **keine Nebenwirkungen** | 0 | 1 | 2 | 3 | 4 | 5 | | 6 | 7 | 8 | 9 | 10 | **sehr starke Nebenwirkungen** | |
| **11. Kurzatmigkeit** | | | | | | | | | | | | | | PHQ_adapt_  Item 11 |
| **nicht beeinträchtigt** | 0 | 1 | 2 | 3 | 4 | 5 | | 6 | 7 | 8 | 9 | 10 | **sehr stark**  **beeinträchtigt** | |
| 11.2 Wie sehr erwarten Sie bis zum Mittag hinsichtlich der *Kurzatmigkeit* beeinträchtigungsfrei zu sein? | | | | | | | | | | | | | | NRS_expect#_  Item 11 |
| **gar nicht beeinträchtigungsfrei** | 0 | 1 | 2 | 3 | 4 | 5 | | 6 | 7 | 8 | 9 | 10 | **sehr beeinträchtigungsfrei** | |
| 11.3 Haben Sie seit dem letzten Abend etwas unternommen, um die *Kurzatmigkeit* zu behandeln? | | | | | | | | | | | | | | NRS_treat1_  Item 11 |
| Ja | | | | | | | Nein | | | | | | | |
| 11.4 Wie sehr hat die Behandlung Ihre Beeinträchtigung durch die *Kurzatmigkeit* verbessert? | | | | | | | | | | | | | | NRS_treat2_  Item 11 |
| **keine**  **Verbesserung** | 0 | 1 | 2 | 3 | 4 | 5 | | 6 | 7 | 8 | 9 | 10 | **sehr starke Verbesserung** | |
| 11.5 Wie sehr hat die Behandlung Ihre Beeinträchtigung durch die *Kurzatmigkeit* verschlechtert? | | | | | | | | | | | | | | NRS_treat3_  Item 11 |
| **keine Verschlechterung** | 0 | 1 | 2 | 3 | 4 | 5 | | 6 | 7 | 8 | 9 | 10 | **sehr starke Verschlechterung** | |
| 11.6 Wie sehr haben Sie unangenehme Nebenwirkungen durch die Behandlung erlebt? | | | | | | | | | | | | | | TreatExp  Item 11 |
| **keine Nebenwirkungen** | 0 | 1 | 2 | 3 | 4 | 5 | | 6 | 7 | 8 | 9 | 10 | **sehr starke Nebenwirkungen** | |
| **12. Verstopfung, nervöser Darm oder Durchfall** | | | | | | | | | | | | | | PHQ_adapt_  Item 12 |
| **nicht beeinträchtigt** | 0 | 1 | 2 | 3 | 4 | 5 | | 6 | 7 | 8 | 9 | 10 | **sehr stark**  **beeinträchtigt** | |
| 12.2 Wie sehr erwarten Sie bis zum Mittag hinsichtlich der *Verstopfung, des nervösen Darms oder Durchfalls* beeinträchtigungsfrei zu sein? | | | | | | | | | | | | | | NRS_expect#_  Item 12 |
| **gar nicht beeinträchtigungsfrei** | 0 | 1 | 2 | 3 | 4 | 5 | | 6 | 7 | 8 | 9 | 10 | **sehr beeinträchtigungsfrei** | |
| 12.3 Haben Sie seit dem letzten Abend etwas unternommen, um die *Verstopfung, den nervösen Darm oder Durchfall* zu behandeln? | | | | | | | | | | | | | | NRS_treat1_  Item 12 |
| Ja | | | | | | | Nein | | | | | | | |
| 12.4 Wie sehr hat die Behandlung Ihre Beeinträchtigung durch die *Verstopfung, den nervösen Darm oder Durchfall* verbessert? | | | | | | | | | | | | | | NRS_treat2_  Item 12 |
| **keine**  **Verbesserung** | 0 | 1 | 2 | 3 | 4 | 5 | | 6 | 7 | 8 | 9 | 10 | **sehr starke Verbesserung** | |
| 12.5 Wie sehr hat die Behandlung Ihre Beeinträchtigung durch die *Verstopfung,* den *nervösen Darm oder Durchfall* verschlechtert? | | | | | | | | | | | | | | NRS_treat3_  Item 12 |
| **keine Verschlechterung** | 0 | 1 | 2 | 3 | 4 | 5 | | 6 | 7 | 8 | 9 | 10 | **sehr starke Verschlechterung** | |
| 12.6 Wie sehr haben Sie unangenehme Nebenwirkungen durch die Behandlung erlebt? | | | | | | | | | | | | | | TreatExp  Item 12 |
| **keine Nebenwirkungen** | 0 | 1 | 2 | 3 | 4 | 5 | | 6 | 7 | 8 | 9 | 10 | **sehr starke Nebenwirkungen** | |
| **13. Übelkeit, Blähungen oder Verdauungsbeschwerden** | | | | | | | | | | | | | | PHQ_adapt_  Item 13 |
| **nicht beeinträchtigt** | 0 | 1 | 2 | 3 | 4 | 5 | | 6 | 7 | 8 | 9 | 10 | **sehr stark**  **beeinträchtigt** | |
| 13.2 Wie sehr erwarten Sie bis zum Mittag hinsichtlich der *Übelkeit, Blähungen oder Verdauungsbeschwerden* beeinträchtigungsfrei zu sein? | | | | | | | | | | | | | | NRS_expect#_  Item 13 |
| **gar nicht beeinträchtigungsfrei** | 0 | 1 | 2 | 3 | 4 | 5 | | 6 | 7 | 8 | 9 | 10 | **sehr beeinträchtigungsfrei** | |
| 13.3 Haben Sie seit dem letzten Abend etwas unternommen, um die *Übelkeit, Blähungen oder Verdauungsbeschwerden* zu behandeln? | | | | | | | | | | | | | | NRS_treat1_  Item 13 |
| Ja | | | | | | | Nein | | | | | | | |
| 13.4 Wie sehr hat die Behandlung Ihre Beeinträchtigung durch die *Übelkeit, Blähungen oder Verdauungsbeschwerden* verbessert? | | | | | | | | | | | | | | NRS_treat2_  Item 13 |
| **keine**  **Verbesserung** | 0 | 1 | 2 | 3 | 4 | 5 | | 6 | 7 | 8 | 9 | 10 | **sehr starke Verbesserung** | |
| 13.5 Wie sehr hat die Behandlung Ihre Beeinträchtigung durch die *Übelkeit, Blähungen oder Verdauungsbeschwerden* verschlechtert? | | | | | | | | | | | | | | NRS_treat3_  Item 13 |
| **keine Verschlechterung** | 0 | 1 | 2 | 3 | 4 | 5 | | 6 | 7 | 8 | 9 | 10 | **sehr starke Verschlechterung** | |
| 13.6 Wie sehr haben Sie unangenehme Nebenwirkungen durch die Behandlung erlebt? | | | | | | | | | | | | | | TreatExp  Item 13 |
| **keine Nebenwirkungen** | 0 | 1 | 2 | 3 | 4 | 5 | | 6 | 7 | 8 | 9 | 10 | **sehr starke Nebenwirkungen** | |
| **14. Müdigkeit oder Gefühl, keine Energie zu haben** | | | | | | | | | | | | | | PHQ_adapt_  Item 14 |
| **nicht beeinträchtigt** | 0 | 1 | 2 | 3 | 4 | 5 | | 6 | 7 | 8 | 9 | 10 | **sehr stark**  **beeinträchtigt** | |
| 14.2 Wie sehr erwarten Sie bis zum Mittag hinsichtlich der *Müdigkeit oder dem Gefühl, keine Energie zu haben,* beeinträchtigungsfrei zu sein? | | | | | | | | | | | | | | NRS_expect#_  Item 14 |
| **gar nicht beeinträchtigungsfrei** | 0 | 1 | 2 | 3 | 4 | 5 | | 6 | 7 | 8 | 9 | 10 | **sehr beeinträchtigungsfrei** | |
| 14.3 Haben Sie seit dem letzten Abend etwas unternommen, um die *Müdigkeit oder das Gefühl, keine Energie zu haben,* zu behandeln? | | | | | | | | | | | | | | NRS_treat1_  Item 14 |
| Ja | | | | | | | Nein | | | | | | | |
| 14.4 Wie sehr hat die Behandlung Ihre Beeinträchtigung durch die *Müdigkeit oder das Gefühl, keine Energie zu haben,* verbessert? | | | | | | | | | | | | | | NRS_treat2_  Item 14 |
| **keine**  **Verbesserung** | 0 | 1 | 2 | 3 | 4 | 5 | | 6 | 7 | 8 | 9 | 10 | **sehr starke Verbesserung** | |
| 14.5 Wie sehr hat die Behandlung Ihre Beeinträchtigung durch die *Müdigkeit oder das Gefühl, keine Energie zu haben,* verschlechtert? | | | | | | | | | | | | | | NRS_treat3_  Item 14 |
| **keine Verschlechterung** | 0 | 1 | 2 | 3 | 4 | 5 | | 6 | 7 | 8 | 9 | 10 | **sehr starke Verschlechterung** | |
| 14.6 Wie sehr haben Sie unangenehme Nebenwirkungen durch die Behandlung erlebt? | | | | | | | | | | | | | | TreatExp  Item 14 |
| **keine Nebenwirkungen** | 0 | 1 | 2 | 3 | 4 | 5 | | 6 | 7 | 8 | 9 | 10 | **sehr starke Nebenwirkungen** | |
| **15. Schlafstörungen** | | | | | | | | | | | | | | PHQ_adapt_  Item 15 |
| **nicht beeinträchtigt** | 0 | 1 | 2 | 3 | 4 | 5 | | 6 | 7 | 8 | 9 | 10 | **sehr stark**  **beeinträchtigt** | |
| 15.2 Wie sehr erwarten Sie bis zum Mittag hinsichtlich der *Schlafstörungen* beeinträchtigungsfrei zu sein? | | | | | | | | | | | | | | NRS_expect#_  Item 15 |
| **gar nicht beeinträchtigungsfrei** | 0 | 1 | 2 | 3 | 4 | 5 | | 6 | 7 | 8 | 9 | 10 | **sehr beeinträchtigungsfrei** | |
| 15.3 Haben Sie seit dem letzten Abend etwas unternommen, um die *Schlafstörungen* zu behandeln? | | | | | | | | | | | | | | NRS_treat1_  Item 15 |
| Ja | | | | | | | Nein | | | | | | | |
| 15.4 Wie sehr hat die Behandlung Ihre Beeinträchtigung durch die *Schlafstörungen* verbessert? | | | | | | | | | | | | | | NRS_treat2_  Item 15 |
| **keine**  **Verbesserung** | 0 | 1 | 2 | 3 | 4 | 5 | | 6 | 7 | 8 | 9 | 10 | **sehr starke Verbesserung** | |
| 15.5 Wie sehr hat die Behandlung Ihre Beeinträchtigung durch die *Schlafstörungen* verschlechtert? | | | | | | | | | | | | | | NRS_treat3_  Item 15 |
| **keine Verschlechterung** | 0 | 1 | 2 | 3 | 4 | 5 | | 6 | 7 | 8 | 9 | 10 | **sehr starke Verschlechterung** | |
| 15.6 Wie sehr haben Sie unangenehme Nebenwirkungen durch die Behandlung erlebt? | | | | | | | | | | | | | | TreatExp  Item 15 |
| **keine Nebenwirkungen** | 0 | 1 | 2 | 3 | 4 | 5 | | 6 | 7 | 8 | 9 | 10 | **sehr starke Nebenwirkungen** | |

**Abschluss:** *„Jetzt haben Sie es geschafft! Wir bedanken uns für die Beantwortung der Fragen und bitten Sie, die Fragen bei der nächsten Befragung am Mittag erneut zu beantworten, da uns vor allem der Verlauf Ihrer Beschwerden interessiert.“*

**English Translation: Adapted Questionnaire (PHQ_adapt_): Negative Expectation Framing. Morning Assessment**

**Ecological Momentary Assessment (EMA)**

**Framing-Group:** Standard

**Online-survey: Morning**

**Introduction: *“****Welcome to this online survey of our study. Thank you for taking the time to participate this morning.”*

| The following section focuses on assessing which physical symptoms are affecting you right now, what expectations you have regarding the impairment due to these symptoms, and whether you have already taken any steps to address them. | | | | | | | | | | | | | | |
| --- | --- | --- | --- | --- | --- | --- | --- | --- | --- | --- | --- | --- | --- | --- |
| **At the moment, how much are you bothered by the following symptoms?** | | | | | | | | | | | | | | |
| **1. Stomach pain** | | | | | | | | | | | | | | PHQ_adapt_  Item 1 |
| **not bothered at all** | 0 | 1 | 2 | 3 | 4 | 5 | | 6 | 7 | 8 | 9 | 10 | **bothered a lot** | |
| 1.2 How impaired do you expect to be by your *Stomach pain* by midday? | | | | | | | | | | | | | | NRS_expect_  Item 1 |
| **not impaired** | 0 | 1 | 2 | 3 | 4 | 5 | | 6 | 7 | 8 | 9 | 10 | **very strongly impairment** | |
| 1.3 Have you received or self-administered any treatment(s) for your *Stomach pain* since last evening? | | | | | | | | | | | | | | NRS_treat1_  Item 1 |
| Yes | | | | | | | No | | | | | | | |
| 1.4 If yes, how much did the treatment improve your impairment from the *Stomach pain?* | | | | | | | | | | | | | | NRS_treat2_  Item 1 |
| **no improvement** | 0 | 1 | 2 | 3 | 4 | 5 | | 6 | 7 | 8 | 9 | 10 | **very strong improvement** | |
| 1.5 How much did the treatment(s) worsen your impairment from the *Stomach pain*? | | | | | | | | | | | | | | NRS_treat3_  Item 1 |
| **no worsening** | 0 | 1 | 2 | 3 | 4 | 5 | | 6 | 7 | 8 | 9 | 10 | **very strong worsening** | |
| 1.6 How much did you experience adverse side effects from this treatment(s)? | | | | | | | | | | | | | | TreatExp  Item 1 |
| **no side effects** | 0 | 1 | 2 | 3 | 4 | 5 | | 6 | 7 | 8 | 9 | 10 | **very strong side effects** | |
| **2. Back pain** | | | | | | | | | | | | | | PHQ_adapt_  Item 2 |
| **not bothered at all** | 0 | 1 | 2 | 3 | 4 | 5 | | 6 | 7 | 8 | 9 | 10 | **bothered a lot** | |
| 2.2 How impaired do you expect to be by your *Back pain* by midday? | | | | | | | | | | | | | | NRS_expect_  Item 2 |
| **not impaired** | 0 | 1 | 2 | 3 | 4 | 5 | | 6 | 7 | 8 | 9 | 10 | **very strongly impaired** | |
| 2.3 Have you received or self-administered any treatment(s) for your *Back pain* since last evening? | | | | | | | | | | | | | | NRS_treat1_  Item 2 |
| Yes | | | | | | | No | | | | | | | |
| 2.4 If yes, how much did the treatment improve your impairment from the *Back pain*? | | | | | | | | | | | | | | NRS_treat2_  Item 2 |
| **no improvement** | 0 | 1 | 2 | 3 | 4 | 5 | | 6 | 7 | 8 | 9 | 10 | **very strong improvement** | |
| 2.5 How much did the treatment(s) worsen your impairment from the *Back pain*? | | | | | | | | | | | | | | NRS_treat3_  Item 2 |
| **no worsening** | 0 | 1 | 2 | 3 | 4 | 5 | | 6 | 7 | 8 | 9 | 10 | **very strong worsening** | |
| 2.6 How much did you experience adverse side effects from this treatment(s)? | | | | | | | | | | | | | | TreatExp  Item 2 |
| **no side effects** | 0 | 1 | 2 | 3 | 4 | 5 | | 6 | 7 | 8 | 9 | 10 | **very strong side effects** | |
| **3. Pain in your arms, legs, or joints (knees, hips,etc.)** | | | | | | | | | | | | | | PHQ_adapt_  Item 3 |
| **not bothered at all** | 0 | 1 | 2 | 3 | 4 | 5 | | 6 | 7 | 8 | 9 | 10 | **bothered a lot** | |
| 3.2 How impaired do you expect to be by your *Pain in your arms, legs, or joints (knees, hips,etc.)* by midday? | | | | | | | | | | | | | | NRS_expect_  Item 3 |
| **not impaired** | 0 | 1 | 2 | 3 | 4 | 5 | | 6 | 7 | 8 | 9 | 10 | **very strongly impaired** | |
| 3.3 Have you received or self-administered any treatment(s) for your *pain in your arms, legs, or joints (knees, hips,etc.)* since last evening? | | | | | | | | | | | | | | NRS_treat1_  Item 3 |
| Yes | | | | | | | No | | | | | | | |
| 3.4 If yes, how much did the treatment improve your impairment from the *Pain in your arms, legs, or joints (knees, hips,etc.)*? | | | | | | | | | | | | | | NRS_treat2_  Item 3 |
| **no improvement** | 0 | 1 | 2 | 3 | 4 | 5 | | 6 | 7 | 8 | 9 | 10 | **very strong improvement** | |
| 3.5 How much did the treatment(s) worsen your impairment from the *Pain in your arms, legs, or joints (knees, hips,etc.)*? | | | | | | | | | | | | | | NRS_treat3_  Item 3 |
| **no worsening** | 0 | 1 | 2 | 3 | 4 | 5 | | 6 | 7 | 8 | 9 | 10 | **very strong worsening** | |
| 3.6 How much did you experience adverse side effects from this treatment(s)? | | | | | | | | | | | | | | TreatExp  Item 3 |
| **no side effects** | 0 | 1 | 2 | 3 | 4 | 5 | | 6 | 7 | 8 | 9 | 10 | **very strong side effects** | |
| **4. Menstrual cramps or other problems with your periods** | | | | | | | | | | | | | | PHQ_adapt_  Item 4 |
| Does menstruation / period / menstrual bleeding apply to you? | | | | | | | | | | | | | | |
| Yes | | | | | | | No | | | | | | | |
| **not bothered at all** | 0 | 1 | 2 | 3 | 4 | 5 | | 6 | 7 | 8 | 9 | 10 | **bothered a lot** | |
| 4.2 How impaired do you expect to be by your *Menstrual cramps or other problems with your periods* by midday? | | | | | | | | | | | | | | NRS_expect_  Item 4 |
| **not impaired** | 0 | 1 | 2 | 3 | 4 | 5 | | 6 | 7 | 8 | 9 | 10 | **very strongly impaired** | |
| 4.3 Have you received or self-administered any treatment(s) for your *Menstrual cramps or other problems with your periods* since last evening? | | | | | | | | | | | | | | NRS_treat1_  Item 4 |
| Yes | | | | | | | No | | | | | | | |
| 4.4 If yes, how much did the treatment improve your impairment from the *Menstrual cramps or other problems with your periods*? | | | | | | | | | | | | | | NRS_treat2_  Item 4 |
| **no improvement** | 0 | 1 | 2 | 3 | 4 | 5 | | 6 | 7 | 8 | 9 | 10 | **very strong improvement** | |
| 4.5 How much did the treatment(s) worsen your impairment from the *Menstrual cramps or other problems with your periods*? | | | | | | | | | | | | | | NRS_treat3_  Item 4 |
| **no worsening** | 0 | 1 | 2 | 3 | 4 | 5 | | 6 | 7 | 8 | 9 | 10 | **very strong worsening** | |
| 4.6 How much did you experience adverse side effects from this treatment(s)? | | | | | | | | | | | | | | TreatExp  Item 4 |
| **no side effects** | 0 | 1 | 2 | 3 | 4 | 5 | | 6 | 7 | 8 | 9 | 10 | **very strong side effects** | |
| **5. Pain or problems during sexual intercourse** | | | | | | | | | | | | | | PHQ_adapt_  Item 5 |
| **not bothered at all** | 0 | 1 | 2 | 3 | 4 | 5 | | 6 | 7 | 8 | 9 | 10 | **bothered a lot** | |
| 5.2 How impaired do you expect to be by your *Pain or problems during sexual intercourse* by midday? | | | | | | | | | | | | | | NRS_expect_  Item 5 |
| **not impaired** | 0 | 1 | 2 | 3 | 4 | 5 | | 6 | 7 | 8 | 9 | 10 | **very strongly impaired** | |
| 5.3 Have you received or self-administered any treatment(s) for your *Pain or problems during sexual intercourse* since last evening? | | | | | | | | | | | | | | NRS_treat1_  Item 5 |
| Yes | | | | | | | No | | | | | | | |
| 5.4 If yes, how much did the treatment improve your impairment from the *Pain or problems during sexual intercourse*? | | | | | | | | | | | | | | NRS_treat2_  Item 5 |
| **no improvement** | 0 | 1 | 2 | 3 | 4 | 5 | | 6 | 7 | 8 | 9 | 10 | **very strong improvement** | |
| 5.5 How much did the treatment(s) worsen your impairment from the *Pain or problems during sexual intercourse*? | | | | | | | | | | | | | | NRS_treat3_  Item 5 |
| **no worsening** | 0 | 1 | 2 | 3 | 4 | 5 | | 6 | 7 | 8 | 9 | 10 | **very strong worsening** | |
| 5.6 How much did you experience adverse side effects from this treatment(s)? | | | | | | | | | | | | | | TreatExp  Item 5 |
| **no side effects** | 0 | 1 | 2 | 3 | 4 | 5 | | 6 | 7 | 8 | 9 | 10 | **very strong side effects** | |
| **6. Headaches** | | | | | | | | | | | | | | PHQ_adapt_  Item 6 |
| **not bothered at all** | 0 | 1 | 2 | 3 | 4 | 5 | | 6 | 7 | 8 | 9 | 10 | **bothered a lot** | |
| 6.2 How impaired do you expect to be by your *Headaches* by midday? | | | | | | | | | | | | | | NRS_expect_  Item 6 |
| **not impaired** | 0 | 1 | 2 | 3 | 4 | 5 | | 6 | 7 | 8 | 9 | 10 | **very strongly impaired** | |
| 6.3 Have you received or self-administered any treatment(s) for your *Headaches* since last evening? | | | | | | | | | | | | | | NRS_treat1_  Item 6 |
| Yes | | | | | | | No | | | | | | | |
| 6.4 If yes, how much did the treatment improve your impairment from the *Headaches*? | | | | | | | | | | | | | | NRS_treat2_  Item 6 |
| **no improvement** | 0 | 1 | 2 | 3 | 4 | 5 | | 6 | 7 | 8 | 9 | 10 | **very strong improvement** | |
| 6.5 How much did the treatment(s) worsen your impairment from the *Headaches*? | | | | | | | | | | | | | | NRS_treat3_  Item 6 |
| **no worsening** | 0 | 1 | 2 | 3 | 4 | 5 | | 6 | 7 | 8 | 9 | 10 | **very strong worsening** | |
| 6.6 How much did you experience adverse side effects from this treatment(s)? | | | | | | | | | | | | | | TreatExp  Item 6 |
| **no side effects** | 0 | 1 | 2 | 3 | 4 | 5 | | 6 | 7 | 8 | 9 | 10 | **very strong side effects** | |
| **7. Chest pain** | | | | | | | | | | | | | | PHQ_adapt_  Item 7 |
| **not bothered at all** | 0 | 1 | 2 | 3 | 4 | 5 | | 6 | 7 | 8 | 9 | 10 | **bothered a lot** | |
| 7.2 How impaired do you expect to be by your *Chest pain* by midday? | | | | | | | | | | | | | | NRS_expect_  Item 7 |
| **not impaired** | 0 | 1 | 2 | 3 | 4 | 5 | | 6 | 7 | 8 | 9 | 10 | **very strongly impaired** | |
| 7.3 Have you received or self-administered any treatment(s) for your *Chest pain* since last evening? | | | | | | | | | | | | | | NRS_treat1_  Item 7 |
| Yes | | | | | | | No | | | | | | | |
| 7.4 If yes, how much did the treatment improve your impairment from the *Chest pain?* | | | | | | | | | | | | | | NRS_treat2_  Item 7 |
| **no improvement** | 0 | 1 | 2 | 3 | 4 | 5 | | 6 | 7 | 8 | 9 | 10 | **very strong improvement** | |
| 7.5 How much did the treatment(s) worsen your impairment from the *Chest pain*? | | | | | | | | | | | | | | NRS_treat3_  Item 7 |
| **no worsening** | 0 | 1 | 2 | 3 | 4 | 5 | | 6 | 7 | 8 | 9 | 10 | **very strong worsening** | |
| 7.6 How much did you experience adverse side effects from this treatment(s)? | | | | | | | | | | | | | | TreatExp  Item 7 |
| **no side effects** | 0 | 1 | 2 | 3 | 4 | 5 | | 6 | 7 | 8 | 9 | 10 | **very strong side effects** | |
| **8. Dizziness** | | | | | | | | | | | | | | PHQ_adapt_  Item 8 |
| **not bothered at all** | 0 | 1 | 2 | 3 | 4 | 5 | | 6 | 7 | 8 | 9 | 10 | **bothered a lot** | |
| 8.2 How impaired do you expect to be by your *Dizziness* by midday? | | | | | | | | | | | | | | NRS_expect_  Item 8 |
| **not impaired** | 0 | 1 | 2 | 3 | 4 | 5 | | 6 | 7 | 8 | 9 | 10 | **very strongly impaired** | |
| 8.3 Have you received or self-administered any treatment(s) for your *Dizziness* since last evening? | | | | | | | | | | | | | | NRS_treat1_  Item 8 |
| Yes | | | | | | | No | | | | | | | |
| 8.4 If yes, how much did the treatment improve your impairment from the *Dizziness*? | | | | | | | | | | | | | | NRS_treat2_  Item 8 |
| **no improvement** | 0 | 1 | 2 | 3 | 4 | 5 | | 6 | 7 | 8 | 9 | 10 | **very strong improvement** | |
| 8.5 How much did the treatment(s) worsen your impairment from the *Dizziness*? | | | | | | | | | | | | | | NRS_treat3_  Item 8 |
| **no worsening** | 0 | 1 | 2 | 3 | 4 | 5 | | 6 | 7 | 8 | 9 | 10 | **very strong worsening** | |
| 8.6 How much did you experience adverse side effects from this treatment(s)? | | | | | | | | | | | | | | TreatExp  Item 8 |
| **no side effects** | 0 | 1 | 2 | 3 | 4 | 5 | | 6 | 7 | 8 | 9 | 10 | **very strong side effects** | |
| **9. Fainting spells** | | | | | | | | | | | | | | PHQ_adapt_  Item 9 |
| **not bothered at all** | 0 | 1 | 2 | 3 | 4 | 5 | | 6 | 7 | 8 | 9 | 10 | **bothered a lot** | |
| 9.2 How impaired do you expect to be by your *Fainting spells* by midday? | | | | | | | | | | | | | | NRS_expect_  Item 9 |
| **not impaired** | 0 | 1 | 2 | 3 | 4 | 5 | | 6 | 7 | 8 | 9 | 10 | **very strongly impaired** | |
| 9.3 Have you received or self-administered any treatment(s) for your *Fainting spells* since last evening? | | | | | | | | | | | | | | NRS_treat1_  Item 9 |
| Yes | | | | | | | No | | | | | | | |
| 9.4 If yes, how much did the treatment improve your impairment from the *Fainting spells?* | | | | | | | | | | | | | | NRS_treat2_  Item 9 |
| **no improvement** | 0 | 1 | 2 | 3 | 4 | 5 | | 6 | 7 | 8 | 9 | 10 | **very strong improvement** | |
| 9.5 How much did the treatment(s) worsen your impairment from the *Fainting spells*? | | | | | | | | | | | | | | NRS_treat3_  Item 9 |
| **no worsening** | 0 | 1 | 2 | 3 | 4 | 5 | | 6 | 7 | 8 | 9 | 10 | **very strong worsening** | |
| 9.6 How much did you experience adverse side effects from this treatment(s)? | | | | | | | | | | | | | | TreatExp  Item 9 |
| **no side effects** | 0 | 1 | 2 | 3 | 4 | 5 | | 6 | 7 | 8 | 9 | 10 | **very strong side effects** | |
| **10. Feeling your heart pound or race** | | | | | | | | | | | | | | PHQ_adapt_  Item 10 |
| **not bothered at all** | 0 | 1 | 2 | 3 | 4 | 5 | | 6 | 7 | 8 | 9 | 10 | **bothered a lot** | |
| 10.2 How impaired do you expect to be by your *Feeling your heart pound or race* by midday? | | | | | | | | | | | | | | NRS_expect_  Item 10 |
| **not impaired** | 0 | 1 | 2 | 3 | 4 | 5 | | 6 | 7 | 8 | 9 | 10 | **very strongly impaired** | |
| 10.3 Have you received or self-administered any treatment(s) for your *Feeling your heart pound or race* since last evening? | | | | | | | | | | | | | | NRS_treat1_  Item 10 |
| Yes | | | | | | | No | | | | | | | |
| 10.4 If yes, how much did the treatment improve your impairment from the *Feeling your heart pound or race*? | | | | | | | | | | | | | | NRS_treat2_  Item 10 |
| **no improvement** | 0 | 1 | 2 | 3 | 4 | 5 | | 6 | 7 | 8 | 9 | 10 | **very strong improvement** | |
| 10.5 How much did the treatment(s) worsen your impairment from the *Feeling your heart pound or race*? | | | | | | | | | | | | | | NRS_treat3_  Item 10 |
| **no worsening** | 0 | 1 | 2 | 3 | 4 | 5 | | 6 | 7 | 8 | 9 | 10 | **very strong worsening** | |
| 10.6 How much did you experience adverse side effects from this treatment(s)? | | | | | | | | | | | | | | TreatExp  Item 10 |
| **no side effects** | 0 | 1 | 2 | 3 | 4 | 5 | | 6 | 7 | 8 | 9 | 10 | **very strong side effects** | |
| **11. Shortness of breath** | | | | | | | | | | | | | | PHQ_adapt_  Item 11 |
| **not bothered at all** | 0 | 1 | 2 | 3 | 4 | 5 | | 6 | 7 | 8 | 9 | 10 | **bothered a lot** | |
| 11.2 How impaired do you expect to be by your *Shortness of breath* by midday? | | | | | | | | | | | | | | NRS_expect_  Item 11 |
| **not impaired** | 0 | 1 | 2 | 3 | 4 | 5 | | 6 | 7 | 8 | 9 | 10 | **very strongly impaired** | |
| 11.3 Have you received or self-administered any treatment(s) for your *Shortness of breath* since last evening? | | | | | | | | | | | | | | NRS_treat1_  Item 11 |
| Yes | | | | | | | No | | | | | | | |
| 11.4 If yes, how much did the treatment improve your impairment from the *Shortness of breath*? | | | | | | | | | | | | | | NRS_treat2_  Item 11 |
| **no improvement** | 0 | 1 | 2 | 3 | 4 | 5 | | 6 | 7 | 8 | 9 | 10 | **very strong improvement** | |
| 11.5 How much did the treatment(s) worsen your impairment from the *Shortness of breath*? | | | | | | | | | | | | | | NRS_treat3_  Item 11 |
| **no worsening** | 0 | 1 | 2 | 3 | 4 | 5 | | 6 | 7 | 8 | 9 | 10 | **very strong worsening** | |
| 11.6 How much did you experience adverse side effects from this treatment(s)? | | | | | | | | | | | | | | TreatExp  Item 11 |
| **no side effects** | 0 | 1 | 2 | 3 | 4 | 5 | | 6 | 7 | 8 | 9 | 10 | **very strong side effects** | |
| **12. Constipation, loose bowels, or diarrhea** | | | | | | | | | | | | | | PHQ_adapt_  Item 12 |
| **not bothered at all** | 0 | 1 | 2 | 3 | 4 | 5 | | 6 | 7 | 8 | 9 | 10 | **bothered a lot** | |
| 12.2 How impaired do you expect to be by your *Constipation, loose bowels, or diarrhea* by midday? | | | | | | | | | | | | | | NRS_expect_  Item 12 |
| **not impaired** | 0 | 1 | 2 | 3 | 4 | 5 | | 6 | 7 | 8 | 9 | 10 | **very strongly impaired** | |
| 12. Have you received or self-administered any treatment(s) for your *Constipation, loose bowels, or diarrhea* since last evening? | | | | | | | | | | | | | | NRS_treat1_  Item 12 |
| Yes | | | | | | | No | | | | | | | |
| 12.4 If yes, how much did the treatment improve your impairment from the *Constipation, loose bowels, or diarrhea*? | | | | | | | | | | | | | | NRS_treat2_  Item 12 |
| **no improvement** | 0 | 1 | 2 | 3 | 4 | 5 | | 6 | 7 | 8 | 9 | 10 | **very strong improvement** | |
| 12.5 How much did the treatment(s) worsen your impairment from the *Constipation, loose bowels, or diarrhea*? | | | | | | | | | | | | | | NRS_treat3_  Item 12 |
| **no worsening** | 0 | 1 | 2 | 3 | 4 | 5 | | 6 | 7 | 8 | 9 | 10 | **very strong worsening** | |
| 12.6 How much did you experience adverse side effects from this treatment(s)? | | | | | | | | | | | | | | TreatExp  Item 12 |
| **no side effects** | 0 | 1 | 2 | 3 | 4 | 5 | | 6 | 7 | 8 | 9 | 10 | **very strong side effects** | |
| **13. Nausea, gas, or indigestion** | | | | | | | | | | | | | | PHQ_adapt_  Item 13 |
| **not bothered at all** | 0 | 1 | 2 | 3 | 4 | 5 | | 6 | 7 | 8 | 9 | 10 | **bothered a lot** | |
| 13.2 How impaired do you expect to be by your *Nausea, gas, or indigestion* by midday? | | | | | | | | | | | | | | NRS_expect_  Item 13 |
| **not impaired** | 0 | 1 | 2 | 3 | 4 | 5 | | 6 | 7 | 8 | 9 | 10 | **very strongly impaired** | |
| 13.3 Have you received or self-administered any treatment(s) for your *Nausea, gas, or indigestion* since last evening? | | | | | | | | | | | | | | NRS_treat1_  Item 13 |
| Yes | | | | | | | No | | | | | | | |
| 13.4 If yes, how much did the treatment improve your impairment from the *Nausea, gas, or indigestion*? | | | | | | | | | | | | | | NRS_treat2_  Item 13 |
| **no improvement** | 0 | 1 | 2 | 3 | 4 | 5 | | 6 | 7 | 8 | 9 | 10 | **very strong improvement** | |
| 13.5 How much did the treatment(s) worsen your impairment from the *Nausea, gas, or indigestion*? | | | | | | | | | | | | | | NRS_treat3_  Item 13 |
| **no worsening** | 0 | 1 | 2 | 3 | 4 | 5 | | 6 | 7 | 8 | 9 | 10 | **very strong worsening** | |
| 13.6 How much did you experience adverse side effects from this treatment(s)? | | | | | | | | | | | | | | TreatExp  Item 13 |
| **no side effects** | 0 | 1 | 2 | 3 | 4 | 5 | | 6 | 7 | 8 | 9 | 10 | **very strong side effects** | |
| **14. Feeling tired or having low energy** | | | | | | | | | | | | | | PHQ_adapt_  Item 14 |
| **not bothered at all** | 0 | 1 | 2 | 3 | 4 | 5 | | 6 | 7 | 8 | 9 | 10 | **bothered a lot** | |
| 14.2 How impaired do you expect to be by your *Feeling tired or having low energy* by midday? | | | | | | | | | | | | | | NRS_expect_  Item 14 |
| **not impaired** | 0 | 1 | 2 | 3 | 4 | 5 | | 6 | 7 | 8 | 9 | 10 | **very strongly impaired** | |
| 14.3 Have you received or self-administered any treatment(s) for your *Feeling tired or having low energy* since last evening? | | | | | | | | | | | | | | NRS_treat1_  Item 14 |
| Yes | | | | | | | No | | | | | | | |
| 14.4 If yes, how much did the treatment improve your impairment from the *Feeling tired or having low energy*? | | | | | | | | | | | | | | NRS_treat2_  Item 14 |
| **no improvement** | 0 | 1 | 2 | 3 | 4 | 5 | | 6 | 7 | 8 | 9 | 10 | **very strong improvement** | |
| 14.5 How much did the treatment(s) worsen your impairment from the *Feeling tired or having low energy*? | | | | | | | | | | | | | | NRS_treat3_  Item 14 |
| **no worsening** | 0 | 1 | 2 | 3 | 4 | 5 | | 6 | 7 | 8 | 9 | 10 | **very strong worsening** | |
| 14.6 How much did you experience adverse side effects from this treatment(s)? | | | | | | | | | | | | | | TreatExp  Item 14 |
| **no side effects** | 0 | 1 | 2 | 3 | 4 | 5 | | 6 | 7 | 8 | 9 | 10 | **very strong side effects** | |
| **15. Trouble sleeping** | | | | | | | | | | | | | | PHQ_adapt_  Item 15 |
| **not bothered at all** | 0 | 1 | 2 | 3 | 4 | 5 | | 6 | 7 | 8 | 9 | 10 | **bothered a lot** | |
| 15.2 How impaired do you expect to be by your *Trouble sleeping* by midday? | | | | | | | | | | | | | | NRS_expect_  Item 15 |
| **not impaired** | 0 | 1 | 2 | 3 | 4 | 5 | | 6 | 7 | 8 | 9 | 10 | **very strongly impaired** | |
| 15.3 Have you received or self-administered any treatment(s) for your *Trouble sleeping* since last evening? | | | | | | | | | | | | | | NRS_treat1_  Item 15 |
| Yes | | | | | | | No | | | | | | | |
| 15.4 If yes, how much did the treatment improve your impairment from the *Trouble sleeping*? | | | | | | | | | | | | | | NRS_treat2_  Item 15 |
| **no improvement** | 0 | 1 | 2 | 3 | 4 | 5 | | 6 | 7 | 8 | 9 | 10 | **very strong improvement** | |
| 15.5 How much did the treatment(s) worsen your impairment from the *Trouble sleeping*? | | | | | | | | | | | | | | NRS_treat3_  Item 15 |
| **no worsening** | 0 | 1 | 2 | 3 | 4 | 5 | | 6 | 7 | 8 | 9 | 10 | **very strong worsening** | |
| 15.6 How much did you experience adverse side effects from this treatment(s)? | | | | | | | | | | | | | | TreatExp  Item 15 |
| **no side effects** | 0 | 1 | 2 | 3 | 4 | 5 | | 6 | 7 | 8 | 9 | 10 | **very strong side effects** | |

**Complete:** *You're all done! We want to thank you for answering these questions and ask you to continue to support us in the next survey at midday. Thank you!*

**English Translation: Adapted Questionnaire (PHQ_adapt_): Positive Expectation Framing. Morning Assessment**

**Ecological Momentary Assessment (EMA)**

**Framing-Group:** Positive

**Online-survey: Morning**

**Introduction: *“****Welcome to this online survey of our study. Thank you for taking the time to participate this morning.”*

| The following section focuses on assessing which physical symptoms are affecting you right now, what expectations you have regarding the impairment due to these symptoms, and whether you have already taken any steps to address them. | | | | | | | | | | | | | | | | | | | | | | | | | |
| --- | --- | --- | --- | --- | --- | --- | --- | --- | --- | --- | --- | --- | --- | --- | --- | --- | --- | --- | --- | --- | --- | --- | --- | --- | --- |
| **At the moment, how much are you bothered by the following symptoms?** | | | | | | | | | | | | | | | | | | | | | | | | | |
| **1. Stomach pain** | | | | | | | | | | | | | | | | | | | | | | | PHQ_adapt_  Item 1 | | |
| **not bothered at all** | 0 | 1 | 2 | 3 | 4 | | 5 | | | | 6 | | 7 | | 8 | | 9 | | 10 | | **bothered a lot** | | | | |
| 1.2 How free of impairment do you expect to be from S*tomach pain* by midday? | | | | | | | | | | | | | | | | | | | | | | | NRS_expect#_  Item 1 | | |
| **not free from impairment** | 0 | 1 | 2 | 3 | 4 | | 5 | | | | 6 | | 7 | | 8 | | 9 | | 10 | | **very free from impairment** | | | | |
| 1.3 Have you received or self-administered any treatment(s) for your *Stomach pain* since last evening? | | | | | | | | | | | | | | | | | | | | | | | NRS_treat1_  Item 1 | | |
| Yes | | | | | | | | | No | | | | | | | | | | | | | | | | |
| 1.4 If yes, how much did the treatment improve your impairment from the *Stomach pain?* | | | | | | | | | | | | | | | | | | | | | | | NRS_treat2_  Item 1 | | |
| **no improvement** | 0 | 1 | 2 | 3 | 4 | | 5 | | | | 6 | | 7 | | 8 | | 9 | | 10 | | **very strong improvement** | | | | |
| 1.5 How much did the treatment(s) worsen your impairment from the *Stomach pain*? | | | | | | | | | | | | | | | | | | | | | | | NRS_treat3_  Item 1 | | |
| **no worsening** | 0 | 1 | 2 | 3 | 4 | | 5 | | | | 6 | | 7 | | 8 | | 9 | | 10 | | **very strong worsening** | | | | |
| 1.6 How much did you experience adverse side effects from this treatment(s)? | | | | | | | | | | | | | | | | | | | | | | | TreatExp  Item 1 | | |
| **no side effects** | 0 | 1 | 2 | 3 | 4 | | 5 | | | | 6 | | 7 | | 8 | | 9 | | 10 | | **very strong side effects** | | | | |
| **2. Back pain** | | | | | | | | | | | | | | | | | | | | | | | PHQ_adapt_  Item 2 | | |
| **not bothered at all** | 0 | 1 | 2 | 3 | 4 | | 5 | | | | 6 | | 7 | | 8 | | 9 | | 10 | | **bothered a lot** | | | | |
| 2.2 How free of impairment do you expect to be from *Back pain* by midday? | | | | | | | | | | | | | | | | | | | | | | | NRS_expect#_  Item 2 | | |
| **not free from impairment** | 0 | 1 | 2 | 3 | 4 | | 5 | | | | 6 | | 7 | | 8 | | 9 | | 10 | | **very free from impairment** | | | | |
| 2.3 Have you received or self-administered any treatment(s) for your *Back pain* since last evening? | | | | | | | | | | | | | | | | | | | | | | | NRS_treat1_  Item 2 | | |
| Yes | | | | | | | | | No | | | | | | | | | | | | | | | | |
| 2.4 If yes, how much did the treatment improve your impairment from the *Back pain*? | | | | | | | | | | | | | | | | | | | | | | | NRS_treat2_  Item 2 | | |
| **no improvement** | 0 | 1 | 2 | 3 | 4 | | 5 | | | | 6 | | 7 | | 8 | | 9 | | 10 | | **very strong improvement** | | | | |
| 2.5 How much did the treatment(s) worsen your impairment from the *Back pain*? | | | | | | | | | | | | | | | | | | | | | | | NRS_treat3_  Item 2 | | |
| **no worsening** | 0 | 1 | 2 | 3 | 4 | | 5 | | | | 6 | | 7 | | 8 | | 9 | | 10 | | **very strong worsening** | | | | |
| 2.6 How much did you experience adverse side effects from this treatment(s)? | | | | | | | | | | | | | | | | | | | | | | | TreatExp  Item 2 | | |
| **no side effects** | 0 | 1 | 2 | 3 | 4 | | 5 | | | | 6 | | 7 | | 8 | | 9 | | 10 | | **very strong side effects** | | | | |
| **3. Pain in your arms, legs, or joints (knees, hips,etc.)** | | | | | | | | | | | | | | | | | | | | | | | PHQ_adapt_  Item 3 | | |
| **not bothered at all** | 0 | 1 | 2 | 3 | 4 | | 5 | | | | 6 | | 7 | | 8 | | 9 | | 10 | | **bothered a lot** | | | | |
| 3.2 How free of impairment do you expect to be from *Pain in your arms, legs, or joints (knees, hips,etc.* by midday? | | | | | | | | | | | | | | | | | | | | | | | NRS_expect#_  Item 3 | | |
| **not free from impairment** | 0 | 1 | 2 | 3 | 4 | | 5 | | | | 6 | | 7 | | 8 | | 9 | | 10 | | **very free from impairment** | | | | |
| 3.3 Have you received or self-administered any treatment(s) for your *Pain in your arms, legs, or joints (knees, hips,etc.)* since last evening? | | | | | | | | | | | | | | | | | | | | | | | NRS_treat1_  Item 3 | | |
| Yes | | | | | | | | | No | | | | | | | | | | | | | | | | |
| 3.4 If yes, how much did the treatment improve your impairment from the *Pain in your arms, legs, or joints (knees, hips,etc.)*? | | | | | | | | | | | | | | | | | | | | | | | NRS_treat2_  Item 3 | | |
| **no improvement** | 0 | 1 | 2 | 3 | 4 | | 5 | | | | 6 | | 7 | | 8 | | 9 | | 10 | | **very strong improvement** | | | | |
| 3.5 How much did the treatment(s) worsen your impairment from the *Pain in your arms, legs, or joints (knees, hips,etc.)*? | | | | | | | | | | | | | | | | | | | | | | | NRS_treat3_  Item 3 | | |
| **no worsening** | 0 | 1 | 2 | 3 | 4 | | 5 | | | | 6 | | 7 | | 8 | | 9 | | 10 | | **very strong worsening** | | | | |
| 3.6 How much did you experience adverse side effects from this treatment(s)? | | | | | | | | | | | | | | | | | | | | | | | TreatExp  Item 3 | | |
| **no side effects** | 0 | 1 | 2 | 3 | 4 | | 5 | | | | 6 | | 7 | | 8 | | 9 | | 10 | | **very strong side effects** | | | | |
| **4. Menstrual cramps or other problems with your periods** | | | | | | | | | | | | | | | | | | | | | | | PHQ_adapt_  Item 4 | | |
| Does menstruation / period / menstrual bleeding apply to you? | | | | | | | | | | | | | | | | | | | | | | | | | |
| Yes | | | | | | | | | | No | | | | | | | | | | | | | | | |
| **not bothered at all** | 0 | 1 | 2 | 3 | 4 | | 5 | | | | 6 | | 7 | | 8 | | 9 | | 10 | | **bothered a lot** | | | | |
| 4.2 How free of impairment do you expect to be from *Menstrual cramps or other problems with your periods* by midday? | | | | | | | | | | | | | | | | | | | | | | | NRS_expect#_  Item 4 | | |
| **not free from impairment** | 0 | 1 | 2 | 3 | 4 | | 5 | | | | 6 | | 7 | | 8 | | 9 | | 10 | | **very free from impairment** | | | | |
| 4.3 Have you received or self-administered any treatment(s) for your *Menstrual cramps or other problems with your periods* since last evening? | | | | | | | | | | | | | | | | | | | | | | | NRS_treat1_  Item 4 | | |
| Yes | | | | | | | | | No | | | | | | | | | | | | | | | | |
| 4.4 If yes, how much did the treatment improve your impairment from the *Menstrual cramps or other problems with your periods*? | | | | | | | | | | | | | | | | | | | | | | | NRS_treat2_  Item 4 | | |
| **no improvement** | 0 | 1 | 2 | 3 | 4 | | 5 | | | | 6 | | 7 | | 8 | | 9 | | 10 | | **very strong improvement** | | | | |
| 4.5 How much did the treatment(s) worsen your impairment from the *Menstrual cramps or other problems with your periods*? | | | | | | | | | | | | | | | | | | | | | | | NRS_treat3_  Item 4 | | |
| **no worsening** | 0 | 1 | 2 | 3 | 4 | | 5 | | | | 6 | | 7 | | 8 | | 9 | | 10 | | **very strong worsening** | | | | |
| 4.6 How much did you experience adverse side effects from this treatment(s)? | | | | | | | | | | | | | | | | | | | | | | | TreatExp  Item 4 | | |
| **no side effects** | 0 | 1 | 2 | 3 | 4 | | 5 | | | | 6 | | 7 | | 8 | | 9 | | 10 | | **very strong side effects** | | | | |
| **5. Pain or problems during sexual intercourse** | | | | | | | | | | | | | | | | | | | | | | | PHQ_adapt_  Item 5 | | |
| **not bothered at all** | 0 | 1 | 2 | 3 | 4 | | 5 | | | | 6 | | 7 | | 8 | | 9 | | 10 | | **bothered a lot** | | | | |
| 5.2 How free of impairment do you expect to be from *Pain or problems during sexual intercourse* by midday? | | | | | | | | | | | | | | | | | | | | | | | NRS_expect#_  Item 5 | | |
| **not free from impairment** | 0 | 1 | 2 | 3 | 4 | | 5 | | | | 6 | | 7 | | 8 | | 9 | | 10 | | **very free from impairment** | | | | |
| 5.3 Have you received or self-administered any treatment(s) for your *Pain or problems during sexual intercourse* since last evening? | | | | | | | | | | | | | | | | | | | | | | | NRS_treat1_  Item 5 | | |
| Yes | | | | | | | | | No | | | | | | | | | | | | | | | | |
| 5.4 If yes, how much did the treatment improve your impairment from the *Pain or problems during sexual intercourse*? | | | | | | | | | | | | | | | | | | | | | | | NRS_treat2_  Item 5 | | |
| **no improvement** | 0 | 1 | 2 | 3 | 4 | | 5 | | | | 6 | | 7 | | 8 | | 9 | | 10 | | **very strong improvement** | | | | |
| 5.5 How much did the treatment(s) worsen your impairment from the *Pain or problems during sexual intercourse*? | | | | | | | | | | | | | | | | | | | | | | | NRS_treat3_  Item 5 | | |
| **no worsening** | 0 | 1 | 2 | 3 | 4 | | 5 | | | | 6 | | 7 | | 8 | | 9 | | 10 | | **very strong worsening** | | | | |
| 5.6 How much did you experience adverse side effects from this treatment(s)? | | | | | | | | | | | | | | | | | | | | | | | TreatExp  Item 5 | | |
| **no side effects** | 0 | 1 | 2 | 3 | 4 | | 5 | | | | 6 | | 7 | | 8 | | 9 | | 10 | | **very strong side effects** | | | | |
| **6. Headaches** | | | | | | | | | | | | | | | | | | | | | | | PHQ_adapt_  Item 6 | | |
| **not bothered at all** | 0 | 1 | 2 | 3 | 4 | | 5 | | | | 6 | | 7 | | 8 | | 9 | | 10 | | **bothered a lot** | | | | |
| 6.2 How free of impairment do you expect to be from *Headaches* by midday? | | | | | | | | | | | | | | | | | | | | | | | NRS_expect#_  Item 6 | | |
| **not free from impairment** | 0 | 1 | 2 | 3 | 4 | | 5 | | | | 6 | | 7 | | 8 | | 9 | | 10 | | **very free from impairment** | | | | |
| 6.3 Have you received or self-administered any treatment(s) for your *Headaches* since last evening? | | | | | | | | | | | | | | | | | | | | | | | NRS_treat1_  Item 6 | | |
| Yes | | | | | | | | | No | | | | | | | | | | | | | | | | |
| 6.4 If yes, how much did the treatment improve your impairment from the *Headaches*? | | | | | | | | | | | | | | | | | | | | | | | NRS_treat2_  Item 6 | | |
| **no improvement** | 0 | 1 | 2 | 3 | 4 | | 5 | | | | 6 | | 7 | | 8 | | 9 | | 10 | | **very strong improvement** | | | | |
| 6.5 How much did the treatment(s) worsen your impairment from the *Headaches*? | | | | | | | | | | | | | | | | | | | | | | | NRS_treat3_  Item 6 | | |
| **no worsening** | 0 | 1 | 2 | 3 | 4 | | 5 | | | | 6 | | 7 | | 8 | | 9 | | 10 | | **very strong worsening** | | | | |
| 6.6 How much did you experience adverse side effects from this treatment(s)? | | | | | | | | | | | | | | | | | | | | | | | | TreatExp  Item 6 |  |
| **no side effects** | 0 | 1 | 2 | 3 | | 4 | | 5 | | | | 6 | | 7 | | 8 | | 9 | | 10 | | **very strong side effects** | | |  |
| **7. Chest pain** | | | | | | | | | | | | | | | | | | | | | | | | PHQ_adapt_  Item 7 |  |
| **not bothered at all** | 0 | 1 | 2 | 3 | | 4 | | 5 | | | | 6 | | 7 | | 8 | | 9 | | 10 | | **bothered a lot** | | |  |
| 7.2 How free of impairment do you expect to be from *Chest pain* by midday? | | | | | | | | | | | | | | | | | | | | | | | | NRS_expect#_  Item 7 |  |
| **not free from impairment** | 0 | 1 | 2 | 3 | | 4 | | 5 | | | | 6 | | 7 | | 8 | | 9 | | 10 | | **very free from impairment** | | |  |
| 7.3 Have you received or self-administered any treatment(s) for your *Chest pain* since last evening? | | | | | | | | | | | | | | | | | | | | | | | | NRS_treat1_  Item 7 |  |
| Yes | | | | | | | | | No | | | | | | | | | | | | | | | |  |
| 7.4 If yes, how much did the treatment improve your impairment from the *Chest pain?* | | | | | | | | | | | | | | | | | | | | | | | | NRS_treat2_  Item 7 |  |
| **no improvement** | 0 | 1 | 2 | 3 | | 4 | | 5 | | | | 6 | | 7 | | 8 | | 9 | | 10 | | **very strong improvement** | | |  |
| 7.5 How much did the treatment(s) worsen your impairment from the *Chest pain*? | | | | | | | | | | | | | | | | | | | | | | | | NRS_treat3_  Item 7 |  |
| **no worsening** | 0 | 1 | 2 | 3 | | 4 | | 5 | | | | 6 | | 7 | | 8 | | 9 | | 10 | | **very strong worsening** | | |  |
| 7.6 How much did you experience adverse side effects from this treatment(s)? | | | | | | | | | | | | | | | | | | | | | | | | TreatExp  Item 7 |  |
| **no side effects** | 0 | 1 | 2 | 3 | | 4 | | 5 | | | | 6 | | 7 | | 8 | | 9 | | 10 | | **very strong side effects** | | |  |
| **8. Dizziness** | | | | | | | | | | | | | | | | | | | | | | | | PHQ_adapt_  Item 8 |  |
| **not bothered at all** | 0 | 1 | 2 | 3 | | 4 | | 5 | | | | 6 | | 7 | | 8 | | 9 | | 10 | | **bothered a lot** | | |  |
| 8.2 How free of impairment do you expect to be from *Dizziness* by midday? | | | | | | | | | | | | | | | | | | | | | | | | NRS_expect#_  Item 8 |  |
| **not free from impairment** | 0 | 1 | 2 | 3 | | 4 | | 5 | | | | 6 | | 7 | | 8 | | 9 | | 10 | | **very free from impairment** | | |  |
| 8.3 Have you received or self-administered any treatment(s) for your *Dizziness* since last evening? | | | | | | | | | | | | | | | | | | | | | | | | NRS_treat1_  Item 8 |  |
| Yes | | | | | | | | | No | | | | | | | | | | | | | | | |  |
| 8.4 If yes, how much did the treatment improve your impairment from the *Dizziness*? | | | | | | | | | | | | | | | | | | | | | | | | NRS_treat2_  Item 8 |  |
| **no improvement** | 0 | 1 | 2 | 3 | | 4 | | 5 | | | | 6 | | 7 | | 8 | | 9 | | 10 | | **very strong improvement** | | |  |
| 8.5 How much did the treatment(s) worsen your impairment from the *Dizziness*? | | | | | | | | | | | | | | | | | | | | | | | | NRS_treat3_  Item 8 |  |
| **no worsening** | 0 | 1 | 2 | 3 | | 4 | | 5 | | | | 6 | | 7 | | 8 | | 9 | | 10 | | **very strong worsening** | | |  |
| 8.6 How much did you experience adverse side effects from this treatment(s)? | | | | | | | | | | | | | | | | | | | | | | | | TreatExp  Item 8 |  |
| **no side effects** | 0 | 1 | 2 | 3 | | 4 | | 5 | | | | 6 | | 7 | | 8 | | 9 | | 10 | | **very strong side effects** | | |  |
| **9. Fainting spells** | | | | | | | | | | | | | | | | | | | | | | | | PHQ_adapt_  Item 9 |  |
| **not bothered at all** | 0 | 1 | 2 | 3 | | 4 | | 5 | | | | 6 | | 7 | | 8 | | 9 | | 10 | | **bothered a lot** | | |  |
| 9.2 How free of impairment do you expect to be from *Fainting spells* by midday? | | | | | | | | | | | | | | | | | | | | | | | | NRS_expect#_  Item 9 |  |
| **not free from impairment** | 0 | 1 | 2 | 3 | | 4 | | 5 | | | | 6 | | 7 | | 8 | | 9 | | 10 | | **very free from impairment** | | |  |
| 9.3 Have you received or self-administered any treatment(s) for your *Fainting spells* since last evening? | | | | | | | | | | | | | | | | | | | | | | | | NRS_treat1_  Item 9 |  |
| Yes | | | | | | | | | No | | | | | | | | | | | | | | | |  |
| 9.4 If yes, how much did the treatment improve your impairment from the *Fainting spells?* | | | | | | | | | | | | | | | | | | | | | | | | NRS_treat2_  Item 9 |  |
| **no improvement** | 0 | 1 | 2 | 3 | | 4 | | 5 | | | | 6 | | 7 | | 8 | | 9 | | 10 | | **very strong improvement** | | |  |
| 9.5 How much did the treatment(s) worsen your impairment from the *Fainting spells*? | | | | | | | | | | | | | | | | | | | | | | | | NRS_treat3_  Item 9 |  |
| **no worsening** | 0 | 1 | 2 | 3 | | 4 | | 5 | | | | 6 | | 7 | | 8 | | 9 | | 10 | | **very strong worsening** | | |  |
| 9.6 How much did you experience adverse side effects from this treatment(s)? | | | | | | | | | | | | | | | | | | | | | | | | TreatExp  Item 9 |  |
| **no side effects** | 0 | 1 | 2 | 3 | | 4 | | 5 | | | | 6 | | 7 | | 8 | | 9 | | 10 | | **very strong side effects** | | |  |
| **10. Feeling your heart pound or race** | | | | | | | | | | | | | | | | | | | | | | | | PHQ_adapt_  Item 10 |  |
| **not bothered at all** | 0 | 1 | 2 | 3 | | 4 | | 5 | | | | 6 | | 7 | | 8 | | 9 | | 10 | | **bothered a lot** | | |  |
| 10.2 How free of impairment do you expect to be from *Feeling your heart pound or race* by midday? | | | | | | | | | | | | | | | | | | | | | | | | NRS_expect#_  Item 10 |  |
| **not free from impairment** | 0 | 1 | 2 | 3 | | 4 | | 5 | | | | 6 | | 7 | | 8 | | 9 | | 10 | | **very free from impairment** | | |  |
| 10.3 Have you received or self-administered any treatment(s) for your *Feeling your heart pound or race* since last evening? | | | | | | | | | | | | | | | | | | | | | | | | NRS_treat1_  Item 10 |  |
| Yes | | | | | | | | | No | | | | | | | | | | | | | | | |  |
| 10.4 If yes, how much did the treatment improve your impairment from the *Feeling your heart pound or race*? | | | | | | | | | | | | | | | | | | | | | | | | NRS_treat2_  Item 10 |  |
| **no improvement** | 0 | 1 | 2 | 3 | | 4 | | 5 | | | | 6 | | 7 | | 8 | | 9 | | 10 | | **very strong improvement** | | |  |
| 10.5 How much did the treatment(s) worsen your impairment from the *Feeling your heart pound or race*? | | | | | | | | | | | | | | | | | | | | | | | | NRS_treat3_  Item 10 |  |
| **no worsening** | 0 | 1 | 2 | 3 | | 4 | | 5 | | | | 6 | | 7 | | 8 | | 9 | | 10 | | **very strong worsening** | | |  |
| 10.6 How much did you experience adverse side effects from this treatment(s)? | | | | | | | | | | | | | | | | | | | | | | | | TreatExp  Item 10 |  |
| **no side effects** | 0 | 1 | 2 | 3 | | 4 | | 5 | | | | 6 | | 7 | | 8 | | 9 | | 10 | | **very strong side effects** | | |  |
| **11. Shortness of breath** | | | | | | | | | | | | | | | | | | | | | | | | PHQ_adapt_  Item 11 |  |
| **not bothered at all** | 0 | 1 | 2 | 3 | | 4 | | 5 | | | | 6 | | 7 | | 8 | | 9 | | 10 | | **bothered a lot** | | |  |
| 11.2 How free of impairment do you expect to be from *Shortness of breath* by midday? | | | | | | | | | | | | | | | | | | | | | | | | NRS_expect#_  Item 11 |  |
| **not free from impairment** | 0 | 1 | 2 | 3 | | 4 | | 5 | | | | 6 | | 7 | | 8 | | 9 | | 10 | | **very free from impairment** | | |  |
| 11.3 Have you received or self-administered any treatment(s) for your *Shortness of breath* since last evening? | | | | | | | | | | | | | | | | | | | | | | | | NRS_treat1_  Item 11 |  |
| Yes | | | | | | | | | No | | | | | | | | | | | | | | | |  |
| 11.4 If yes, how much did the treatment improve your impairment from the *Shortness of breath*? | | | | | | | | | | | | | | | | | | | | | | | | NRS_treat2_  Item 11 |  |
| **no improvement** | 0 | 1 | 2 | 3 | | 4 | | 5 | | | | 6 | | 7 | | 8 | | 9 | | 10 | | **very strong improvement** | | |  |
| 11.5 How much did the treatment(s) worsen your impairment from the *Shortness of breath*? | | | | | | | | | | | | | | | | | | | | | | | | NRS_treat3_  Item 11 |  |
| **no worsening** | 0 | 1 | 2 | 3 | | 4 | | 5 | | | | 6 | | 7 | | 8 | | 9 | | 10 | | **very strong worsening** | | |  |
| 11.6 How much did you experience adverse side effects from this treatment(s)? | | | | | | | | | | | | | | | | | | | | | | | | TreatExp  Item 11 |  |
| **no side effects** | 0 | 1 | 2 | 3 | | 4 | | 5 | | | | 6 | | 7 | | 8 | | 9 | | 10 | | **very strong side effects** | | |  |
| **12. Constipation, loose bowels, or diarrhea** | | | | | | | | | | | | | | | | | | | | | | | | PHQ_adapt_  Item 12 |  |
| **not bothered at all** | 0 | 1 | 2 | 3 | | 4 | | 5 | | | | 6 | | 7 | | 8 | | 9 | | 10 | | **bothered a lot** | | |  |
| 12.2 How free of impairment do you expect to be from *Constipation, loose bowels, or diarrhea* by midday? | | | | | | | | | | | | | | | | | | | | | | | | NRS_expect#_  Item 12 |  |
| **not free from impairment** | 0 | 1 | 2 | 3 | | 4 | | 5 | | | | 6 | | 7 | | 8 | | 9 | | 10 | | **very free from impairment** | | |  |
| 12. Have you received or self-administered any treatment(s) for your *Constipation, loose bowels, or diarrhea* since last evening? | | | | | | | | | | | | | | | | | | | | | | | | NRS_treat1_  Item 12 |  |
| Yes | | | | | | | | | No | | | | | | | | | | | | | | | |  |
| 12.4 If yes, how much did the treatment improve your impairment from the *Constipation, loose bowels, or diarrhea*? | | | | | | | | | | | | | | | | | | | | | | | | NRS_treat2_  Item 12 |  |
| **no improvement** | 0 | 1 | 2 | 3 | | 4 | | 5 | | | | 6 | | 7 | | 8 | | 9 | | 10 | | **very strong improvement** | | |  |
| 12.5 How much did the treatment(s) worsen your impairment from the *Constipation, loose bowels, or diarrhea*? | | | | | | | | | | | | | | | | | | | | | | | | NRS_treat3_  Item 12 |  |
| **no worsening** | 0 | 1 | 2 | 3 | | 4 | | 5 | | | | 6 | | 7 | | 8 | | 9 | | 10 | | **very strong worsening** | | |  |
| 12.6 How much did you experience adverse side effects from this treatment(s)? | | | | | | | | | | | | | | | | | | | | | | | | TreatExp  Item 12 |  |
| **no side effects** | 0 | 1 | 2 | 3 | | 4 | | 5 | | | | 6 | | 7 | | 8 | | 9 | | 10 | | **very strong side effects** | | |  |
| **13. Nausea, gas, or indigestion** | | | | | | | | | | | | | | | | | | | | | | | | PHQ_adapt_  Item 13 |  |
| **not bothered at all** | 0 | 1 | 2 | 3 | | 4 | | 5 | | | | 6 | | 7 | | 8 | | 9 | | 10 | | **bothered a lot** | | |  |
| 13.2 How free of impairment do you expect to be from *Nausea, gas, or indigestion* by midday? | | | | | | | | | | | | | | | | | | | | | | | | NRS_expect#_  Item 13 |  |
| **not free from impairment** | 0 | 1 | 2 | 3 | | 4 | | 5 | | | | 6 | | 7 | | 8 | | 9 | | 10 | | **very free from impairment** | | |  |
| 13.3 Have you received or self-administered any treatment(s) for your *Nausea, gas, or indigestion* since last evening? | | | | | | | | | | | | | | | | | | | | | | | | NRS_treat1_  Item 13 |  |
| Yes | | | | | | | | | No | | | | | | | | | | | | | | | |  |
| 13.4 If yes, how much did the treatment improve your impairment from the *Nausea, gas, or indigestion*? | | | | | | | | | | | | | | | | | | | | | | | | NRS_treat2_  Item 13 |  |
| **no improvement** | 0 | 1 | 2 | 3 | | 4 | | 5 | | | | 6 | | 7 | | 8 | | 9 | | 10 | | **very strong improvement** | | |  |
| 13.5 How much did the treatment(s) worsen your impairment from the *Nausea, gas, or indigestion*? | | | | | | | | | | | | | | | | | | | | | | | | NRS_treat3_  Item 13 |  |
| **no worsening** | 0 | 1 | 2 | 3 | | 4 | | 5 | | | | 6 | | 7 | | 8 | | 9 | | 10 | | **very strong worsening** | | |  |
| 13.6 How much did you experience adverse side effects from this treatment(s)? | | | | | | | | | | | | | | | | | | | | | | | | TreatExp  Item 13 |  |
| **no side effects** | 0 | 1 | 2 | 3 | | 4 | | 5 | | | | 6 | | 7 | | 8 | | 9 | | 10 | | **very strong side effects** | | |  |
| **14. Feeling tired or having low energy** | | | | | | | | | | | | | | | | | | | | | | | | PHQ_adapt_  Item 14 |  |
| **not bothered at all** | 0 | 1 | 2 | 3 | | 4 | | 5 | | | | 6 | | 7 | | 8 | | 9 | | 10 | | **bothered a lot** | | |  |
| 14.2 How free of impairment do you expect to be from *Feeling tired or having low energy* by midday? | | | | | | | | | | | | | | | | | | | | | | | | NRS_expect#_  Item 14 |  |
| **not free from impairment** | 0 | 1 | 2 | 3 | | 4 | | 5 | | | | 6 | | 7 | | 8 | | 9 | | 10 | | **very free from impairment** | | |  |
| 14.3 Have you received or self-administered any treatment(s) for your *Feeling tired or having low energy* since last evening? | | | | | | | | | | | | | | | | | | | | | | | | NRS_treat1_  Item 14 |  |
| Yes | | | | | | | | | No | | | | | | | | | | | | | | | |  |
| 14.4 If yes, how much did the treatment improve your impairment from the *Feeling tired or having low energy*? | | | | | | | | | | | | | | | | | | | | | | | | NRS_treat2_  Item 14 |  |
| **no improvement** | 0 | 1 | 2 | 3 | | 4 | | 5 | | | | 6 | | 7 | | 8 | | 9 | | 10 | | **very strong improvement** | | |  |
| 14.5 How much did the treatment(s) worsen your impairment from the *Feeling tired or having low energy*? | | | | | | | | | | | | | | | | | | | | | | | | NRS_treat3_  Item 14 |  |
| **no worsening** | 0 | 1 | 2 | 3 | | 4 | | 5 | | | | 6 | | 7 | | 8 | | 9 | | 10 | | **very strong worsening** | | |  |
| 14.6 How much did you experience adverse side effects from this treatment(s)? | | | | | | | | | | | | | | | | | | | | | | | | TreatExp  Item 14 |  |
| **no side effects** | 0 | 1 | 2 | 3 | | 4 | | 5 | | | | 6 | | 7 | | 8 | | 9 | | 10 | | **very strong side effects** | | |  |
| **15. Trouble sleeping** | | | | | | | | | | | | | | | | | | | | | | | | PHQ_adapt_  Item 15 |  |
| **not bothered at all** | 0 | 1 | 2 | 3 | | 4 | | 5 | | | | 6 | | 7 | | 8 | | 9 | | 10 | | **bothered a lot** | | |  |
| 15.2 How free of impairment do you expect to be from *Trouble sleeping* by midday? | | | | | | | | | | | | | | | | | | | | | | | | NRS_expect#_  Item 15 |  |
| **not free from impairment** | 0 | 1 | 2 | 3 | | 4 | | 5 | | | | 6 | | 7 | | 8 | | 9 | | 10 | | **very free from impairment** | | |  |
| 15.3 Have you received or self-administered any treatment(s) for your *Trouble sleeping* since last evening? | | | | | | | | | | | | | | | | | | | | | | | | NRS_treat1_  Item 15 |  |
| Yes | | | | | | | | | No | | | | | | | | | | | | | | | |  |
| 15.4 If yes, how much did the treatment improve your impairment from the *Trouble sleeping*? | | | | | | | | | | | | | | | | | | | | | | | | NRS_treat2_  Item 15 |  |
| **no improvement** | 0 | 1 | 2 | 3 | | 4 | | 5 | | | | 6 | | 7 | | 8 | | 9 | | 10 | | **very strong improvement** | | |  |
| 15.5 How much did the treatment(s) worsen your impairment from the *Trouble sleeping*? | | | | | | | | | | | | | | | | | | | | | | | | NRS_treat3_  Item 15 |  |
| **no worsening** | 0 | 1 | 2 | 3 | | 4 | | 5 | | | | 6 | | 7 | | 8 | | 9 | | 10 | | **very strong worsening** | | |  |
| 15.6 How much did you experience adverse side effects from this treatment(s)? | | | | | | | | | | | | | | | | | | | | | | | | TreatExp  Item 15 |  |
| **no side effects** | 0 | 1 | 2 | 3 | | 4 | | 5 | | | | 6 | | 7 | | 8 | | 9 | | 10 | | **very strong side effects** | | |  |

**Complete:** *You're all done! We want to thank you for answering these questions and ask you to continue to support us in the next survey at midday. Thank you!*
