# Supplementary material for: Time-dynamic associations between symptom-related expectations, self-management experiences and somatic symptom severity in everyday life: an ecological momentary assessment study with university students
Source: BMJ Open. 2025 Feb 7;15(2):e091032. doi: 10.1136/bmjopen-2024-091032 (PMC11808919; doi:10.1136/bmjopen-2024-091032)
Supplement: Supplementary file 2 [file bmjopen-2024-091032.reviewer_comments.pdf]

PEER REVIEW HISTORY

BMJ Open publishes all reviews undertaken for accepted manuscripts. Reviewers are asked to complete a checklist review form and are provided with free text boxes to elaborate on their assessment. These free text comments are reproduced below.

ARTICLE DETAILS

Title (Provisional)

Time-dynamic associations between symptom-related expectations, self-management experiences, and somatic symptom severity in everyday life: An ecological momentary assessment study with university students

Authors

Hahn, Stefanie; Nestoriuc, Yvonne; Kirchhof, Simon; Toussaint, Anne; Löwe, Bernd; Pauls, Franz

---

VERSION 1 - REVIEW

**Reviewer**	**1**
**Name**	**Kallesøe, Karen**
**Affiliation**	**Aarhus University Hospital, Research Clinic for Functional Disorders and Psychosomatics**
**Date**	**23-Aug-2024**
**COI**	**I do not have any competing interests to declare.**

Thank you for allowing me to review the manuscript on a study investigating the associations between symptom-related expectations, self-management experiences, and expectations framing on concurrent and subsequent somatic symptom severity in university students.

The overall manuscript addresses an important issue regarding physical symptoms and potential factors influencing symptom perception, which is highly relevant given the increase in physical symptoms in the general population.

The manuscript is well written and methodologically easy to follow.

Please see my suggestions below for consideration, which aim to enhance readability and further include existing literature.

Abstract:

The abstract is very clear and concise. Please consider presenting the results in the results section in the same order as mentioned in the introduction. I suggest that you choose an order and maintain it throughout the entire manuscript to enhance readability.

Introduction:

Line 34-36 starting with “Considering the debilitating nature…”. Consider whether the aim is well-placed here in the middle of the introduction, especially since you have a separate section on objectives.

Line 48-49: The introduction would benefit from more information, including references on expectation framing, as this is also an important outcome in the manuscript.

Line 54: Please include a reference number for Henningsen et al. (2018)

Line 63-72: Consider whether the section on ecological momentary assessment should be placed in the introduction. Perhaps a condensed version of this section could be included in the methods section. This change is suggested to provide clarity and maintain a coherent flow in the introduction, which currently seems a bit fragmented as different aspects of the study are explained without necessarily linking them to each other.

The sub-section “Objective” is very long and also includes methodological components e.g. line 81 “A seven-day EMA study was conducted…”. I suggest shortening this section by omitting some of the more detailed descriptions. Some relevant points in this section could be further elaborated in the introduction section to provide context for the issues explored.

Method:

Line 160: I would suggest using a consistent way of referencing throughout the manuscript. For instance, insert reference 22 after Cronbachs alpha instead of writing Kroenke et al. This consistency will make it easier to find the specific references. The same applies to line 162, Gräfe et al.

Line 168: I suggest including a line break when moving on to symptom-related expectations even though it is the same measure.

Results:

Table 1. There seems to be some redundant information, e.g. gender. It is stated that there are 104 participants in the total sample and how this is distributed in the two groups. Could information on one gender be omitted? Since you have mentioned in the text that there is no diversity, it may be sufficient to report just one gender to improve readability. The same considerations applies to migration background and partnership.

Line 265: You have stated that a found negative interaction effect suggests that the positive association between symptom-related expectations and concurrent average levels of somatic symptom severity was slightly more pronounced for participants in the negative expectation framing group than for those in the positive expectation framing group. Please consider whether this statement would be more appropriate for the discussion section.

Discussion

The topics discussed in this section are relevant. However, I suggest putting your current findings more into context with the existing literature, e.g. what others have found about self-management and symptom-related expectations. This applies to the entire discussion section.

Conclusion

This section is very long and is not only summarizes the findings but also includes an extended section on micro-interventions. I suggest shortening this to one or two sentences regarding future perspectives.

**Reviewer**	**2**
**Name**	**Hennemann, Severin**
**Affiliation**	**University of Mainz, Dep. of Clinical Psychology, Psychotherapy and Experimental Psychopathology**
**Date**	**28-Aug-2024**
**COI**	**None to declare.**

The manuscript describes a randomized, 7-day EMA study testing the effect of positive or negative expectation framing on the bothersomeness of somatic symptoms in a convenience sample of 104 university students. The role of expectation, i.e. placebo and nocebo effects on symptom perception in everyday life is an important research agenda and the manuscript is concise and overall methodologically sound. However, some points should be addressed by the authors in a revision:

Abstract

You could add a short information on the framing conditions.

Use “showed” or “indicated” rather than “suggested”.

Intro

Reasons for the particular vulnerability for somatic symptoms/PSS in young/emerging adults are lacking.

Please elaborate on the methods and evidence of previous EMA studies in PSS/FSS/somatoform disorders and identify the research gap.

p.6, l. 68 “devices” not “devises”

Methods

The EMA method used here has some shortcomings. The short assessment period and relatively few assessment points per day (e.g. May et al., 2018, doi: 10.1016/j.jpain.2018.01.006) should be discussed critically, as is the use of fixed time intervals, which may increase missingness (as opposed to somewhat more frequently used stratified randomized time periods).

One could argue that the PHQ-15 is rather intended to assess somatic symptom distress (or bothersomeness) than severity (e.g., Witthöft et al., 2013, DOI 10.1007/s12529-012-9237-2), yet this is a rather scholarly topic beyond the scope of this manuscript (feel free to discuss).

Why was the study, which is part of a larger, publicly funded project, not pre-registered? At least, I could not find any info on that aspect (also, the link on p. 20 was not working).

Since no trait measures (i.e. self-reports) were applied at baseline, valuable information on the sample is missing. Another option could have been a synthetic control group (i.e. non-instructed EMA data collection phase beforehand).

Did you use verbal anchors for the interim scale points of the adapted PHQ-15 (the original has verbals on each scale point)? See also my comment on the missing reliability of this version.

Did participants receive any reimbursement?

Did you test gender differences in symptom reporting? Your sample includes a rather high proportion of male psych students which could be interesting, given that most studies in this group include mostly female participants. Could this be a potential covariate to your analyses?

Were participants randomized in a 1:1 ratio?

Fig. 1 shows that all randomized participants were analyzed (fulfilling the ITT principle). This is a strength of the study, however, It just made me wonder, why there were no technical dropouts, which occur frequently in EMA studies.

How was the first (morning) measurement point treated in analyses (predicted by evening, i.e. overnight effects)?

Did you intervene (e.g. messaging participants) in case of low completion rates? I noticed, that you also included participants with less than 10% of data, can you tell how robust MLM is for that amount of missingness per person?

Were there any statistical outliers and if so, how were these treated? It could be interesting to repeat MLM without outliers as a sensitivity analysis.

Please substantiate medium-sized between-group effects for the power calculation. Why were auto-regressive effects of symptom severity not included here?

There is no supplementary information provided, which is rather unusual. For example, I would like to see the adapted questionnaire, an example for assessment within the app, further interaction effects of MLMs, sensitivity/subgroup analyses if applicable, etc.

Results

Please report the reliabilities of the scales used in the EMA phase.

Please add average values for IVs and perhaps also group testing for average values.

Were there any high symptom reporters in the sample? A subgroup analysis could shed light on the mechanisms of PSS or SSD and indicate whether contradictory framing led to increased symptoms for those who are vulnerable.

Did the framing effects accumulate over time, that is, have you inspected individual slopes (ICCs indicated some variability here)?

While I find the interpretation that nocebo effects may have a different latency than placebo effects, the data do not show a significant interaction effect, right? Thus, the explanation should be more cautious.

The intro and discussion section should elaborate more on existing empirical evidence. For example, various EMA studies point towards a strong predictive of negative affect (e.g., Hennemann et al., 2023, doi: 1016/j.jpsychores.2023.111429) or other psychobehavioral aspects (e.g., Mewes et a., 2022). Furthermore, beyond the effect of selective attention, induced by the expectation framing, other anteceding mechanisms of symptom perception such as those described in predictive processing theories (e.g., Van den Bergh, et al., 2017; DOI: 10.1016/j.neubiorev.2017.01.015) could be addressed.

VERSION 1 - AUTHOR RESPONSE

Reviewer: 1
Dr. Karen Kallesøe, Aarhus University Hospital

Comments to the Author:
Thank you for allowing me to review the manuscript on a study investigating the associations between symptom-related expectations, self-management experiences, and expectations framing on concurrent and subsequent somatic symptom severity in university students.
The overall manuscript addresses an important issue regarding physical symptoms and potential

factors influencing symptom perception, which is highly relevant given the increase in physical symptoms in the general population.

The manuscript is well written and methodologically easy to follow.
Please see my suggestions below for consideration, which aim to enhance readability and further include existing literature.

Abstract:
- The abstract is very clear and concise. Please consider presenting the results in the results section in the same order as mentioned in the introduction. I suggest that you choose an order and maintain it throughout the entire manuscript to enhance readability.
Authors' response: We are grateful for the valuable comments received from the reviewer. We have now revised the results section of the abstract for the sake of readability (page 2, lines 21-32). Please note that additional changes to the abstract have been made based on the editor's comments.

Introduction:
- Line 34-36 starting with "Considering the debilitating nature...". Consider whether the aim is well-placed here in the middle of the introduction, especially since you have a separate section on objectives.
Authors' response: Thank you for this helpful remark. We fully agree that the aim of the study was slightly misplaced at the end of the introduction. Therefore, we have now revised the introduction section and excluded the misplaced sentence accordingly (page 4, lines 66-70).

- Line 48-49: The introduction would benefit from more information, including references on expectation framing, as this is also an important outcome in the manuscript.
Authors' response: We have now included more information regarding expectation framing as well as references related to that topic in the introduction section (page 5, lines 84-88).

- Line 54: Please include a reference number for Henningsen et al. (2018)
Authors' response: We have now included a reference number for Henningsen et al. (2018) (page 5, line 94).

- Line 63-72: Consider whether the section on ecological momentary assessment should be placed in the introduction. Perhaps a condensed version of this section could be included in the methods section. This change is suggested to provide clarity and maintain a coherent flow in the introduction, which currently seems a bit fragmented as different aspects of the study are explained without necessarily linking them to each other.
Authors' response: We appreciate the suggestion given by the reviewer. Although our initial aim was to introduce the EMA in the introduction section before providing study-specific information in the methods section later on, we agree that a brief introduction of the EMA approach is better placed in the methods section for coherence (page 6, lines 137-142). Therefore, we have now adjusted both sections accordingly in order to improve the flow and clarity of our manuscript.

- The sub-section "Objective" is very long and also includes methodological components e.g. line 81 "A seven-day EMA study was conducted...". I suggest shortening this section by omitting some of the

more detailed descriptions. Some relevant points in this section could be further elaborated in the introduction section to provide context for the issues explored.
Authors' response: Thank you for this suggestion. The corresponding sentence has now been removed from the introduction and included in the methods section (page 6, lines 137-142). Apart from that, however, we still think that the sub-section "Objective" is reasonable in length as it not only gives on overview of the main study goals but also includes the study results we expected to find based on the information provided in the introduction section.

Method:
- Line 160: I would suggest using a consistent way of referencing throughout the manuscript. For instance, insert reference 22 after Cronbachs alpha instead of writing Kroenke et al. This consistency will make it easier to find the specific references. The same applies to line 162, Gräfe et al.
Authors' response: Thank you for pointing this out. We have now checked for the consistency of referencing throughout the entire manuscript and revised all references accordingly (page 7, line 183).

- Line 168: I suggest including a line break when moving on to symptom-related expectations even though it is the same measure.
Authors' response: We have now included a line break before describing the symptom-related expectations measures (page 7, lines 189-190) and another line break when moving on to the self-treatment experience measure (page 8, lines 203-204).

Results:
- Table 1. There seems to be some redundant information, e.g. gender. It is stated that there are 104 participants in the total sample and how this is distributed in the two groups. Could information on one gender be omitted? Since you have mentioned in the text that there is no diversity, it may be sufficient to report just one gender to improve readability. The same considerations applies to migration background and partnership.
Authors' response: We appreciate the suggestion given by the reviewer. We agree that table 1. included some redundant information. In accordance with this comment and further feedback provided by the editorial office, we have thus revised the table to improve readability. The manuscript now includes separate Tables on (1) sociodemographic and (2) health care-related information for the complete sample (see Table 1 and 2, pages 10 and 11). For further information on the expectation framing groups and group comparison we have provided an additional Table in the supplements (see Table b).

- Line 265: You have stated that a found negative interaction effect suggests that the positive association between symptom-related expectations and concurrent average levels of somatic symptom severity was slightly more pronounced for participants in the negative expectation framing group than for those in the positive expectation framing group. Please consider whether this statement would be more appropriate for the discussion section.
Authors' response: Since the mentioned statement already provides an interpretation of the results in some sense, we agree that it would be more appropriate for the discussion section. Therefore, we have now revised the corresponding sentence in the results section (page 12, lines 287-289) and included another sentence related to the interaction effect in the discussion section (page 15, lines

346-350).

Discussion:
- The topics discussed in this section are relevant. However, I suggest putting your current findings more into context with the existing literature, e.g., what others have found about self-management and symptom-related expectations. This applies to the entire discussion section.
Authors' response: Thank you for requesting more context with the existing literature on this topic. We agree that this section could profit from additional references and have therefore added more references for the discussion and expanded several topics in the discussion (page 14, lines 337-340, page 15, lines 346-350, page 16, lines 407-409).

Conclusion:
- This section is very long and is not only summarizes the findings but also includes an extended section on micro-interventions. I suggest shortening this to one or two sentences regarding future perspectives.
Authors' response: Thank you for this remark. We have now shortened the according section on future perspectives (page 17, lines 447-450).

Reviewer: 2
Dr. Severin Hennemann, University of Mainz

Comments to the Author:
The manuscript describes a randomized, 7-day EMA study testing the effect of positive or negative expectation framing on the bothersomeness of somatic symptoms in a convenience sample of 104 university students. The role of expectation, i.e. placebo and nocebo effects on symptom perception in everyday life is an important research agenda and the manuscript is concise and overall methodologically sound. However, some points should be addressed by the authors in a revision:

Abstract

- You could add a short information on the framing conditions.
Authors' response: Thank you for your suggestion. We have now included a short sentence in the methods section of the abstract to provide more information on the framing conditions (page 2, lines 14-16).

Use "showed" or "indicated" rather than "suggested".
Authors' response: We have now replaced the word "suggested" by the word "showed" in the according sentence of the abstract (page 2, line 27).

Intro

- Reasons for the particular vulnerability for somatic symptoms/PSS in young/emerging adults are lacking.
Authors' response: Thank you for requesting additional background information. We agree that additional information for the vulnerability of this particular group is relevant and have thus

provided this information in the introduction (page 4, lines 50-53).

- Please elaborate on the methods and evidence of previous EMA studies in PSS/FSS/somatoform disorders and identify the research gap.
Authors' response: Thank you for requesting evidence of previous EMA studies on similar topics. We have now included more information on previous studies focusing on PSS/FSS/somatoform disorders and identified the specific gap addressed in our study (page 4, lines 63-66). Since we have followed Reviewer 1's recommendation to introduce EMA in the section "study design", we have now updated our references on the implementation of this method in previous studies within this new section (page 6, line 143).

- p.6, l. 68 "devices" not "devises"
Authors' response: Thank you for this remark. As the specific section has been shortened, the word in question is no longer included in the according section of the main document.

Methods

- The EMA method used here has some shortcomings. The short assessment period and relatively few assessment points per day (e.g. May et al., 2018, doi: 10.1016/j.jpain.2018.01.006) should be discussed critically, as is the use of fixed time intervals, which may increase missingness (as opposed to somewhat more frequently used stratified randomized time periods).
Authors' response: Thank you for pointing out these shortcomings. We agree that EMA data based on stratified randomized assessment points would likely have increased ecological validity and have thus included this point as a limitation of our study (page 16, lines 414-416). However, we decided to use predetermined assessment points to facilitate meaningful comparisons without having to control for time and to avoid being too dependent on later analytical procedures. Please also note that we did not explicitly hypothesize time as a predictor of symptom severity in the present study. However, investing time-related effects would be an interesting goal for future research.

- One could argue that the PHQ-15 is rather intended to assess somatic symptom distress (or bothersomeness) than severity (e.g., Witthöft et al., 2013, DOI 10.1007/s12529-012-9237-2), yet this is a rather scholarly topic beyond the scope of this manuscript (feel free to discuss).
Authors' response: We agree that there is still an ongoing debate on whether the PHQ-15 assesses symptom distress, burden, or severity. Our decision to refer to the outcome as "symptom severity" is based on several reasons. On one hand, initial publications for the PHQ-15 referred to the outcome as symptom severity (Kroenke et al. 2002, https://doi.org/10.1097/00006842-200203000-00008). The contextual understanding of the outcome was furthermore discussed in a systematic review concerning the assessment of common somatic symptoms in large-scale studies (Zijlema et al., 2013, https://doi.org/10.1016/j.jpsychores.2013.03.093). We therefore concluded, in line with principle investigators of the main project as well as the advisory board of the associated research unit SOMACROSS (RU5111), that the PHQ-15 sum score should be used to operationalize the overall somatic symptom severity.

- Why was the study, which is part of a larger, publicly funded project, not pre-registered? At least, I could not find any info on that aspect (also, the link on p. 20 was not working).
Authors' response: Thank you for this important comment regarding the pre-registration of the

study. While pre-registration is generally considered good practice for enhancing transparency and reducing biases in research, we did not conduct a pre-registration. As this present study acted as a pilot study for the pre-registered SOMA.SSD-study (ISRCTN36251388) and applied the same EMA methodology and study design on a micro-level, an additional pre-registration was not deemed necessary. We apologize for the faulty link and have updated it accordingly.

- Since no trait measures (i.e. self-reports) were applied at baseline, valuable information on the sample is missing. Another option could have been a synthetic control group (i.e. non-instructed EMA data collection phase beforehand).
Authors' response: Thank you for pointing this out. We do agree that additional information on the sample could be valuable. Especially the investigation of psychological factors such as depression, anxiety or negative affectivity are therefore interesting elements that will be included as covariates, moderators, or potential predictor variables in future research within SOMACROSS. However, given that the main objectives of this study focused on symptom-related expectations and symptom severity, we decided not to assess further (psychological) factors in addition to socio-demographic data in order to ensure the most economical survey possible. As we do agree that these factors should be considered in the future, we have addressed their importance in our discussion (page 16, lines 407-409)

- Did you use verbal anchors for the interim scale points of the adapted PHQ-15 (the original has verbals on each scale point)? See also my comment on the missing reliability of this version.
Authors' response: Thank you for requesting further clarification. Since our goal was to ensure the measures used for symptom-related expectations and somatic symptom severity to be as analogous as possible, we decided to adapt the original PHQ-15 items to a 11-point numeric rating scale with two verbal anchors for the adapted PHQ-15, thus matching the scale points and anchors of the 11-point numeric rating scale used for symptom-related expectation. This enabled a point-by-point comparison between both measures. However, you are correct that this adapted version of the PHQ-15 was developed due to the described pragmatic reasons and has not yet been validated. Please note that we have now included the entire questionnaire including items for both measures PHQ_{adapt} as supplementary material for further clarification.

- Did participants receive any reimbursement?
Authors' response: Thank you for requesting further clarification. Participants did not receive financial reimbursement. Instead, Instead, they received university course credit points (i.e., three test subject hours) for a fully completed study participation. We have now highlighted this information in the methods section (page 6, lines 134-136).

- Did you test gender differences in symptom reporting? Your sample includes a rather high proportion of male psych students which could be interesting, given that most studies in this group include mostly female participants. Could this be a potential covariate to your analyses?
Authors' response: Thank you for this suggestion. Given the high proportion of male students in our study sample, we fully agree on the importance of controlling for potential gender effects. Therefore, we conducted a preliminary sensitivity analysis prior to our main analyses to investigate potential gender effects on our predictor variables symptom-related expectations and self-management experiences as well as on our outcome variable somatic symptom severity. This analysis indicated no statistically significant gender differences. Since there was no indication for

gender to be a potential covariate, we decided to not report results of this sensitivity analysis for reasons of space.

- Were participants randomized in a 1:1 ratio?
Authors' response: Yes, participants were randomized in a 1:1 ratio. Please note that random group allocation in a 1:1 ratio was ensured by providing participants with random group specific QR codes for study registration. Prior to assessment, the same number of QR-Codes had been created for each framing group and were randomly distributed among participants in predefined alternating order. For clarification, we have now included this information in the methods section (page 6, lines 147-149).

- Fig. 1 shows that all randomized participants were analyzed (fulfilling the ITT principle). This is a strength of the study, however, It just made me wonder, why there were no technical dropouts, which occur frequently in EMA studies.
Authors' response: Thank you for requesting further clarification on the dropout statistics. Indeed, Figure 1 illustrates that a total of *n* = 32 participants had to be excluded from data collection due to not providing smartphone operating systems compatible with the study application, unavailability and not responding, or broken devices. Please note that after installation and study registration, there were no cases in which technical issues (e.g., smartphone being turned off during assessment points) lead to a drop out. However, apart from non-compliance with the study-specific instructions provided in advance, technical problems were the main reason for missing data.

- How was the first (morning) measurement point treated in analyses (predicted by evening, i.e. overnight effects)?
Authors' response: In our time-lagged analysis (MLM 2), associations were analyzed between somatic symptom severity at a given assessment point (t) as the criterion variable and somatic symptom severity, symptom-related expectations, self-management experiences, expectation framing, and the interaction between symptom-related expectations and expectation framing at the previous assessment point (t-1) as the predictor variables. Given that the assessment points for all predictor variables included were lagged this way, the very first assessment point for the outcome variable somatic symptom severity was not included in the time-lagged analysis. However, somatic symptom severity was also treated as a time-lagged predictor variable to analyze the effects of prior symptom severity on subsequent symptom severity.
Since possible time effects were not examined in our study, we fully agree that potential overnight effects on all (morning) assessment points could have been neglected. Also, time intervals between the last assessment point on each day (evening) and the first assessment point on each following day (morning) were longer than time intervals between the assessment points during each day. Since an in-depth investigation of possible time effects was beyond the scope of the present study, we have now included a brief recommendation for future studies in the discussion (page 16, lines 414-419).

- Did you intervene (e.g. messaging participants) in case of low completion rates?
Authors' response: Thank you for this question. We did not intervene in cases of low completion rates. Contact to participants was established via automated emails (e.g. after completing the sociodemographic questionnaire), which were intended to serve as reminders for study participation. In cases of severe technical issues, however, participants could receive individual technical support via email as well. To strengthen compliance, participants were informed before

participation (e.g., informed consent) that the number of credits (test subject hours) they would receive for their participation would depend on the compliance rate.

- I noticed, that you also included participants with less than 10% of data, can you tell how robust MLM is for that amount of missingness per person?
Authors' response: Thank you for requesting further clarification. It has to be noted that there were only *n* = 1 cases with a compliance rate less than 10 % in the total sample under examination. Please also note that the present MLM analyses were based on maximum likelihood estimation, which is known to be a robust an appropriate method to account for missing values in extensive longitudinal data. This is because missing data are not actually imputed but the model is estimated making use of the incomplete data in a way that does not bias estimates under certain conditions. The process is comparable to the full maximum likelihood approach in structural equation modeling. Missing data estimation in this sense produces unbiased estimation of the complete data and often even better estimation than if conventional missing data imputation techniques would have been used.

- Were there any statistical outliers and if so, how were these treated? It could be interesting to repeat MLM without outliers as a sensitivity analysis.
Authors' response: Thank you for this important comment. In our main analysis, we included statistical outliers as we wanted to include all responses in an unaltered analysis. However, we did check for statistical outliers and were able to identify *N* = 6 (*n* =2 in the negative expectation framing group, *n* = 4 in the positive expectation framing group) outliers for both symptom severity and symptom-related expectations and *N* = 5 (*n* = 1 in the negative expectation framing group, *n* = 4 in the positive expectation framing group) for self-management experiences. We also conducted a sensitivity analysis and repeated MLM 1 and MLM 2 without outliers. We have now provided the corresponding tables as supplementary materials as well as a short description in our manuscript (pages 13-14, lines 304-312).

- Please substantiate medium-sized between-group effects for the power calculation. Why were auto-regressive effects of symptom severity not included here?
Authors' response: Thank you for requesting further clarification. As there is no sufficient research on the time-dynamic associations between symptom-related expectations, expectation framing, self-management experiences, and somatic symptom severity in a nonclinical sample of university students, there is no prior knowledge from which to draw for an a priori case number estimation. However, placebo/nocebo responses, research often suggests medium effect sizes (Cohen's d ≈ 0.5) regarding their impact on outcomes (e.g., Enck et al. 2013, DOI: 10.1038/nrd3923; Price et al. 2008, DOI: 10.1146/annurev.psych.59.113006.095941). Accordingly, statistical power and the required sample size estimations were based on an approximative power analysis with the latter being based on a medium effect size, an alpha error of $\alpha = 0.05$, a statistical power of $1-\beta = 0.80$, and four predictor variables, suggesting a required total sample size of *N* = 85 for the variance in the criterion variable (somatic symptom severity) to be different from zero. Given the large number of observations at level 1 that are nested within individuals at level 2, the sample size analyzed in MLM 1 and MLM 2 is in line with recommendations regarding statistical power estimation in two-level models (Arend & Schäfer, 2019, https://doi.org/10.1037/met0000195).
Furthermore, it has to be noted that we conducted preliminary analyses including an examination of the fit of different covariance structures. The model with the best fit (according to AIC, -2log) for

MLM 1 and MLM 2 was the AR(1) model, in which autoregressive effects were also taken into account. However, results of the preliminary analyses (e.g., model comparisons) were not presented in the manuscript for reasons of space.

- There is no supplementary information provided, which is rather unusual. For example, I would like to see the adapted questionnaire, an example for assessment within the app, further interaction effects of MLMs, sensitivity/subgroup analyses if applicable, etc.
Authors' response: Thank you for suggesting the inclusion of further supplementary information. We have now included an additional table providing descriptive statistics for the measures, tables for additional sensitivity analyses, and the adapted questionnaire as supplementary documents.

Results

- Please report the reliabilities of the scales used in the EMA phase.
Authors' response: Thank you for requesting further clarification. We have now included an additional table as supplementary material, in which Cronbach's alpha values are provided for each scale used during the EMA.

- Please add average values for IVs and perhaps also group testing for average values.
Authors' response: Thank you for this request. Please note that we have now included an additional table as supplementary material, in which descriptive statistics (e.g., means and standard deviations) are provided for the requested information.

- Were there any high symptom reporters in the sample? A subgroup analysis could shed light on the mechanisms of PSS or SSD and indicate whether contradictory framing led to increased symptoms for those who are vulnerable.
Authors' response: Thank you for highlighting this important question. We did conduct a screening for high-symptom reporters and were able to identify a total of N = 6 (n =2 in the negative expectation framing group, n = 4 in the positive expectation framing group) participants. Since our sample does not include a reasonable number of high symptom reporters to conduct a meaningful subgroup analysis, we have now included this important aspect as an implication for future research. As we fully agree that investigations on extremely vulnerable individuals could be an interesting approach to further investigate the mechanisms of PSS or SSD, we will take this as a valuable input for future projects and analyses (pages 16-17, lines 426-430) .

- Did the framing effects accumulate over time, that is, have you inspected individual slopes (ICCs indicated some variability here)?
Authors' response: Thank you for raising a question on this important issue. As potential accumulative effects of framing over time were not part of the objectives of the present study, we did not control for such. However, we agree that this would be an important and interesting research topic to investigate and have therefore addressed this topic as an implication for future research (page 17, lines 430-434).

- While I find the interpretation that nocebo effects may have a different latency than placebo effects, the data do not show a significant interaction effect, right? Thus, the explanation should be more cautious.

Authors' response: Thank you for this comment. As we agree that the interpretation of the results in question should be done more cautiously, the corresponding section has now been revised accordingly (page 16, lines 390-394).

- The intro and discussion section should elaborate more on existing empirical evidence. For example, various EMA studies point towards a strong predictive of negative affect (e.g., Hennemann et al., 2023, doi: 1016/j.jpsychores.2023.111429) or other psychobehavioral aspects (e.g., Mewes et a., 2022). Furthermore, beyond the effect of selective attention, induced by the expectation framing, other anteceding mechanisms of symptom perception such as those described in predictive processing theories (e.g., Van den Bergh, et al., 2017; DOI: 10.1016/j.neubiorev.2017.01.015) could be addressed.

Authors' response: Thank you for this comment and your suggestions. We have now expanded the introduction accordingly (page 4, lines 63-66). Additionally, we have incorporated more empirical evidence in the discussion, including predictive processing theories (page 15, lines 355-367, page 15, lines 374-377, page 16 lines 407-409).

VERSION 2 - REVIEW

Reviewer 1

Name Kallesøe, Karen

Affiliation Aarhus University Hospital, Research Clinic for Functional Disorders and Psychosomatics

Date 16-Dec-2024

COI

Thank you for allowing me to review the revised manuscript. The authors have thoroughly addressed the questions raised during the review process and I fully support its publication with no further revisions needed.

Reviewer 2

Name Hennemann, Severin

Affiliation University of Mainz, Dep. of Clinical Psychology, Psychotherapy and Experimental Psychopathology

Date 02-Dec-2024

COI

The authors have thoroughly addressed the reviewers' comments, resulting in significant improvements to the manuscript. I have only a few minor suggestions remaining:

Study characterization: In the rebuttal letter, you describe your study as a pilot for a larger upcoming project. However, this is not clearly reflected in the manuscript or its title. I recommend explicitly stating this.

Presentation of results: Consider structuring the results section alongside your hypotheses to enhance clarity and readability.

Missingness: For transparency, please report the average level (or range) of missingness in the EMA data.

VERSION 2 - AUTHOR RESPONSE

Reviewer: 1

Dr. Karen Kallesøe, Aarhus University Hospital

Comments to the Author:

Thank you for allowing me to review the revised manuscript. The authors have thoroughly addressed the questions raised during the review process and I fully support its publication with no further revisions needed.

Reviewer: 2

Dr. Severin Hennemann, University of Mainz

Comments to the Author:

The authors have thoroughly addressed the reviewers' comments, resulting in significant improvements to the manuscript. I have only a few minor suggestions remaining:

(1) Study characterization: In the rebuttal letter, you describe your study as a pilot for a larger upcoming project. However, this is not clearly reflected in the manuscript or its title. I recommend explicitly stating this.

Authors response: Thank you for this suggestion. We have now stated the pilot character of the present study more explicitly in the study design section (page 7, lines 172-176).

(2) Presentation of results: Consider structuring the results section alongside your hypotheses to enhance clarity and readability.

Authors response: Thank you for pointing out this opportunity for further improvement. As our goal is to provide a clearly written and readable manuscript, we have now adjusted the structure of the objectives and results (page 5 & 6, lines 106-110, 115-119, 123-124).

(3) Missingness: For transparency, please report the average level (or range) of missingness in the EMA data.

Authors response: Thank you for this suggestion. We have now included the requested information on the average level of missingness in the EMA data (page 9, lines 271 & 272).
